# Effect of second booster vaccinations and prior infection against SARS-CoV-2 in the UK SIREN healthcare worker cohort

**DOI:** 10.1016/j.lanepe.2023.100809

**Published:** 2023-12-14

**Authors:** Peter D. Kirwan, Victoria J. Hall, Sarah Foulkes, Ashley D. Otter, Katie Munro, Dominic Sparkes, Anna Howells, Naomi Platt, Jonathan Broad, David Crossman, Chris Norman, Diane Corrigan, Christopher H. Jackson, Michelle Cole, Colin S. Brown, Ana Atti, Jasmin Islam, SIREN Study Group, Tracy Lewis, Steve Bain, Rebeccah Thomas, John Geen, Carla Pothecary, Sean Cutler, John Northfield, Cathy Price, Johanne Tomlinson, Sarah Knight, Emily Macnaughton, Ekaterina Watson, Rajeka Lazarus, Aaran Sinclair, Joanne Galliford, Bridgett Masunda, Tabitha Mahungu, Alison Rodger, Esther Hanison, Simon Warren, Swati Jain, Mariyam Mirfenderesky, Natasha Mahabir, Rowan Pritchard-Jones, Diane Wycherley, Claire Gabriel, Elijah Matovu, Philippa Bakker, Simantee Guha, S. Gormley, James Pethick, Georgina Butt, Stacey Pepper, Luke Bedford, Paul Ridley, Jane Democratis, Manjula Meda, Anu Chawla, Fran Westwell, Nagesh Kalakonda, Sheena Khanduri, Allison Doel, Sumita Pai, Christian Hacon, Davis Nwaka, Veronica Mendez Moro, A. Moody, Cressida Auckland, Stephanie Prince, Thushan de Silva, Helen Shulver, A. Shah, C. Jones, Banerjee Subhro-Osuji, Angela Houston, Tim Planche, Martin Booth, Christopher Duff, Jonnie Aeron-Thomas, Ray Chaudhuri, David Hilton, Hannah Jory, Zehra'a Al-Khafaji, Philippa Kemsley, Ruth Longfellow, David Boss, Simon Brake, Louise Coke, Ngozi Elumogo, Scott Latham, Chinari Subudhi, Ina Hoad, Claire Thomas, Nihil Chitalia, Tracy Edmunds, Helen Ashby, John Elliott, Beverley Wilkinson, Abby Rand, Catherine Thompson, K. Agwuh, Anna Grice, Kelly Moran, Vijayendra Waykar, Yvonne Lester, Lauren Sach, Kathryn Court, Nikki White, Clair Favager, Kyra Holliday, Jayne Harwood, Brendan Payne, Karen Burns, Lynda Fothergill, Alejandro Arenas-Pinto, Abigail Severn, Kerryanne Brown, Katherine Gray, Jane Dare, Qi Zheng, Kathryn Hollinshead, Robert Shorten, Alun Roebuck, Christopher Holmes, Martin Wiselka, Barzo Faris, Liane Marsh, Cressida Auckland, Clare McAdam, Lisa Ditchfield, Zaman Qazzafi, G. Boyd, N. Wong, Sarah Brand, Jack Squires, John Ashcroft, Ismaelette Del Rosario, Joanne Howard, Emma Ward, Gemma Harrison, Joely Morgan, Claire Corless, Robert Shorten, Ruth Penn, Nick Wong, Manny Bagary, Nadezda Starkova, Mandy Beekes, Mandy Carnahan, Shivani Khan, Shekoo Mackay, Keneisha Lewis, Graham Pickard, Joy Dawson, Lauren Finlayson, Euan Cameron, Anne Todd, Sebastien Fagegaltier, Sally Mavin, Alexandra Cochrane, Andrew Gibson, Sam Donaldson, Kate Templeton, Martin Malcolm, Beth Smith, Devesh Dhasmana, Susan Fowler, Antonia Ho, Michael Murphy, Claire Beith, Manish Patel, Elizabeth Boyd, Val Irvine, Alison Grant, Rebecca Temple-Purcell, Clodagh Loughrey, Elinor Hanna, Frances Johnston, Angel Boulos, Fiona Thompson, Yuri Protaschik, Susan Regan, Tracy Donaghy, Maurice O'Kane, Omolola Akinbami, Paola Barbero, Tim Brooks, Meera Chand, Ferdinando Insalata, Palak Joshi, Anne-Marie O'Connell, Mary Ramsay, Ayoub Saei, Maria Zambon, Ezra Linley, Simon Tonge, Enemona Adaji, Omoyeni Adebiyi, Nick Andrews, Joanna Conneely, Paul Conneely, Angela Dunne, Simone Dyer, Hannah Emmett, Nipunadi Hettiarachchi, Nishanthan Kapirial, Jameel Khawam, Edward Monk, Sophie Russell, Andrew Taylor-Kerr, Jean Timeyin, Silvia D'Arcangelo, Cathy Rowe, Amanda Semper, Eileen Gallagher, Robert Kyffin, Lisa Cromey, Desmond Areghan, Jennifer Bishop, Melanie Dembinsky, Laura Dobbie, Josie Evans, David Goldberg, Lynne Haahr, Annelysse Jorgenson, Ayodeji Matuluko, Laura Naismith, Desy Nuryunarsih, Alexander Olaoye, Caitlin Plank, Lesley Price, Nicole Sergenson, Sally Stewart, Andrew Telfer, Jennifer Weir, Ellen De Lacy, Yvette Ellis, Susannah Froude, Guy Stevens, Linda Tyson, Susanna Dunachie, Paul Klenerman, Chris Duncan, Rebecca Payne, Lance Turtle, Alex Richter, Thushan De Silva, Eleanor Barnes, Daniel Wootton, Oliver Galgut, Jonathan Heeney, Helen Baxendale, Javier Castillo-Olivares, Rupert Beale, Edward Carr, Wendy Barclay, Maya Moshe, Massimo Palmarini, Brian Willett, John Kenneth Baillie, Jennie Evans, Erika Aquino, Anne M. Presanis, Andre Charlett, Daniela De Angelis, Susan Hopkins

**Affiliations:** a MRC Biostatistics Unit, University of Cambridge, United Kingdom; b UK Health Security Agency, United Kingdom; c School of Medicine, University of St Andrews, United Kingdom; d Health and Care Research Wales, United Kingdom; e Northern Ireland Public Health Agency, United Kingdom

**Keywords:** SARS-CoV-2, Vaccine effectiveness, Asymptomatic, Symptomatic, Healthcare worker, Cohort study

## Abstract

**Background:**

The protection of fourth dose mRNA vaccination against SARS-CoV-2 is relevant to current global policy decisions regarding ongoing booster roll-out. We aimed to estimate the effect of fourth dose vaccination, prior infection, and duration of PCR positivity in a highly-vaccinated and largely prior-COVID-19 infected cohort of UK healthcare workers.

**Methods:**

Participants underwent fortnightly PCR and regular antibody testing for SARS-CoV-2 and completed symptoms questionnaires. A multi-state model was used to estimate vaccine effectiveness (VE) against infection from a fourth dose compared to a waned third dose, with protection from prior infection and duration of PCR positivity jointly estimated.

**Findings:**

1298 infections were detected among 9560 individuals under active follow-up between September 2022 and March 2023. Compared to a waned third dose, fourth dose VE was 13.1% (95% CI 0.9 to 23.8) overall; 24.0% (95% CI 8.5 to 36.8) in the first 2 months post-vaccination, reducing to 10.3% (95% CI −11.4 to 27.8) and 1.7% (95% CI −17.0 to 17.4) at 2–4 and 4–6 months, respectively. Relative to an infection >2 years ago and controlling for vaccination, 63.6% (95% CI 46.9 to 75.0) and 29.1% (95% CI 3.8 to 43.1) greater protection against infection was estimated for an infection within the past 0–6, and 6–12 months, respectively. A fourth dose was associated with greater protection against asymptomatic infection than symptomatic infection, whilst prior infection independently provided more protection against symptomatic infection, particularly if the infection had occurred within the previous 6 months. Duration of PCR positivity was significantly lower for asymptomatic compared to symptomatic infection.

**Interpretation:**

Despite rapid waning of protection, vaccine boosters remain an important tool in responding to the dynamic COVID-19 landscape; boosting population immunity in advance of periods of anticipated pressure, such as surging infection rates or emerging variants of concern.

**Funding:**

UK Health Security Agency, 10.13039/501100000265Medical Research Council, NIHR HPRU Oxford, Bristol, and others.

**Research in context**

**Evidence before this study**

We searched Ovid Embase and MEDLINE for articles in English published between 1 January 2022 and 22 September 2023 using the keywords (vaccin∗ OR immunis∗ OR immuniz∗ OR mRNA OR spikevax OR comirnaty) AND (coronavirus OR sars-cov-2 OR sarscov2 OR severe acute respiratory syndrome coronavirus 2 OR COVID OR COVID-19) AND (effectiveness OR vaccine effectiveness OR VE) AND (omicron OR BA.4 OR BA.5 OR XBB), limited to “human” studies. We selected articles that included vaccine efficacy of mRNA fourth dose boosters against infection in the Omicron BA.4/5 and/or XBB sub-lineage circulating-period. Relatively few studies have investigated the real-world effectiveness of booster doses, and study designs vary, with none collecting data on asymptomatic infection. A test-negative-style study in US pharmacies estimated fourth booster vaccine effectiveness (VE) against symptomatic BA.5 and XBB/XBB.1.5 SARS-CoV-2 infection of around 50% during the initial post-vaccination period, with some waning of protection from 2 months post-vaccination among older study participants. These estimates were unadjusted for infection history and the authors acknowledge several other biases in the study design which could not be accounted for. Meanwhile, a Dutch cohort study of self-reported SARS-CoV-2 infection estimated fourth booster VE among 60 to 85 year-olds of 14% (95% CI 1 to 25%), with greater protection from recent prior infection compared to booster vaccination, and minimal evidence of waning vaccine protection in the limited period of post-vaccination follow-up.

**Added value of this study**

This study uses an established cohort of UK National Health Service (NHS) healthcare workers with dense sampling of PCR and antibody status, combined with regular self-reported symptom information. Controlling for prior infection, we estimate the effectiveness of a fourth dose mRNA vaccine against infection (both symptomatic and asymptomatic) over the period September 2022 to March 2023 of 24% in the first 2 months post-vaccination, with significant waning of protection thereafter. Protection from recent prior infection is estimated to be greater and longer-lasting, as compared to booster vaccination. Our methodology enables us to jointly estimate the duration of PCR positivity, which differs for symptomatic and asymptomatic infection.

**Implications of all the available evidence**

The available evidence shows that booster vaccination offers modest, but tangible, increased protection against SARS-CoV-2 infection in the short-term, with prior infection conferring more robust and sustained protection. This study, and the others referenced, highlight how vaccines continue to have a role in the ongoing COVID-19 response; boosting population immunity in advance of expected periods of high prevalence. Via regular asymptomatic testing, the SIREN study offers unique insights into current infection trends, and the real-world protection conferred by booster vaccination and prior infection for a working-age population.

## Introduction

More than 3 years since SARS-CoV-2 emerged, after 770 million reported infections and 13.5 billion doses of COVID-19 vaccine delivered,^1^ most of the world's population now has some degree of immunity, whether from infection, vaccination, or a hybrid of both. How long this protection lasts, against what outcome, and how future-proof it may be to emerging variants remains uncertain. With the public health emergency declared over by the World Health Organisation,^2^ budgets, testing, and vaccination programmes have been scaled back in many countries.^3^ In the northern hemisphere, decisions about autumn vaccination campaigns are being made, with concomitant authorisation of vaccines which target emerging strains of SARS-CoV-2.^4^

In many high-income countries, following evidence of vaccine protection waning after the second dose,^5^, ^6^, ^7^ priority population groups have now been offered at least two ‘booster’ vaccinations (with up to 6 vaccines administered to the highest risk individuals).^8^ Healthcare workers without additional risk factors were offered their fourth vaccine dose in autumn 2022. This occurred during a period of dominance of Omicron sub-variants, which had higher immune escape capability from both vaccines and previous infection.^9^^,^^10^ Together with the contemporaneous removal of non-pharmaceutical interventions^11^—and the return to more normal population mixing^12^—record levels of infections were detected.^13^ Importantly, given a combination of high population immunity and variant properties, the Omicron sub-variant waves have been clinically milder than earlier variants.^14^ Considerations about future vaccination campaigns must, therefore, be made in a context of population immunity, uncertainty about variant emergence, and the requirement for sustainable post-pandemic management of COVID-19.

Healthcare workers have been prioritised for COVID-19 vaccination in the UK and many other countries, recognising their high occupational exposure, their potential role in nosocomial transmission dynamics, and the significant impact of healthcare worker absence on healthcare delivery.^15^ Whether healthcare workers should be regularly offered autumn boosters is unresolved. There are opportunities for efficient operational delivery aligned with seasonal influenza vaccination, however, consideration of perceptions and behaviours affecting vaccine uptake, and the potential risk of vaccination fatigue, is important. Efficient timing of these vaccination campaigns should also be considered, for optimal protection during periods of highest COVID-19 and other respiratory virus circulation.

The SARS-CoV-2 Immunity and Reinfection Evaluation (SIREN) study, a large-scale United Kingdom (UK) healthcare worker cohort undergoing fortnightly PCR testing and running continuously since June 2020,^16^ is uniquely placed to provide evidence to support this decision-making. Here we investigate the protection of booster vaccination and prior SARS-CoV-2 infection on the acquisition of infection (both symptomatic and asymptomatic) among our cohort of triple-vaccinated healthcare workers in a period of Omicron sub-variant circulation.^5^

## Methods

### Study design and setting

The SIREN study, run by the UK Health Security Agency (UKHSA), is a prospective cohort study of National Health Service (NHS) healthcare workers, recruiting 44,000 participants between June 2020 and March 2021. Follow-up was initially 12 months from enrolment, with subsequent extensions to 24 and 36 months. At enrolment, and at each subsequent extension, participants completed a demographic questionnaire and provided blood serum and nasal swab samples for anti-SARS-CoV-2 antibody testing and polymerase–chain reaction (PCR) testing for SARS-CoV-2 RNA. Following enrolment, participants provided samples for fortnightly PCR testing and regular (monthly or quarterly, depending on the site) antibody testing, and completed a fortnightly symptom questionnaire.^16^ The study protocol was approved by the Berkshire Research Ethics Committee on May 22, 2020. This study collected consent from all participants. ISRCTN Registry number: ISRCTN11041050.

### Participant data

We included all participants maintained under active follow-up (enrolled in the study and contributing PCR tests and/or fortnightly questionnaires) between 12th September 2022 and 31st March 2023, with more than 6 months since receipt of their third dose and contributing at least two SARS-CoV-2 PCR tests during the follow-up period. PCR tests determined a participant's status during the study analysis period. We excluded participants who had received more than three doses before September 2022. Questionnaire responses, PCR and antibody test results (including from outside the study), and information on vaccination were collected centrally by UKHSA.^16^

### Covariates

Demographic covariates were self-reported and included: age, gender identity, ethnicity, region of residence, occupational setting, staff type, medical conditions, and household structure (Table S3).

Linked testing and vaccination data included: PCR and antibody dates and results, vaccination dose, date, and manufacturer (with most receiving mRNA vaccines).

Baseline antibody tests were used to identify if participants had an early Wild-type or Alpha sub-variant infection, prior to their initial recruitment into the study. Between recruitment and the start of the study analysis period, information from both PCR testing and antibody testing were used to identify if an infection had occurred, with the date of a positive PCR test or a positive antibody test consistent with previous infection used as a proxy for the onset of infection. Participants were grouped by time since previous infection. For those without indication of prior infection we required an anti-N negative result (Roche Elecsys anti-SARS-CoV-2 nucleocapsid (anti-N) assay) within the 6 months prior to study entry to confirm their naïve status. Participants without indication of prior infection and for whom a recent serum sample (within the last 6 months) was not available for testing (n = 2100) were excluded from analyses exploring time since infection as we could not confirm their infection-naïve status.

Questionnaires completed within a 14-day window of a positive PCR test were used to distinguish between infections with and without COVID-19 symptoms. We assigned symptom statuses of: COVID-19-specific symptoms (any of: cough, fever, sore throat, anosmia, and/or dysgeusia), and asymptomatic for COVID-19 (absence of symptoms, or only non-specific symptoms such as fatigue and muscle ache).

### Representativeness

All NHS hospitals and health boards in the UK were invited to join SIREN, with no random sampling of hospitals, health boards, or participants.^16^ The cohort, whilst not representative of the general UK population, broadly reflects the demography of healthcare workers in the UK.

The Spikevax bivalent Original/Omicron and Comirnaty bivalent Original/Omicron booster vaccines received regulatory approval in August and September 2022, respectively, and were introduced for frontline healthcare workers on 12th September 2022 (with eligibility criteria consistent with third dose criteria).

### Bias

The fortnightly testing regime minimised bias in detection of SARS-CoV-2 infection, and the statistical methodology further controlled for gaps in testing. Recall bias was minimised by only considering symptoms reported within a 14-day window of a positive PCR test.

### Censoring

Testing dates were pre-determined upon study enrolment, providing interval-censored observations of infection. Participants joined the study analysis at the date of their first PCR test after 12th September 2022. Participants required at least 24 weeks since receiving a third vaccine dose to be eligible for inclusion, those with fewer than 24 weeks only entered the study once 24 weeks had elapsed. To account for study withdrawal (either because of early withdrawal or reaching the end of the follow-up period) participants were right-censored at their last recorded PCR test.

### Statistical methods

Crude PCR positivity rates were calculated as the number of detected PCR positive results per 10,000 person-days of follow-up. An exact Poisson method was used to calculate 95% confidence intervals (CI). We used multi-state models (MSMs) to estimate the hazards associated with infection for selected covariates and the time spent in the PCR positive state. MSMs describe the transition rates (“transition intensities”) between discrete states of a process (in this case between the susceptible and infected state). Specifically, we applied continuous-time MSMs and modelled the PCR data as intermittent observations of the underlying trajectory of infection and recovery. The mean time spent in a state is proportional to the inverse of the estimated transition intensity for remaining in the state. For example, if the estimated transition intensity out of a state is 0.2, the transition intensity for remaining in the state is −0.2, and the mean time spent in the state is 5 days (−1/−0.2). Duration of PCR positivity estimates were averaged over the demographic characteristics of the entire study population for comparability. We compared MSM estimates to a Cox proportional hazards model, assessed model fit by comparing expected and observed numbers in each state over time, and undertook variable selection using Akaike information criterion values and likelihood-ratio tests. Stratification and piecewise-constant hazards over time were used to account for non-proportionality. Vaccine effectiveness (VE) and relative protection estimates were obtained from hazard ratios (HR) using the formula: VE = 1—HR.^7^ The covariate levels and transitions that these covariates were included on are shown in Table S1, the list of (multi-state) models and covariates used to generate the estimates is shown in Table S2 and corresponding models are listed alongside estimates below. We used 2+ years as the baseline for time since previous infection because the confirmed naïve group was smaller and testing records indicated different behavioural trends, see Supplementary Appendix for further details.

### Model implementation

Statistical models were implemented using R v.4.3.1 (R Foundation, Vienna, Austria) and the R package msm was used to fit the multi-state models.

### Role of the funding source

The funders had no role in study design, data collection, data analysis, interpretation, or writing of the report.

## Results

### Population characteristics

**Fig. 1:**
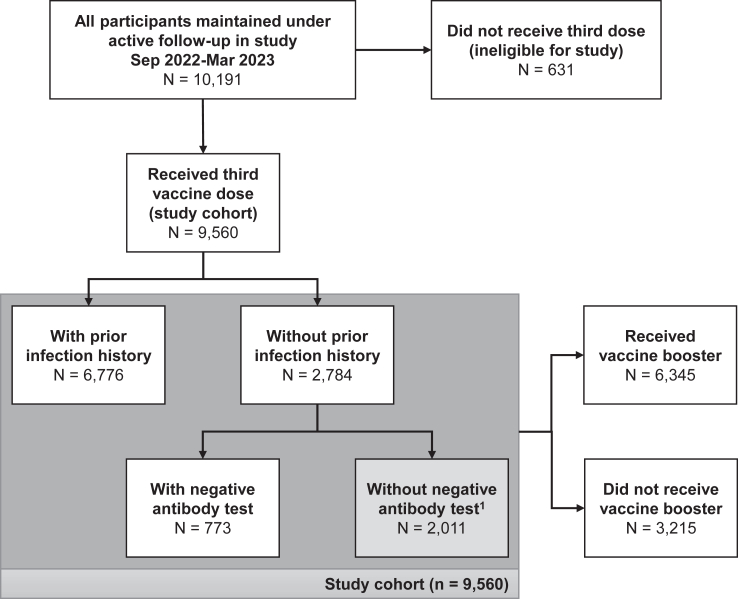
Flowchart of participation in study and uptake of booster vaccine. ^1^Individuals without prior infection history and without a negative antibody test within the 6 months prior to study entry were excluded from analyses exploring time since infection.

A total of 9560 participants were included in this analysis; 6776 (70.9%) had a previous SARS-CoV-2 infection date recorded, and 773 (8.1%) were confirmed as naïve (Fig. 1). Coverage of booster vaccination was low compared with previous vaccines; 64.3% (6143/9560) uptake within 2 months of fourth dose availability, compared to 83.0% (11,758/14,174) within 2 months of third dose availability.

The study cohort included both clinical (nurses comprised 33% (3178/9560) and doctors 12% (1155/9560) of the cohort) and non-clinical, predominantly office-based, roles (administrative/executive staff comprised 17% (1613/9560) of the cohort). Overall, 84% (7992/9560) of the study cohort were in patient-facing roles. The majority (84%; 8036/9560) reported female gender identity, with 88% (8435/9560) between the ages 35–64, and 90% (8611/9560) of white ethnicity. Most (74%; 7038/9560) had no chronic medical conditions, although 2.6% (247/9560) reported immunosuppression (Table S3).

Healthcare workers were recruited from every UK region, for this analysis the greatest proportion were resident in Scotland (1701 participants, 18%), with London (1199 participants, 13%) and the East of England (1176 participants, 12%) contributing the greatest proportions from England. Compared to the UK population, participants for whom deprivation information was available lived in less socio-economically deprived areas on average; 29% (2593/9027) in least deprived quintile, 9% (823/9027) in most deprived quintile.

All participants contributed at least one test per month of study follow-up, 88% (8400/9560) contributed at least one test per 3 weeks of study follow-up, and 53% (5109/9560) of participants contributed at least one test per fortnight of study follow-up.

### Crude PCR positivity rates

Over the study period, 1264 (13.2%) participants received at least one positive PCR result. Including re-infections, we observed 1298 distinct PCR positives over 1,521,928 person-days of follow-up, corresponding to a crude (unadjusted) PCR positivity rate of 8.53 (95% CI 8.07 to 9.01) per 10,000 days follow-up. Crude PCR positivity rates varied over the analysis period, and by region, but did not differ substantially according to other demographic characteristics (Table S4, Figure S5). Where sequencing information was available, >75% of infections were Omicron BQ.1, BA.5, and XBB (Figure S6).

### Vaccine effectiveness

**Fig. 2:**
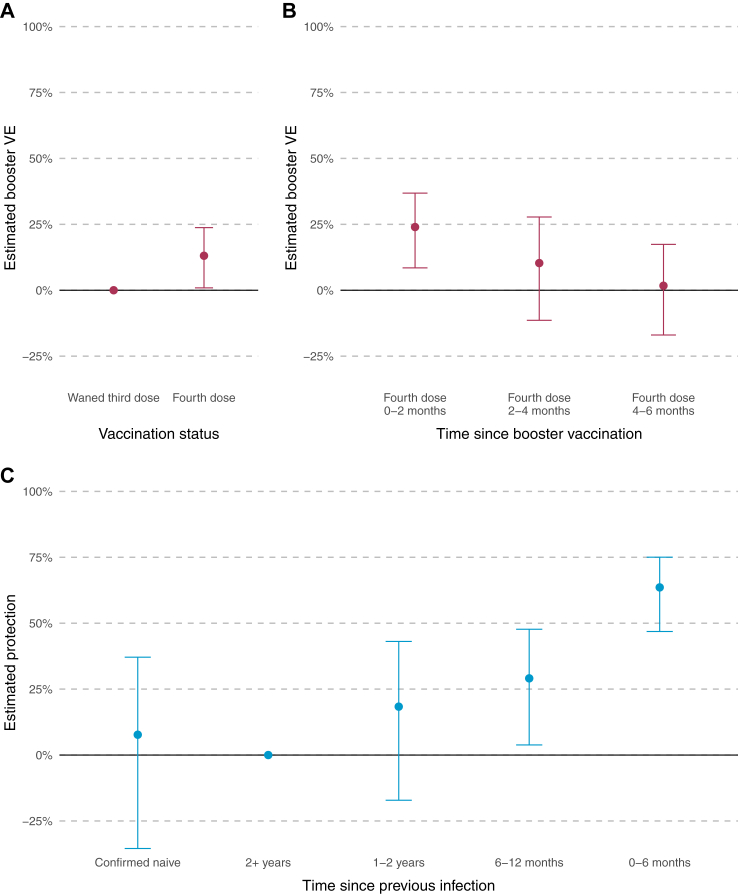
Estimated booster vaccine effectiveness (VE), relative to waned third dose, by booster vaccination status (panel A, model 1), time since booster vaccination (panel B, model 2), and estimated protection from previous infection, relative to a baseline of 2+ years (panel C, model 3). Error bars show the 95% confidence interval around the estimates.

VE of the fourth dose relative to protection at least 6 months after a third dose was estimated as 13.08% (95% CI 0.89 to 23.76) over the entire analysis period (model 1). VE was highest in the 2 months post-vaccination at 23.97% (95% CI 8.48 to 36.83), reducing to 10.30% (95% CI −11.40 to 27.78) in the period 2–4 months post-vaccination, and 1.71% (95% CI −16.97 to 17.40) in the period 4–6 months post-vaccination (Table 1, Fig. 2, model 2).

**Table 1:** Crude PCR positivity rates per 10,000 person-days and estimated vaccine effectiveness and protection from prior infection by vaccination status, time since previous infection and reported COVID symptoms.

	Number of participants	Positive PCR tests	Exposure (person-days at risk)	Crude PCR positivity rate per 10,000 person-days (95% CI)	Protection relative to baseline (95% CI)	Symptomatic		Asymptomatic	
						Positive PCR tests	Protection relative to baseline (95% CI)	Positive PCR tests	Protection relative to baseline (95% CI)
Whole population	9560	1298	1,521,928	8.53 (8.07, 9.01)	N/A	865	N/A	265	N/A
**Vaccination status**									
Waned third dose^a^	9389	541	603,023	8.97 (8.23, 9.76)	Baseline	340	Baseline	118	Baseline
Fourth dose	6345	757	918,905	8.24 (7.66, 8.85)	13.08% (0.89, 23.76)	525	8.55% (−5.75, 20.91)	147	27.99% (6.61, 44.48)
**Time since fourth dose**									
Fourth dose 0–2 months	6345	179	338,530	5.29 (4.54, 6.12)	23.97% (8.48, 36.83)	131	18.52% (0.08, 33.56)	34	39.85% (11.24, 59,23)
Fourth dose 2–4 months	5759	300	305,192	9.83 (8.75, 11.01)	10.30% (−11.40, 27.78)	212	5.80% (−18.81, 25.31)	52	26.31% (−8.34, 49.88)
Fourth dose 4–6 months	5005	278	275,183	10.1 (8.95, 11.36)	1.71% (−16.97, 17.40)	182	−1.95% (−23.36, 15.74)	61	15.56% (−18.18, 39.66)
**Time since previous infection**									
Confirmed naive	773	173	115,165	15.02 (12.87, 17.43)	7.71% (−35.45, 37.11)	124	16.06% (−29.03, 45.39)	33	−18.47% (−155.83, 45.13)
2+ years	1752	196	222,783	8.8 (7.61, 10.12)	Baseline	127	Baseline	40	Baseline
1–2 years	2850	209	195,715	10.68 (9.28, 12.23)	18.35% (−17.15, 43.09)	136	26.49% (−10.35, 51.04)	39	5.35% (−101.67, 55.58)
6–12 months	4123	347	426,815	8.13 (7.3, 9.03)	29.08% (3.82, 43.09)	226	33.85% (7.48, 52.71)	81	10.56% (−68.56, 52.55)
0–6 months	3433	83	254,866	3.26 (2.59, 4.04)	63.58% (46.85, 75.04)	38	71.99% (56.11, 82.13)	30	42.23% (−17.83, 72.65)

a Participants joined the analysis at the date of their first PCR test after 12th September 2022. 171 participants had received their fourth dose booster on or before the date of their first PCR test and contributed no follow-up time in the waned third dose vaccine status.

Compared to a waned third dose, VE was 30.87% (95% CI −84.79 to 7.31) among those confirmed as naïve, 32.87% (95% CI 8.51 to 52.20) for those with a previous infection more than 2 years ago, −1.67% (95% CI −40.95 to 26.66) for those with an infection in the past 1–2 years, 23.14% (95% CI 3.07 to 39.06) for infection in the past 6–12 months and 39.58% (95% CI 7.99 to 60.32) for infection in the past 0–6 months (Table S5, Figure S7, model 4).

### Protection from previous infection and other covariates

SARS-CoV-2 infection (symptomatic or asymptomatic) between 6 and 12 months ago was associated with a 29.08% (95% CI 3.82 to 43.09) increase in protection compared to individuals who had an infection more than 2 years ago; an infection within the last 6 months was associated with a 63.58% (95% CI 46.85 to 75.04) increase in protection; and those with an infection 1–2 years ago or never infected were not associated with significantly more or less protection, 18.35% (95% CI −17.15 to 43.09) and 7.71% (95% CI −35.45 to 37.11), respectively (Table 1, Fig. 2, model 3).

### Symptomatic vs asymptomatic infection

Among the 1298 positive PCR results, 1130 had symptom information reported. Of these, 865 (76.5%) had COVID-19 symptoms, and 265 (23.5%) were asymptomatic (132 had symptoms unrelated to COVID-19, and 133 had no symptoms). The proportion symptomatic was slightly higher among those with a booster dose (78.1%, 525/672) compared to those with a waned third dose (74.2%, 340/458). Among those with an infection in the past 6 months, 55.9% (38/68) reported symptoms compared to >70% of those confirmed naïve or with an infection more than 6 months prior (Table 1, Figure S8).

**Fig. 3:**
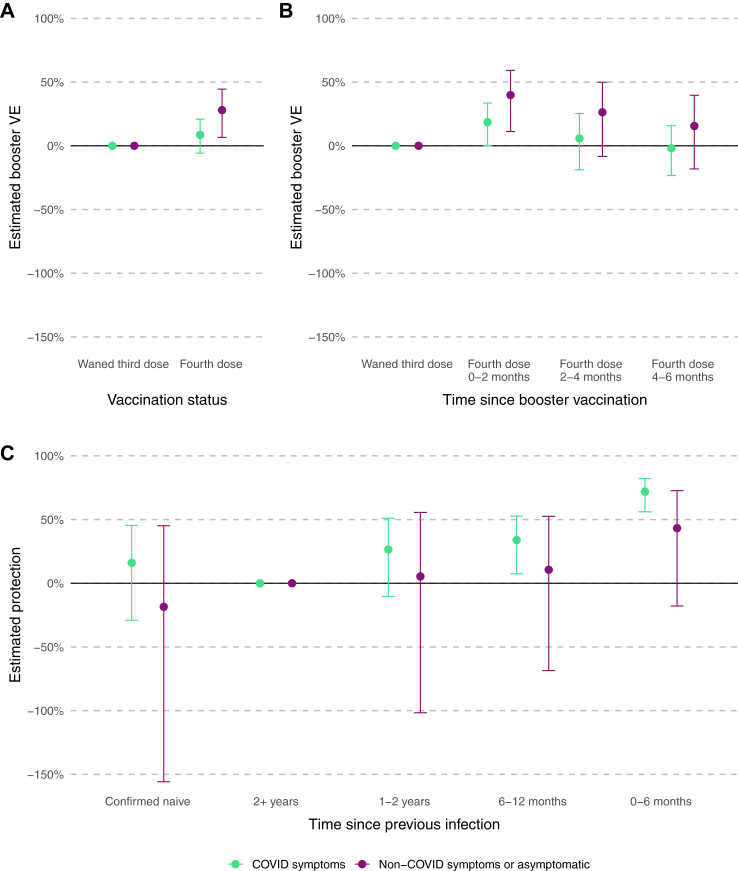
Estimated booster vaccine effectiveness (VE), relative to waned third dose by symptom status and booster vaccination status (panel A, model 5), time since booster vaccination (panel B, model 6), and estimated protection from previous infection, relative to a baseline of 2+ years (panel C, model 7). Error bars show the 95% confidence interval around the estimates.

Relative to a waned third dose, VE was 8.55% (95% CI −5.75 to 20.91) against symptomatic infection and 27.99% (95% CI 6.61 to 44.48) against asymptomatic infection. Relative to an infection more than 2 years previously, an infection in the past 0–6 months was associated with 71.99% (95% CI 56.11 to 82.13) increased protection against symptomatic infection and 42.23% (95% CI −17.83 to 72.65) against asymptomatic infection (Table 1, Fig. 3, models 5–7).

### Duration of PCR positivity

Duration of PCR positivity was estimated as 7.51 days (95% CI 6.94 to 8.13) overall. When averaged over the study population, this duration was shorter among those with a booster vaccination at 6.90 days (95% CI 5.87 to 8.11), compared to 8.50 days (95% CI 6.79 to 10.64) for those with a waned third dose (Table S6, model 1).

**Fig. 4:**
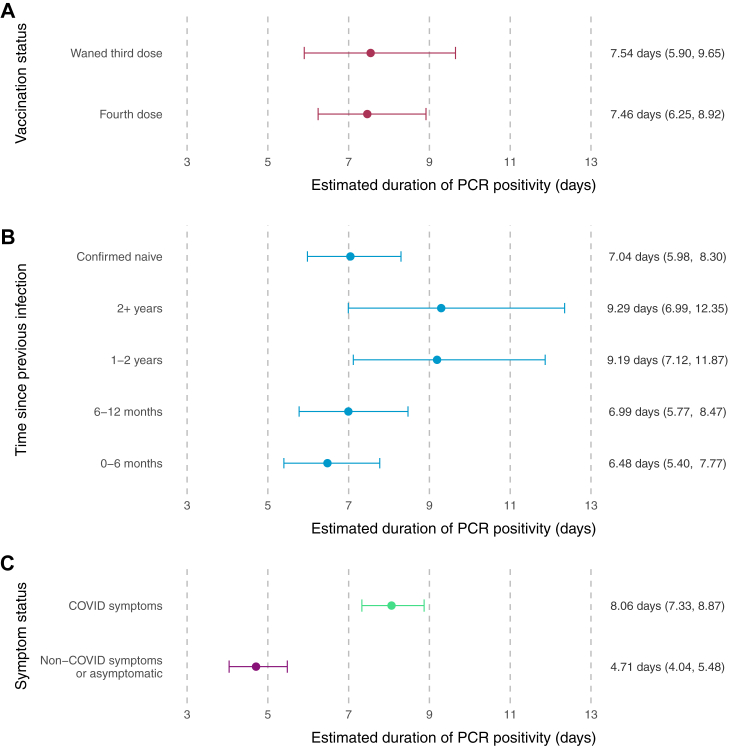
Estimated duration of PCR positivity (days), averaged across the study population, by vaccination status (panel A, model 1), time since previous infection (panel B, model 2), and symptom status (panel C, model 5). Error bars show the 95% confidence interval around the estimated duration of PCR positivity. Estimated time and 95% confidence interval are shown alongside.

The estimated PCR positive duration was 9.51 days (95% CI 7.08 to 12.78) for confirmed naïve participants and 7.25 days (95% CI 6.32 to 8.31) for those with an infection within the past 0–6 months. Infections reported as symptomatic had a longer duration at 8.09 days (95% CI 7.36 to 8.90), compared to 4.70 days (95% CI 4.04 to 5.47) for asymptomatic (Fig. 4, model 3).

## Discussion

We have estimated real-world effectiveness of second COVID-19 booster vaccines, protection against symptomatic and asymptomatic SARS-CoV-2 following previous infection, and the duration of PCR positivity in our cohort of UK healthcare workers over autumn/winter 2022–23, a period with high circulation of Omicron sub-variants. In this cohort of triple-vaccinated, generally healthy, working-age adults we found booster vaccines provided modest and short-lived additional protection against infection. A recent previous infection provided more sustained protection but waning over time was still evident. Asymptomatic infection was more common amongst those with a recent previous infection, and these infections had shorter duration of positivity.

Our study adds to the growing literature on booster VE in the context of high population immunity and high infection rates. A recent cohort study in the Netherlands estimated fourth booster VE of 14% (95% CI 1 to 25%), albeit using self-reported infection data for an older cohort.^17^ Meanwhile, early fourth booster VE estimates from a study of United States (US) pharmacies were 49% (95% CI 41 to 55%) and 40% (95% CI 28 to 50%) for age groups 18–49 and 50–64, respectively.^18^ This analysis did not control for prior infection history, and biases in the study design mean this may be an over-estimate.^19^ In both studies, only data on symptomatic infection was able to be collected, whereas a key strength of SIREN is the capability to detect both symptomatic and asymptomatic infection, and to link to an individuals’ complete testing history.

As seen following third dose vaccination in our cohort^7^ and elsewhere,^20^ protection from infection and protection by vaccination and infection (“hybrid immunity”) conferred longer-lasting immunity than vaccination alone. This may stem from the differential cellular immune responses to infection vs. vaccination,^21^ additionally, mucosal immunity appears to be conferred by infection rather than vaccination.^22^^,^^23^

For the Omicron BA.4/5 era in particular, greater and more durable protection was reported for infection-experienced individuals as compared to naïve individuals in results from the nationally-representative UK COVID-19 Infection Survey (CIS),^24^ with rapid waning of mRNA vaccine protection from 2 months post-vaccination. The CIS study estimated >80% protection against Omicron BA.4/5 reinfection for those with a previous Omicron BA.2 infection.^24^ This is higher than our estimated 64% protection from a recent (Omicron-period) infection and may reflect our younger cohort and greater number of prior infections among healthcare workers.

A quarter of infections detected in our study were reported as asymptomatic, similar to other European countries during Omicron BA.1 dominance.^25^ We found a fourth dose was associated with greater protection against asymptomatic than symptomatic infection, whilst prior infection provided more protection against symptomatic infection, particularly if an infection had occurred recently (i.e. during the Omicron-circulating period). Given most comparably scaled studies have used symptomatic infection as their outcome we are unable to directly compare this result.

We estimate important distinctions in duration of PCR positivity between sub-groups. Several studies have investigated duration of positivity for Omicron-era infections^26^ and, whilst most estimate around 7 days, consistent with our findings, these studies may suffer from small sample sizes or employ methodology that over-estimates duration. In comparison, our analysis correctly accounts for interval-censoring and uncovers important distinctions between sub-groups that would be missed by an empirical approach (e.g. median time between initial PCR positive to subsequent PCR negative). We did not collect information on infectiousness (evidence for which remains varied^26^) but it was notable that individuals with asymptomatic infection, and, to a more limited extent, those either vaccinated or with recent previous infection, were estimated to have shorter durations of PCR-positivity. These estimates can help to inform infection-control measures and transmission models to forecast prevalence.

### Limitations

The SIREN cohort is a cohort of working-age healthcare staff, with participants being predominantly female, of white ethnicity, healthy, and middle-aged. We have controlled for many of these factors in our analysis of VE, however the relatively small proportions of males, older participants, and those with high multimorbidity limits full generalisability to the wider UK population.

Vaccination was not randomly assigned, and despite limited differences in vaccine uptake by measured demographic, we could not control for several other prognostic factors which may be associated with vaccination, e.g. an individual's perceived exposure risk, which may alter their decision to receive a booster vaccination.

We did not investigate severe disease, which is rare in this cohort. Other studies have found VE against severe disease in the Omicron era to be higher and longer-lasting than against mild disease.^27^^,^^28^

Given the very small number of unvaccinated individuals in SIREN and recognising they may have different risk profiles to vaccinated individuals, we were unable to use them as a reference group to estimate absolute VE. Previous studies of this cohort have demonstrated a dramatic reduction in infection risk for vaccinated as compared to unvaccinated individuals.^29^

We did not compare vaccination type as most of our cohort received the same schedule before study entry (93% three mRNA doses). The COV-BOOST trial found more durable immune response with heterologous third doses.^30^ Therefore, potentially the 6% of participants with two viral vector vaccine doses followed by an mRNA vaccine may have had slightly more durable protection, although the effect is expected to be minimal across such a large cohort.

Due to the small number of truly asymptomatic cases, for our symptoms analysis we grouped together asymptomatic cases and those reporting only non-COVID-19-specific symptoms, such as fatigue or muscle ache. Whilst non-specific symptoms occurring around the time of COVID-19 infection may be linked to the infection, this grouping reflects the fact that most participants reported one or more of these non-specific symptoms at some point during the study period, regardless of PCR status.

### Conclusions

In this highly vaccinated, infection-experienced, working-age cohort there was a small but short-lived increase in protection against SARS-CoV-2 infection associated with receipt of booster vaccination during an Omicron sub-variant period. Given the more marginal benefit in comparison with first boosters, and the notably lower coverage of second boosters, economic evaluation will be increasingly important in informing future vaccine deployment. With SARS-CoV-2 yet to settle into a seasonal pattern and considering the short-lived protection provided by current COVID-19 vaccines, it appears premature to plan mass roll-out of annual boosters akin to Influenza. Currently, therefore, vaccine boosters remain an important tool in responding to the dynamic COVID-19 landscape; boosting population immunity in advance of periods of anticipated pressure, such as surging infection rates or emerging variants of concern.

## Contributors

SH conceived and supervised the study. PDK, VJH, SF and SH designed the analysis plan, did the literature search, and drafted the manuscript. VJH and SF cleaned and finalised the dataset for analysis and did the descriptive analyses. PDK, AC, AP and DDA planned and did the statistical analysis, with support from CHJ. VJH, SF, AC and AA verified the data in the study. ADO, KM, DS, AH, NP, JB, DCr, CN, DCo, CHJ, MC, CSB, AA, JI, AMP, AC and DDA revised the manuscript for important intellectual content and approved the manuscript for publication. All authors had full access to all the data in the study and accept responsibility to submit for publication. The lead author affirms that this manuscript is an honest, accurate, and transparent account of the study being reported; that no important aspects of the study have been omitted; and that any discrepancies from the study as planned (and, if relevant, registered) have been explained.

## Data sharing statement

The metadata is available through the Health Data Research UK Co-Connect platform. Anonymised data will be made available for secondary analysis to trusted researchers upon reasonable request.

A list of all R packages used and R code written to process the data, implement the statistical analysis, and produce the figures and tables is available online at https://github.com/SIREN-study/SARS-CoV-2-booster-vaccination and may be cited with Digital Object Identifier: 10.5281/zenodo.10055166. UKHSA and the MRC Biostatistics Unit have public facing websites and Twitter accounts @UKHSA and @MRC_BSU. UKHSA and the MRC Biostatistics Unit engage with print and internet press, television, radio, news, and documentary programme makers.

## Declaration of interests

We declare no competing interests. DCr reports participation on steering groups for the SIREN Scotland trial and TAVI UK trial and is a trustee of the Medical Schools Council of the UK. DDA reports attendance and travel expenses for MRC board/panel meetings and is a member of the Scientific Pandemic Influenza Advisory Committee, the steering group for SIREN Scotland; the MRC Infection Immunity board, the MRC Population Health Science Group, the MRC Better Methods for Better Research Panel, and the Joint Biosecurity Centre/UKHSA Data Science Advisory Board. CSB and SH report grant funding from the NIHR HPRU. CSB reports participation in an ad-hoc one-off market research advisory on a variety of infection topics.

## Appendix A Supplementary data

Supplementary Appendix

SIREN Protocol

SIREN Statistical Analysis Plan

SIREN Study Group
