# Supplementary material for: Effect of second booster vaccinations and prior infection against SARS-CoV-2 in the UK SIREN healthcare worker cohort
Source: Lancet Reg Health Eur. 2023 Dec 14;36:100809. doi: 10.1016/j.lanepe.2023.100809 (PMC10727938; doi:10.1016/j.lanepe.2023.100809)
Supplement: Supplementary Appendix [file mmc1.pdf]

Supplementary Appendix for: Protection of COVID-19 booster vaccination and previous infection against symptomatic and asymptomatic SARS-CoV-2

Contents

1 Supplementary methods **2**

1.1 Multi-state models . . . 2

1.2 Cox proportional hazards models . . . 5

1.3 Methodological discussion . . . 6

1.4 Model assumptions . . . 7

2 Supplementary information **9**

2.1 Participant and public involvement . . . 9

2.2 Dissemination to participants and related patient and public communities . . . 9

3 Supplementary figures **10**

4 Supplementary tables **19**

References **27**

1 Supplementary methods

1.1 Multi-state models

Overview

Multi-state models can be used to describe health-related processes over time, particularly those relating to disease epidemics. In multi-state models, an individual begins in a given state, spends a period of time in that state, then moves to a next state. Examples include survival models, where individuals move from the alive state to the dead state, and disease-staged models, where each state represents disease severity, with movement from state to state representing disease progression [1]. The period of time spent in a state and the state to which the individual moves can be thought of as random variables which may be estimated.

Intermittently observed data

In cohort studies, data will often be intermittently observed, reflecting observations of an individual's status at a given time point (which may be pre-specified). An example of intermittent observation of an underlying process is shown in the below diagram.

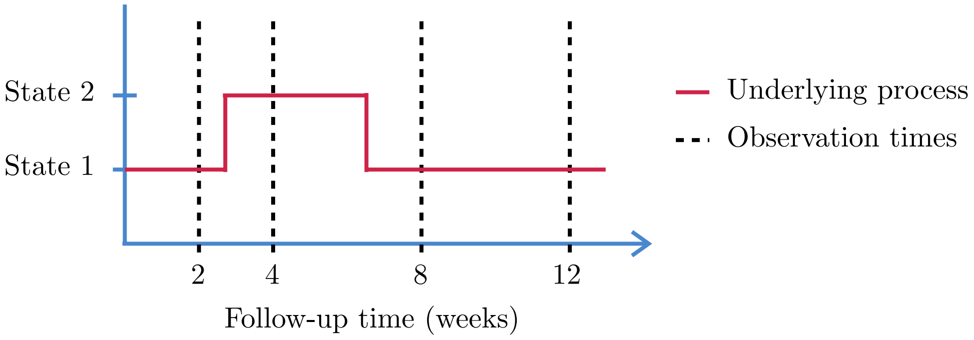

Despite the underlying process being intermittently observed, features of the underlying process may be estimated by fitting a multi-state model to the set of observed state transitions for a group of individuals. Estimated quantities include the mean time spent in a state and probability of moving between states, as well as associated hazards of transition for a set of given covariates. Further details of multi-state models are provided in the subsequent sections, and in the literature [1, 2, 3].

Generic multi-state model

Multi-state models are governed by a set of transition intensities $q_{rs}(t, z(t))$ for each pair of states r and s. The intensities may also depend on the time of the process t, or more generally a set of individual-specific and/or time-varying explanatory variables $z(t)$.

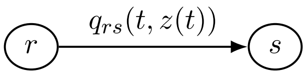

The transition intensity represents the instantaneous risk of moving from state r to state s and is defined by the limit as δt tends to zero:

$$q_{rs}(t, z(t)) = \lim_{\delta t \to 0} \frac{P(S(t+\delta t) = s | S(t) = r)}{\delta t}$$

The intensities form a transition intensity matrix Q whose rows sum to zero, with diagonal entries defined by:

$$q_{rr}(t, z(t)) = -\sum_{s \neq r} q_{rs}(t, z(t))$$

Typically multi-state models will be Markov, i.e. with the assumption that future evolution only depends on the current state and time i.e. $q_{rs}(t, z(t), \mathcal{F}_t) = q_{rs}(t, z(t))$, where $\mathcal{F}_t$ is the observation history of the process up to time t. For conciseness, hereon we denote the implicitly time-varying transition intensity simply as q_{rs}.

Let Q be the $r \times s$ matrix formed by the q_{rs}, then the transition probability matrix, with probability of moving between states in time t, $P(t)$ is:

$$P(t) = \exp(tQ) = \sum_{n=0}^{\infty} \frac{t^n}{n!} Q^n$$

and the sojourn time (mean time spent in a state) is:

$$E(T_r) = -\frac{1}{q_{rr}}$$

Covariates

Covariates can be included in multi-state models through a proportional hazards model, whereby the transition intensities q_{rs} are replaced by $q_{rs}(\boldsymbol{z_i})$ for a given set of m constant or time-varying covariates z_{im}:

$$q_{rs}(z_i) = q_{rs}^{(0)} \exp\left(\sum_{m=1}^{M} \beta_m z_{im}\right)$$

where $q_{rs}^{(0)}$ are the baseline hazards and $\exp(\beta_m)$ is the hazard ratio for the mth covariate.

Censoring

Multi-state models are well-suited to investigating interval-censored 'panel' data, where observations represent PCR testing rather than the occurrence of infection (i.e. where sampling times are non-informative). The time intervals of observation for the SIREN study are fixed in advance (2-weekly intervals). A continuous-time model is appropriate in the context of these data, allowing for changes of state (from susceptible to infected, and infected to recovered) to occur at any point during the 2 week interval. This multi-state framework additionally allows incomplete testing (i.e. gaps in an individual's testing history) to be implicitly accounted for, without the assumption that an individual remains uninfected despite not testing.

For an individual with a PCR negative status at time L and PCR positive status at time R, infection is assumed to occur at some time $T \in [0, \infty)$ during the time interval $(L, R]$.

The infection time is interval-censored, $T \in (L, R]$, and, if the time intervals are fixed in advance, this interval censoring is independent of T [2]:

$$P(L < T \leq R | L = l, R = r) = P(l < T < r)$$

Likelihood

For interval-censored panel data we have information on states $(x_{i1}, \ldots, x_{in_i})$ at times $(t_{i1}, \ldots, t_{in_i})$ for person i which may take the values PCR negative (Susceptible) or PCR positive (Infected). As described by Kalbfleisch & Lawless [4], the likelihood contribution to the transition intensities q_{rs} from person i is:

$$L_i(q_{rs} \mid x_i) = p(x_{i1} \mid x_{i0}) p(x_{i2} \mid x_{i1}) \ldots p(x_{in_i} \mid x_{in_i - 1})$$
$$\text{where: } p(x_{ij} \mid x_{ij-1}) = p_{x_{i,j-1}, x_{i,j}}(t_{i,j-1}, t_{i,j} \mid q_{rs})$$

The full likelihood $L(Q)$ is the product of the $L_{i,j}$ over all individuals and transitions. An estimate of the transition intensity matrix Q is obtained by maximising this likelihood.

Piecewise-constant hazards

Risk of community transmission of COVID-19, as well as risk of within-hospital transmission for healthcare workers, has varied throughout the study. The calendar time at which a SARS-CoV-2 infection takes place must therefore be considered. A time-varying covariate $z(t)$ for calendar month allowing for piecewise-constant hazards for each month are used to model the changing risk of infection over the study period.

Model specification and implementation

Two multi-state models were used for this study:

The first was a two-state model where participants were grouped into susceptible (with a negative PCR test) or infected (with a positive PCR test).

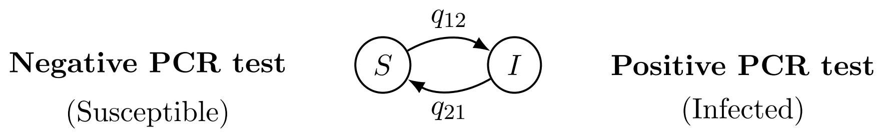

The second model included symptom status information as an outcome, sub-dividing the infected state into asymptomatic or symptomatic infection.

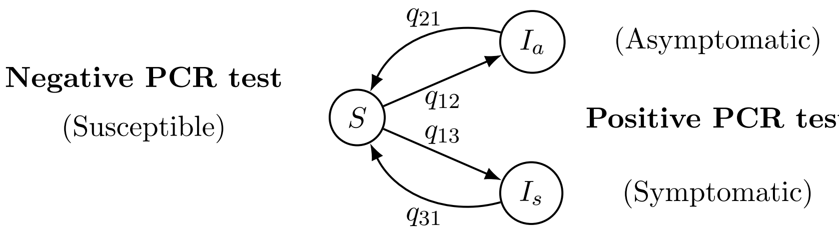

The models were specified to include proportional hazards for selected covariates on each transition and piecewise-constant hazards for the month covariate to capture the changing risk of SARS-CoV-2 infection (Table S1).

The R package msm [3] was used to implement maximum-likelihood estimation for these multi-state models.

1.2 Cox proportional hazards models

Overview

The Cox proportional hazards models is another common approach used to fit regression models with survival outcomes.

Following the notation used previously we define a hazard (which may be time-dependent) at time t as $q(t)$, with a baseline hazard $q^{(0)}(t)$, then for a given set of m constant or time-varying covariates z_{im}:

$$q(t|z_i) = q^{(0)}(t) \exp\left(\sum_{m=1}^{M} \beta_m z_{im}\right)$$

where $\exp(\beta_m)$ is the hazard ratio for the mth covariate.

Stratified Cox proportional hazards model

A key assumption of Cox models is that hazards are proportional. For covariates which exhibit non-proportionality, covariate stratification may be used.

For the example of vaccination and age, an unstratified model would state:

$$q(t|z) = q^{(0)}(t) \exp(\beta_{\text{age}} z_{\text{age}} + \beta_{\text{vax}} z_{\text{vax}})$$

This implies that the trend in survival over time is the same for all age groups, and that (hazard for age group 2)/(hazard for age group 1) is a constant. Stratification avoids needing to make this assumption by allowing the baseline hazard to differ by different covariate levels, fitting two (or more) separate equations with a common regression coefficient β_{vax}:

$$q_{\text{age}=1}(z) = q^{(0)}_{\text{age}=1} \exp(\beta_{\text{vax}} z_{\text{vax}})$$
$$q_{\text{age}=2}(z) = q^{(0)}_{\text{age}=2} \exp(\beta_{\text{vax}} z_{\text{vax}})$$

Comparison of estimated hazards

Two MSMs were used for the main analysis: the two-state model where participants moved between 'susceptible' (PCR-negative) and 'infected' (PCR-positive) states, and the three-state model which incorporated symptom status in the 'infected' states. For comparison purposes, a stratified Cox proportional hazards model was fitted to the same dataset. The estimates of this simpler model (which didn't consider recovery or interval censoring) were

able to be compared to the more complex models, as well as providing a historical comparison to previous SIREN analyses which have used stratified Cox models only.

We used three models to generate our estimates: the Cox model (equivalent to a two-state MSM without recovery or interval censoring), the two-state MSM with recovery, and the three-state MSM with recovery. Each of the three models was run with four scenarios:

1. Not including time since previous infection as a covariate and using a binary indicator of vaccination, to estimate overall vaccination effectiveness in a cohort with heterogeneous time since previous infection.

2. Not including time since previous infection as a covariate and using a time-varying indicator of vaccination, to estimate vaccine effectiveness by time since vaccination in a cohort with heterogeneous time since previous infection.

3. Including time since previous infection and a binary indicator of vaccination, to estimate protection associated with prior infection.

4. Including time since previous infection and an interaction effect between this variable and vaccination, to estimate the marginal effectiveness of vaccination by time since prior infection.

The list of models is shown in Table S2, and Figures S1-S4 compare the estimates from the MSM and Cox proportional hazards models.

For the main analysis we converted the hazard ratios (HR) estimated by the MSMs into vaccine effectiveness (VE) and relative protection estimates using the formula: VE = 1 − HR. Duration of positivity estimates were averaged over the study population to provide comparable estimates.

The R package survival [5] was used to implement estimation for the stratified Cox proportional hazards models. The Cox proportional hazards models included the covariates specified previously, with stratification on the occupational setting, age group, and region covariates. As with previous analyses of this cohort, we allowed for deferred study entry and re-entry, with participants entering or re-entering the cohort 6 weeks after the date of a positive PCR test. The Cox models were fitted both with and without a random effect on the hospital trust (i.e. a frailty model) to control for trust-specific effects, with very minimal effect on estimates [6].

1.3 Methodological discussion

Use of multi-state models

We estimated very similar hazard ratios from the MSMs and comparable Cox proportional hazards models. The slightly wider confidence intervals from the MSMs may be a more accurate reflection of the uncertainty in the estimates, given that they account for the interval censoring/missed testing.

Using MSMs we could account for gaps due to missed testing and jointly estimate the time spent in a positive state, as well as symptomatic vs asymptomatic infection. Such methods

are growing in popularity, with tutorials directed towards epidemiological research [7, 8, 9], and are well-suited to studies involving interval-censored panel-style data, although we note the MSMs took significantly longer to fit in our study (several hours compared to under a minute for the Cox proportional hazards model) and required careful tuning of the optimizer to converge to the maximum likelihood.

Stratification and piecewise-constant hazards over time were used to account for non-proportionality, however, it is possible that the proportional hazards assumption may still be unrealistic for the covariates we have studied, a common issue particularly in medical studies. Stensrud and Hernán recommend that hazard ratios should be interpreted as a weighted average of the true hazard ratios over the entire follow-up period [10].

Choice of baseline

For this study we elected to use '2+ years' as the baseline group for the covariate time since previous infection. Whilst a comparison to the immunological baseline (confirmed naïve) would be desirable, in our study this is a very small group and using this baseline introduces greater uncertainty to the estimates. Furthermore, the infection trends for the confirmed naïve group indicate likely differences in risk behaviour, e.g. more shielding, making them less representative of the larger cohort.

Whilst the pandemic has evolved, the SIREN study continues to provide relevant and generalisable results. Most of our study population have now experienced a prior COVID-19 infection, therefore selecting a group with a previous infection as a baseline seems appropriate. We chose '2+ years' due to size of this group, and based on previous analysis [11] it was reasonable to assume this group was the least protected. Our analyses test whether a more recent infection was indicative of better protection.

For completeness, we have re-run the estimates with the confirmed naïve group as a baseline, and include these in Figures S10 and S11.

Causal interpretation of VE

Work to investigate VE under different counterfactual scenarios is underway to more fully assess the causal effect of vaccination on reducing acquisition of SARS-CoV-2 infection in this population [12].

1.4 Model assumptions

In fitting the multi-state model we have made several assumptions:

Markov assumption

The Markov assumption states that future trajectories depend only on the current state and time. This assumption is unlikely to hold true immediately post-infection and immediately post-recovery, when state-transitions are less likely. We investigated two alternate model specifications to assess the sensitivity of our estimates to this assumption:

1. A semi-Markov model, allowing for a mix of long-stayers and short-stayers in the infected state. To implement this model we split the I state into two 'hidden' states I_1 and I_2, as shown in the below diagram. For more information on semi-Markov models see [13].

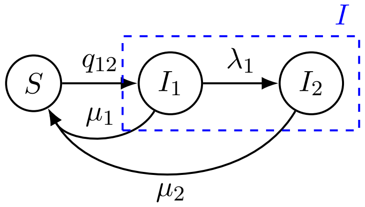

The proportion of long-stayers and short-stayers estimated by the semi-Markov models were found to be sensitive to initial values, although without a significant difference in the estimated hazards or overall duration of PCR positivity estimates to the two-state (SI) model.

2. A model including a convalescent or recovery state, in which participants cannot be re-infected. This is implemented by the addition of the R state, as shown in the below diagram, and defining a length of time during which individuals should remain in the R state (e.g. 6 weeks post-infection).

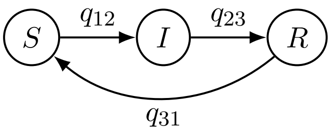

Models which included a convalescent state gave very similar estimated hazards and duration of PCR positivity estimates to the two-state (SI) model.

The two-state (SI) model defined previously had lower AIC than either of the alternate model specifications shown here, indicating a more parsimonious model. Neither of these alternate models were used for the final estimates.

No state misclassification

We assume no state misclassification in our model. Whilst the Coronavirus Infection Survey (CIS) [14] estimates PCR test sensitivity (true positive rate) to be between 85-98%, and specificity (true negative rate) to be 99.9%, the SIREN study includes serial PCR testing and linked antibody test information which enabled a manual review of positive PCR tests. Any spurious results were identified and reviewed by two researchers, and remaining misclassification is likely to be small.

Non-informative sampling

SIREN study participants were assigned to a fortnightly PCR testing pattern, and the majority tested according to this fortnightly schedule. We use the multi-state model to implicitly account for any gaps in testing, as described above. It is possible that some symptomatic testing will have taken place amongst our cohort, which would increase the probability of detecting infection, although the extent of out-of-schedule PCR testing in the study was observed to be small.

2 Supplementary information

2.1 Participant and public involvement

The SIREN study has an active Participant Involvement Panel (PIP), co-delivered with the British Society for Immunology. The SIREN PIP meets regularly to consult participants on proposed study developments, recent outputs and obtain feedback on study delivery. We have a multi-method cohort retention programme informed by the PIP and wider participant feedback which we collect continuously and through cross-sectional surveys.

2.2 Dissemination to participants and related patient and public communities

We use diverse channels to disseminate our findings to participants, including via regular digital newsletters, live webinars, videos, blogs and through our study webpage on the UKHSA website (https://www.gov.uk/guidance/siren-study).

3 Supplementary figures

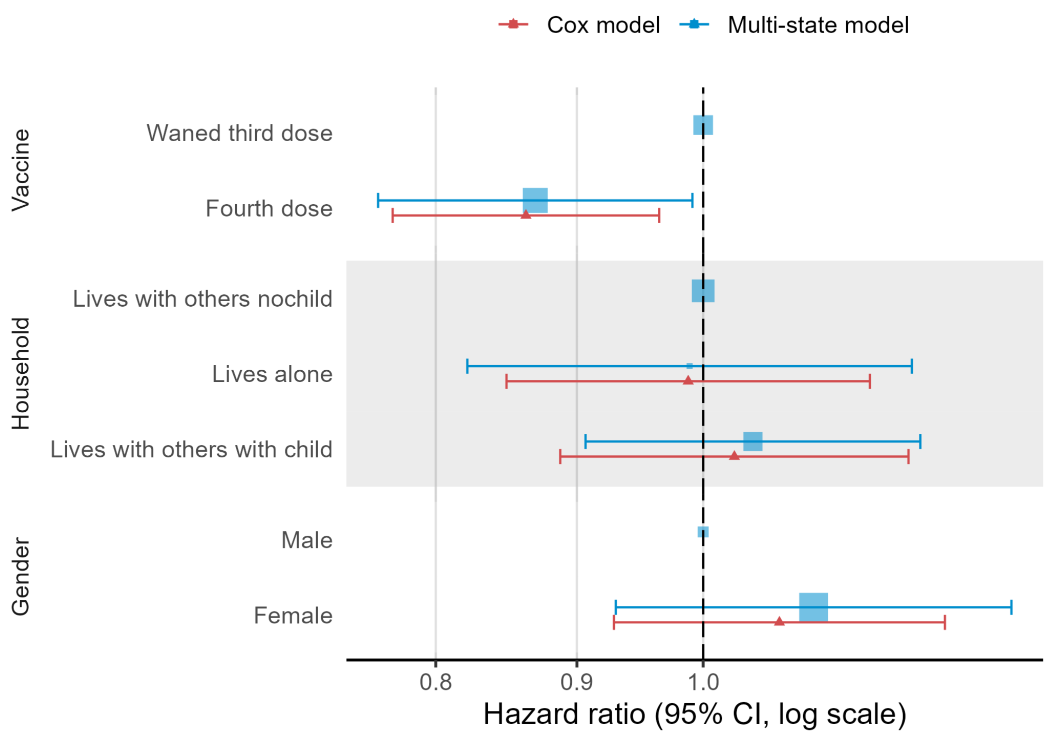

Figure S1: Comparison of hazards estimated by MSM model 1 and Cox model 1, for covariates common between the two models. Error bars show the 95% confidence interval around the estimated hazards, the size of the marker indicates the relative size of each subgroup.

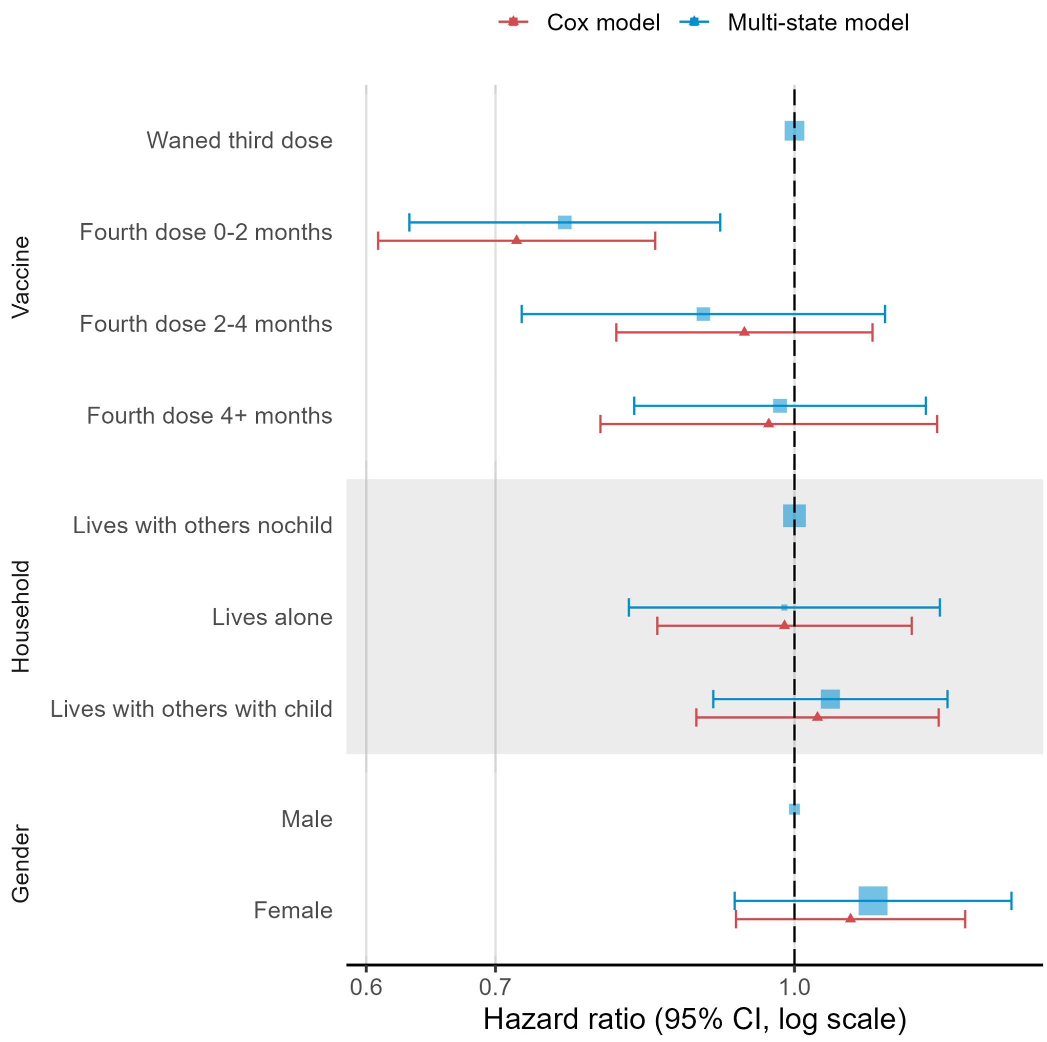

Figure S2: Comparison of hazards estimated by MSM model 2 and Cox model 2, for covariates common between the two models. Error bars show the 95% confidence interval around the estimated hazards, the size of the marker indicates the relative size of each subgroup.

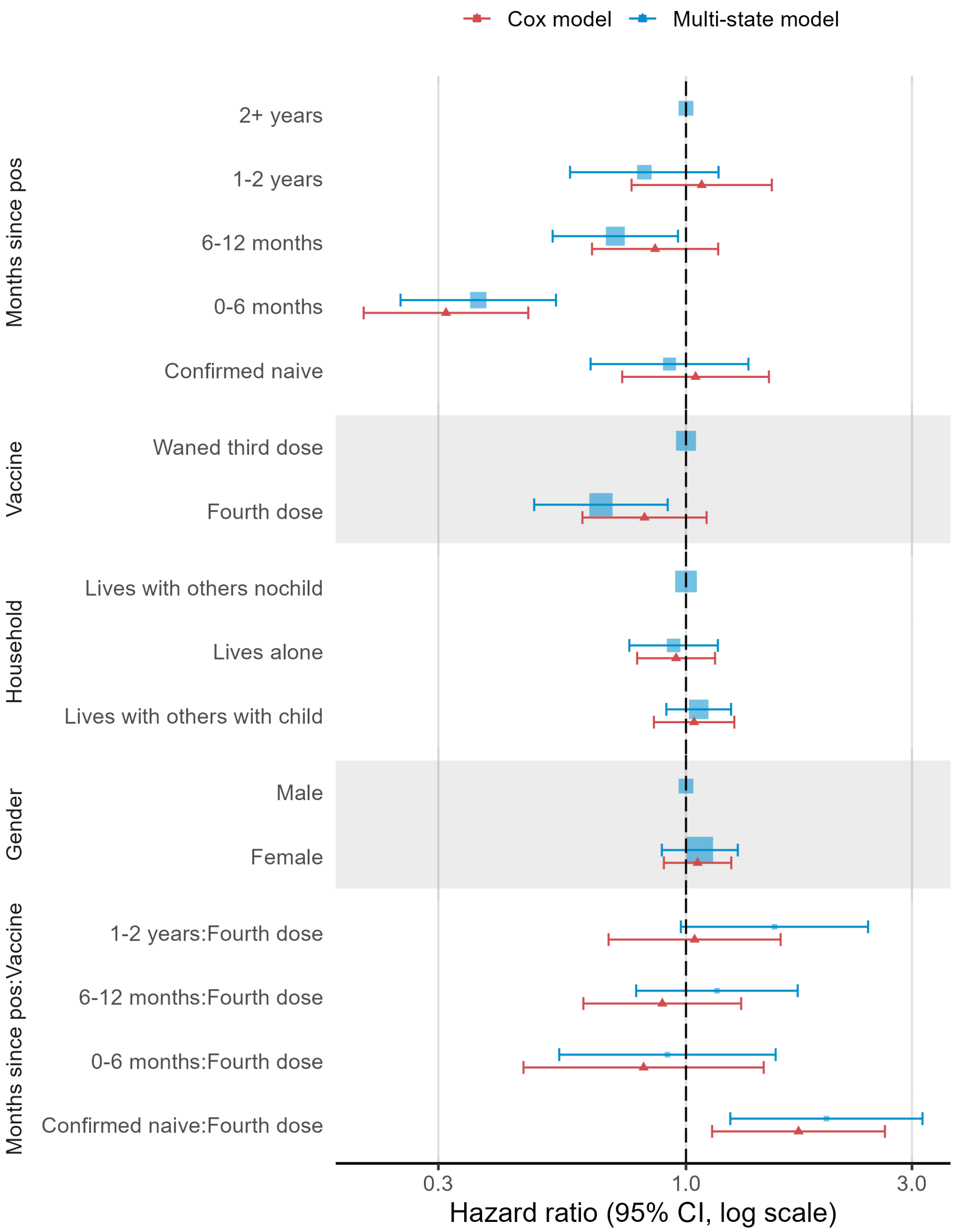

Figure S3: Comparison of hazards estimated by MSM model 3 and Cox model 3, for covariates common between the two models. Error bars show the 95% confidence interval around the estimated hazards, the size of the marker indicates the relative size of each subgroup.

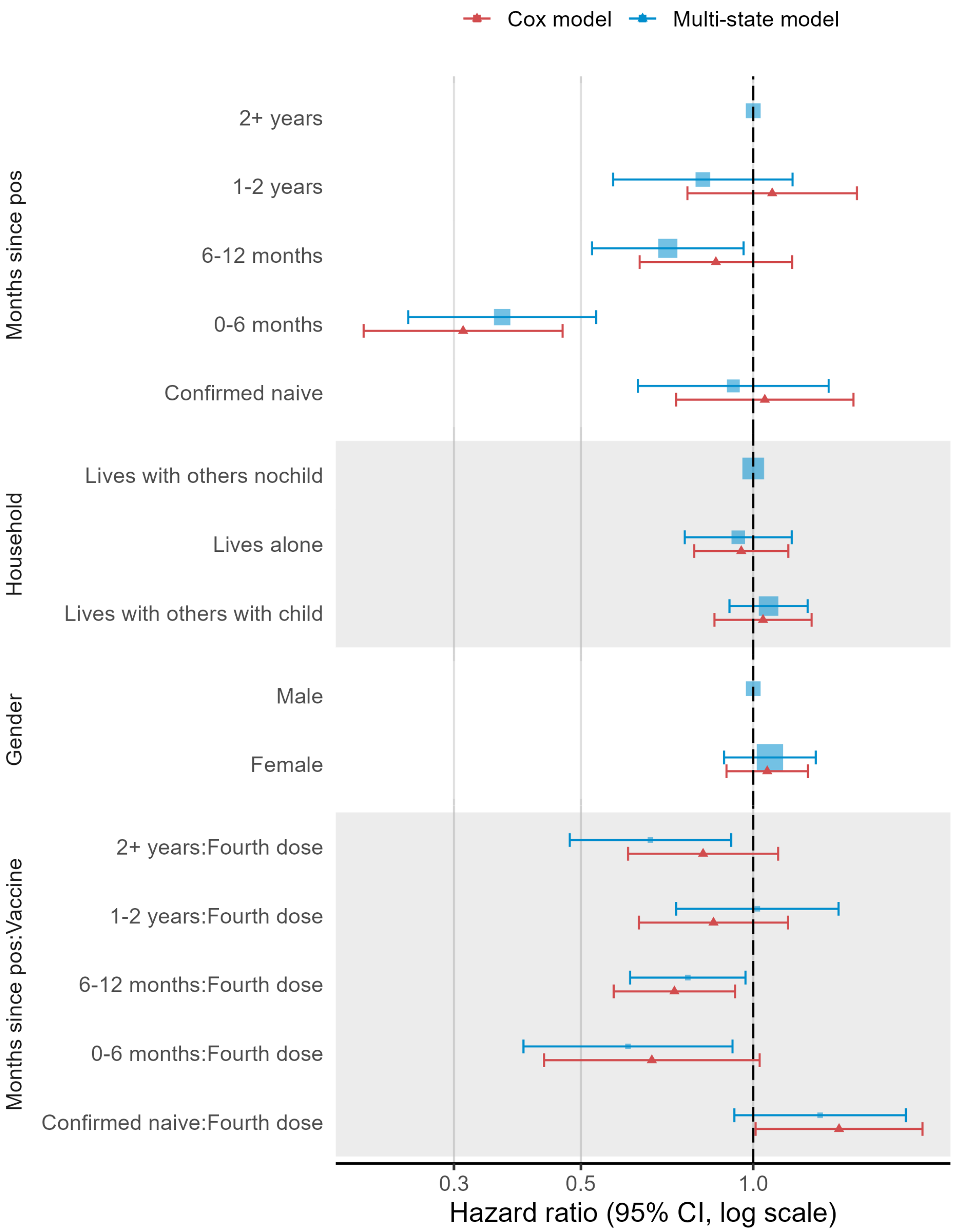

Figure S4: Comparison of hazards estimated by MSM model 4 and Cox model 4, for covariates common between the two models. Error bars show the 95% confidence interval around the estimated hazards, the size of the marker indicates the relative size of each subgroup.

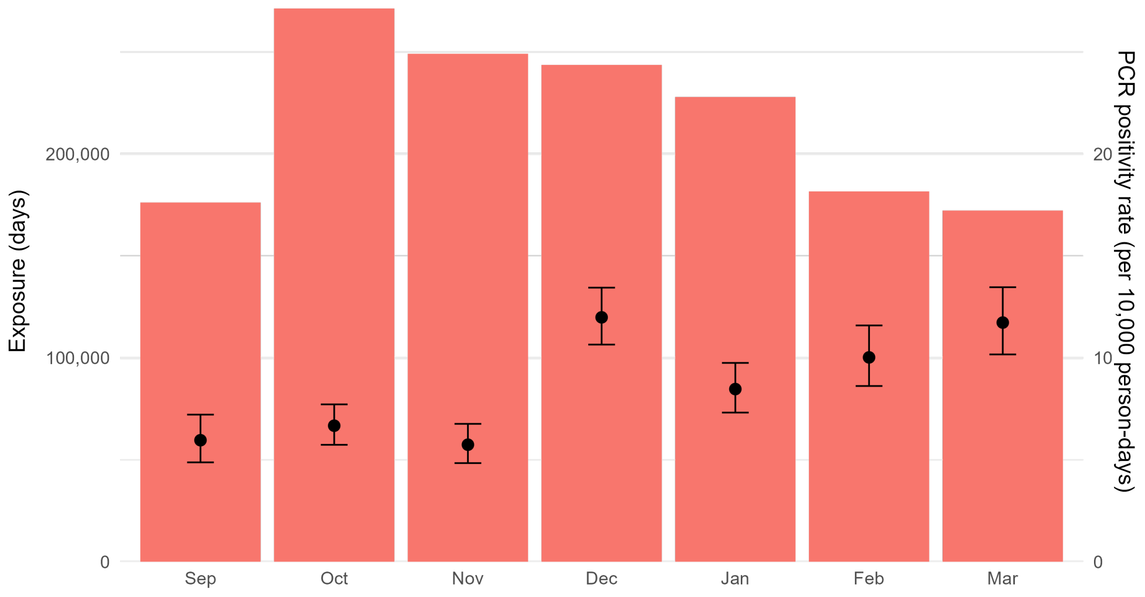

Figure S5: Exposure (person-days at risk) and crude PCR positivity rate per 10,000 person-days by month. Left-hand y-axis for exposure (bar chart), right-hand y-axis for crude PCR positivity (points and error-bars). Error bars show the 95% confidence interval (estimated using an exact Poisson method) around the estimated PCR positivity rate.

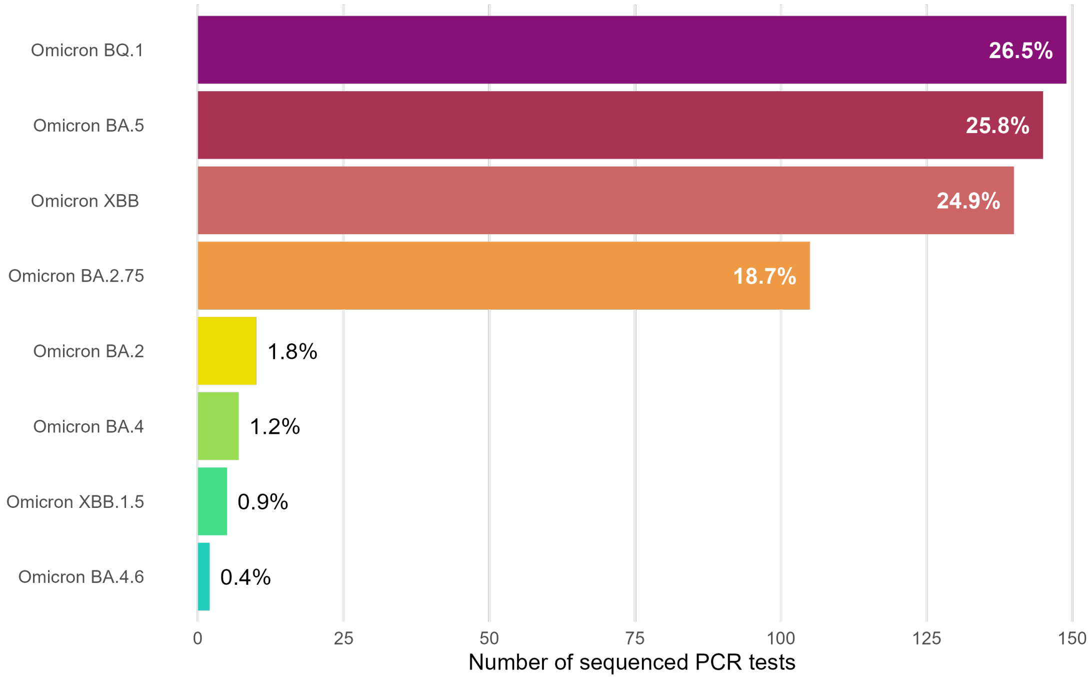

Figure S6: SARS-CoV-2 infections, by sequenced variant. Percentages indicate proportion of sequenced variants. Sequence information was available for 43.4% (563/1,298) of detected infections.

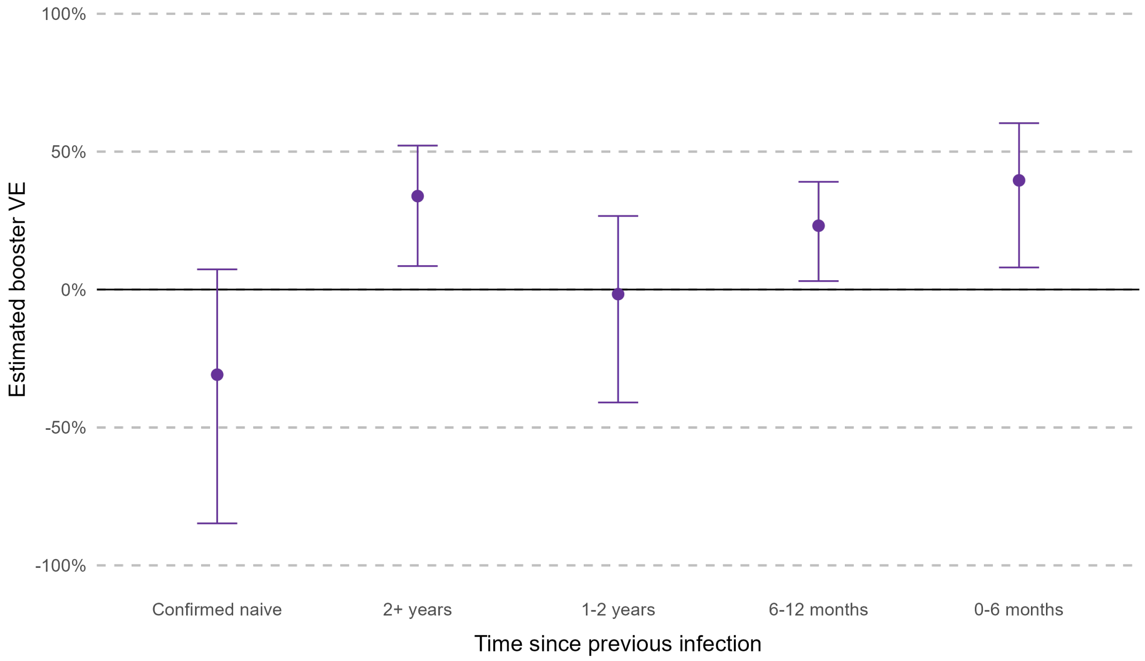

Figure S7: Estimated booster vaccine effectiveness (VE), relative to waned third dose, by time since previous infection (model 3). Error bars show the 95% confidence interval around the estimated VE.

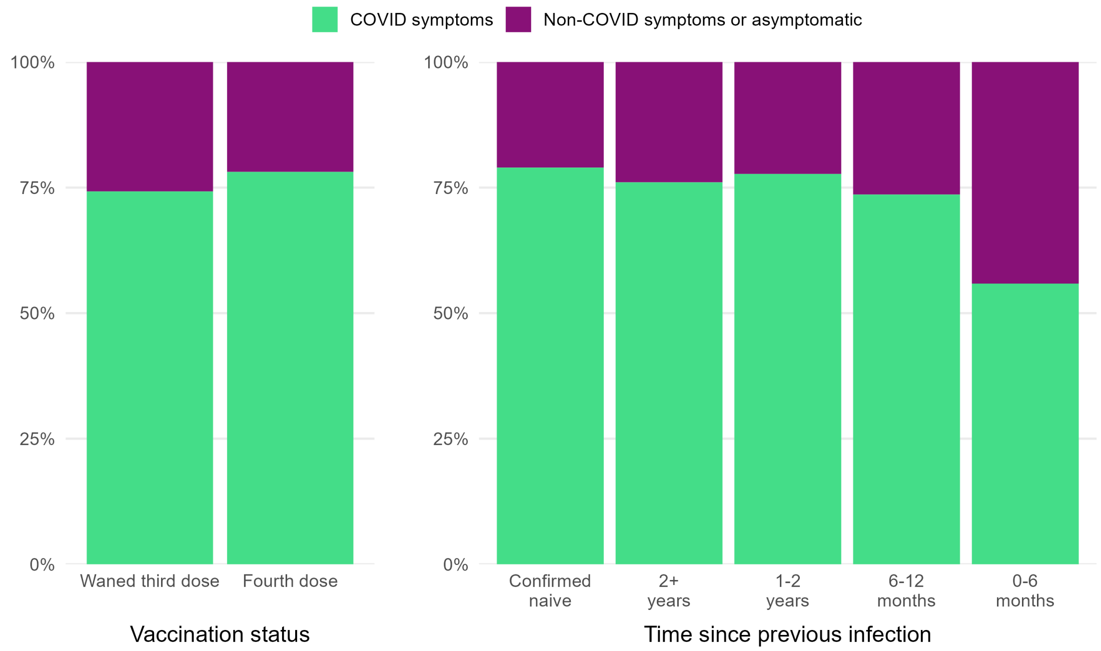

Figure S8: SARS-CoV-2 infections, by vaccination status, time since previous infection, and presence of COVID-19 symptoms.

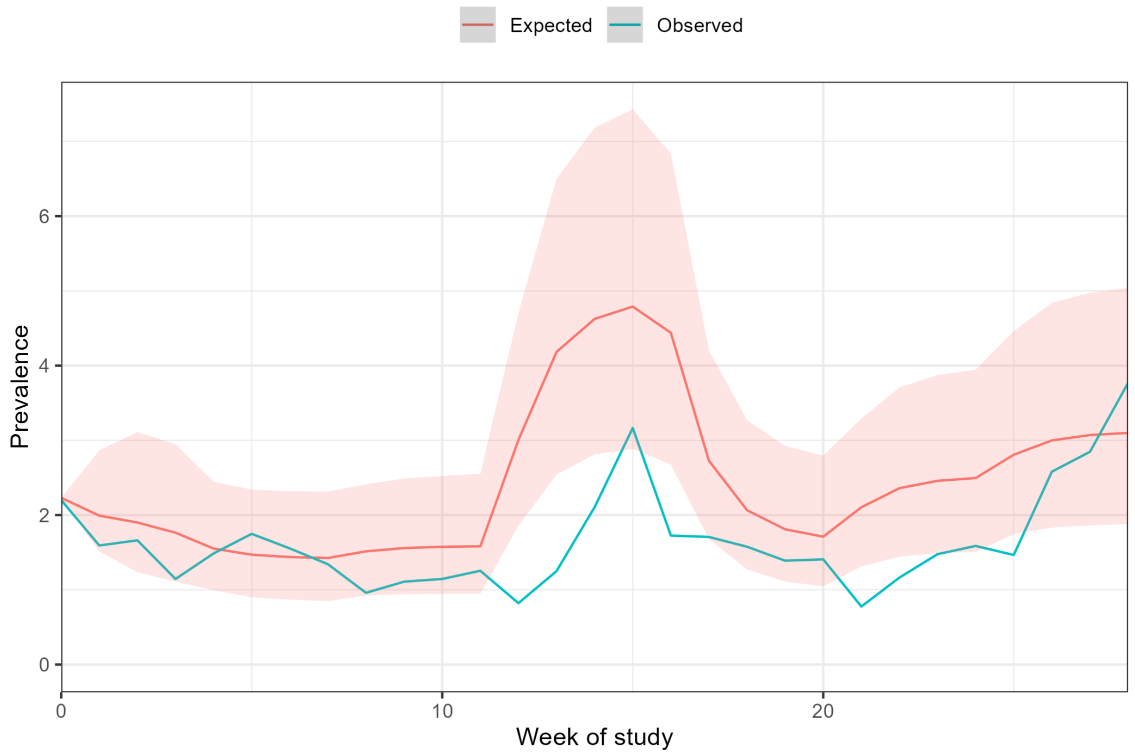

Figure S9: Comparison of expected (estimated) and observed prevalence in the infected state over time. Shaded area shows the 95% CI around the expected prevalence. The observed prevalence is below the lower 95% CI of expected prevalence in weeks 12-16. This period coincides with December and the new year period, when lower adherence to fortnightly testing was observed. This difference may be a result of the model accounting for infections that are unobserved due to the interval-censored nature of the data.

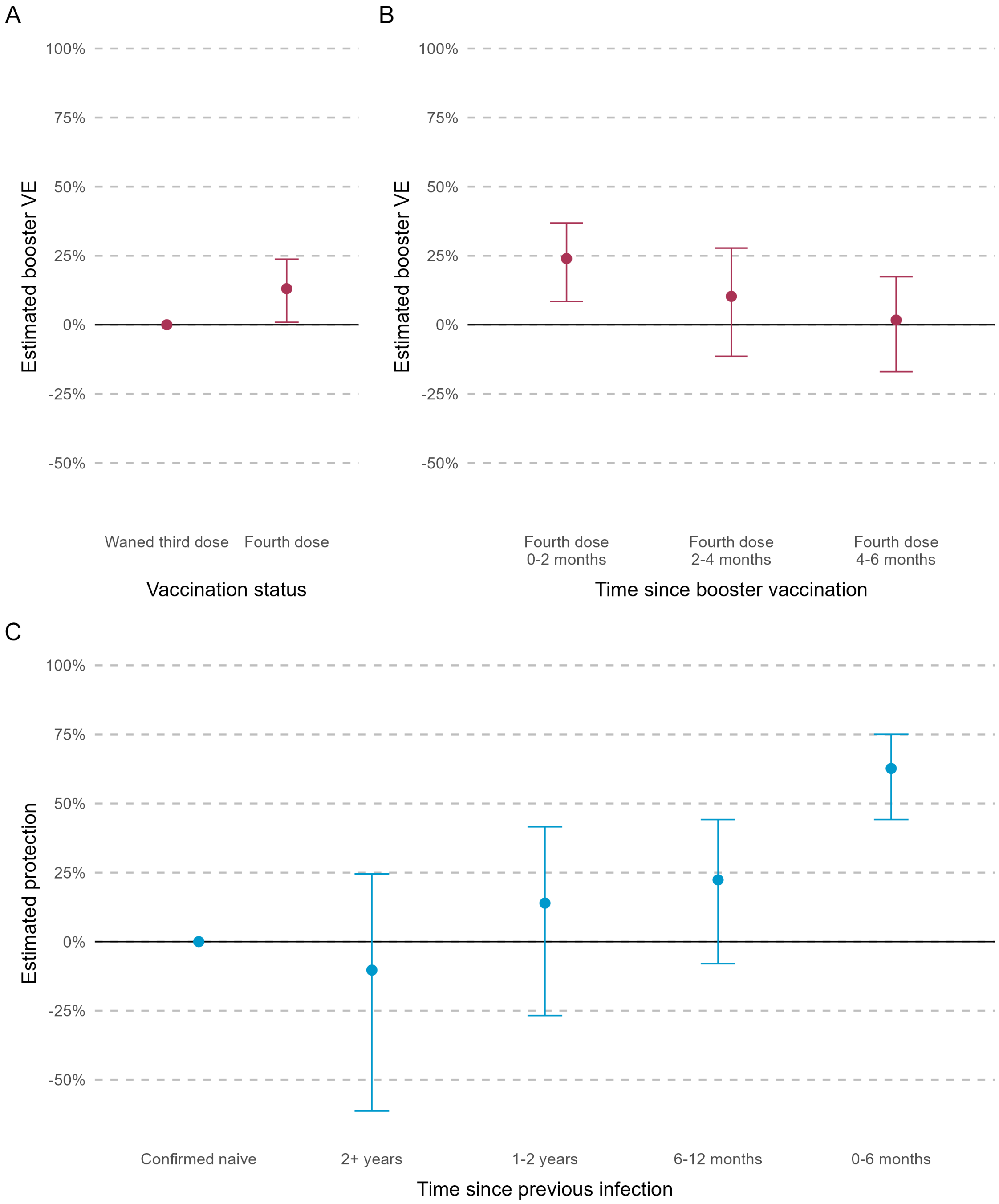

Figure S10: Estimated booster vaccine effectiveness (VE), relative to waned third dose, by booster vaccination status (panel A, model 1), time since booster vaccination (panel B, model 2), and estimated protection from previous infection, relative to a baseline of confirmed naïve (panel C, model 3). Error bars show the 95% confidence interval around the estimates.

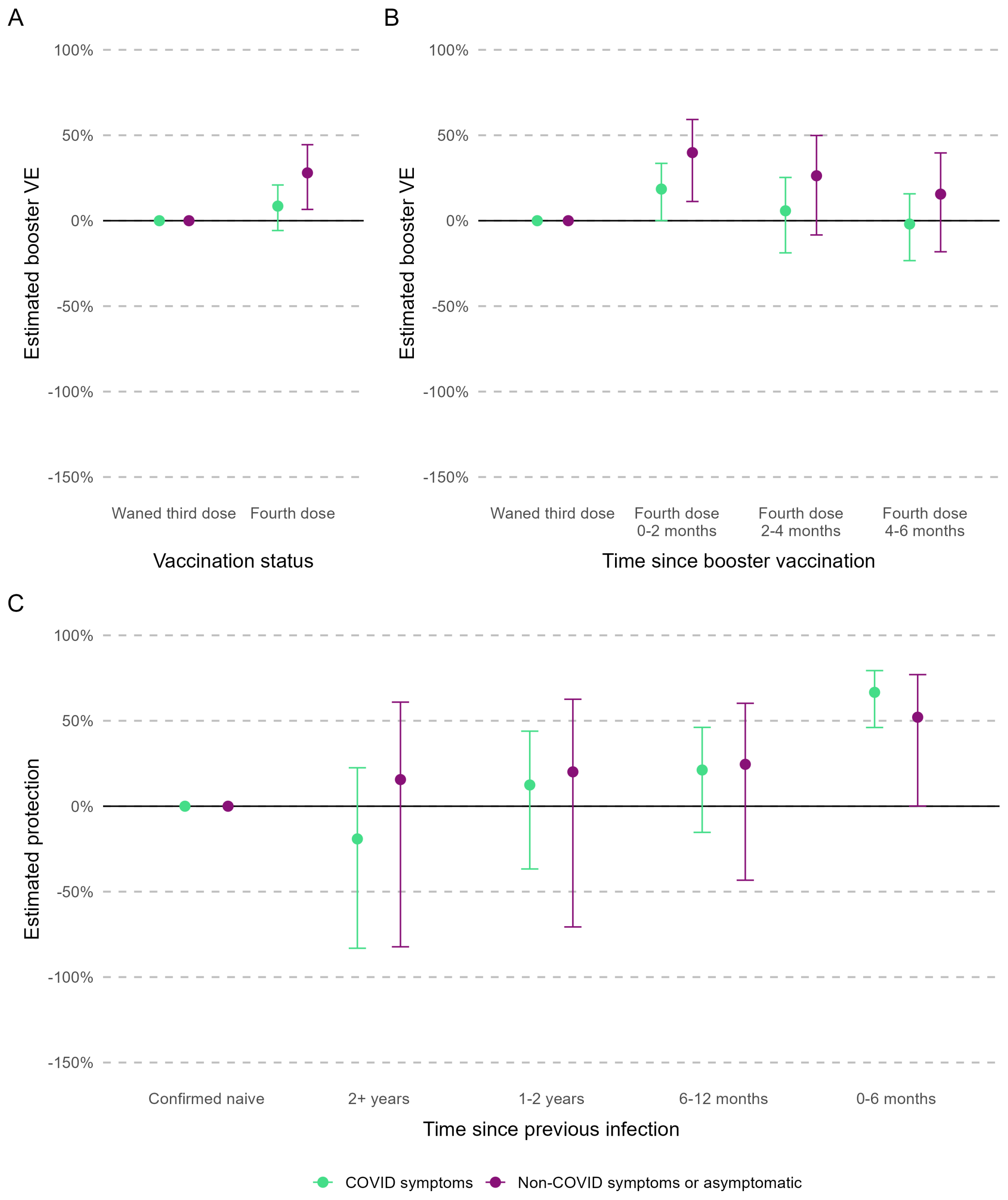

Figure S11: Estimated booster vaccine effectiveness (VE), relative to waned third dose by symptom status and booster vaccination status (panel A, model 5), time since booster vaccination (panel B, model 6), and estimated protection from previous infection, relative to a baseline of confirmed naïve (panel C, model 7). Error bars show the 95% confidence interval around the estimates.

4 Supplementary tables

Table S1: List of covariates used for multi-state models, with time-dependence and transitions used for each covariate.

**Covariate**	**Levels**	**Time-dependent**	**Transitions**
Month	Sep, Oct, Nov, Dec, Jan, Feb, Mar	Yes, piecewise-constant hazards	Infection only
Vaccination status	Third dose, Fourth dose (0-2 months, 2-4 months, 4-6 months)	Yes	Infection and recovery
Binary vaccination status	Third dose, Fourth dose	Yes	Infection and recovery
Time since previous infection	Confirmed naïve, 2+ years, 1-2 years, 6-12 months, 0-6 months	Yes	Infection and recovery
Age group	<25, 25-34, 35-44, 45-54, 55-64, 65+	No	Infection and recovery
Gender	Male, Female	No	Infection only
Region	East Midlands, East of England, ...	No	Infection only
Household status	Lives alone, Lives with others (no children), Lives with children	No	Infection only
Occupational setting	Ambulance, ED, Inpatient, Outpatient, ...	No	Infection only

Table S2: List of multi-state and Cox proportional hazards models used to generate estimates, with number of individuals, inclusion of symptom information, and list of covariates. Individuals without prior infection history and without a negative antibody test were excluded from analyses exploring time since infection, this is indicated by a smaller number of individuals for these models.
x: covariate included as main effect in regression, p: covariate included with piece-wise constant hazards, s: covariate included with stratification.

**Multi-state models**	Model 1	Model 2	Model 3	Model 4	Model 5	Model 6	Model 7
N (individuals in model)	9,560	9,560	7,549	7,549	9,560	9,560	7,549
Symptom data (Y/N)	N	N	N	N	Y	Y	Y
**Covariates**							
Month	p	p	p	p	p	p	p
Binary vaccination status	x		x		x		x
Vaccination status		x				x	
Time since infection			x	x			x
Vaccination:time since infection (interaction term)			x	x			x
Age group	x	x	x	x	x	x	x
Gender	x	x	x	x	x	x	x
Region	x	x	x	x	x	x	x
Household status	x	x	x	x	x	x	x
Occupation/Setting	x	x	x	x	x	x	x

**Cox proportional hazards models**	Cox model 1	Cox model 2	Cox model 3	Cox model 4
N (individuals in model)	9,560	9,560	7,549	7,549
Symptom data (Y/N)	N	N	N	N
**Covariates**				
Month				
Binary vaccination status	x		x	
Vaccination status		x		
Time since infection			x	x
Vaccination:time since infection (interaction term)			x	x
Age group	s	s	s	s
Gender	x	x	x	x
Region	x	x	x	x
Household status	s	s	s	s
Occupation/Setting	s	s	s	s

Table S3: Demographic characteristics of study participants.

**Characteristic**	**Overall**	**2+ years**	**1-2 years**	**6-12 months**	**0-6 months**	**No evidence of infection**	**Confirmed naïve**
All	9,560	1,105	1,153	2,378	2,140	2,100	773
**Gender**							
Male	1,524 (16%)	205 (19%)	180 (16%)	382 (16%)	346 (16%)	298 (15%)	113 (15%)
Female	8,036 (84%)	900 (81%)	973 (84%)	1,996 (84%)	1,794 (84%)	1,713 (85%)	660 (85%)
**Age group**							
<25	71 (0.7%)	5 (0.5%)	15 (1.3%)	24 (1.0%)	16 (0.7%)	9 (0.4%)	2 (0.3%)
25-34	828 (8.7%)	62 (5.6%)	95 (8.2%)	243 (10%)	222 (10%)	145 (7.2%)	61 (7.9%)
35-44	2,096 (22%)	211 (19%)	244 (21%)	691 (29%)	447 (21%)	376 (19%)	127 (16%)
45-54	3,702 (39%)	434 (39%)	456 (40%)	881 (37%)	824 (39%)	788 (39%)	319 (41%)
55-64	2,637 (28%)	371 (34%)	317 (27%)	501 (21%)	580 (27%)	625 (31%)	243 (31%)
65+	226 (2.4%)	22 (2.0%)	26 (2.3%)	38 (1.6%)	51 (2.4%)	68 (3.4%)	21 (2.7%)
**Ethnicity**							
White	8,611 (90%)	952 (86%)	1,005 (87%)	2,117 (89%)	1,958 (91%)	1,863 (93%)	716 (93%)
Asian	543 (5.7%)	88 (8.0%)	78 (6.8%)	157 (6.6%)	99 (4.6%)	86 (4.3%)	35 (4.5%)
Black	179 (1.9%)	35 (3.2%)	42 (3.6%)	35 (1.5%)	29 (1.4%)	29 (1.4%)	9 (1.2%)
Mixed-race	126 (1.3%)	14 (1.3%)	12 (1.0%)	43 (1.8%)	34 (1.6%)	18 (0.9%)	5 (0.6%)
Other	101 (1.1%)	16 (1.4%)	16 (1.4%)	26 (1.1%)	20 (0.9%)	15 (0.7%)	8 (1.0%)
**Medical conditions**							
None	7,038 (74%)	805 (73%)	855 (74%)	1,759 (74%)	1,582 (74%)	1,458 (73%)	579 (75%)
Immunosuppression	247 (2.6%)	37 (3.3%)	21 (1.8%)	60 (2.5%)	56 (2.6%)	51 (2.5%)	22 (2.8%)
Chronic Respiratory conditions	1,193 (12%)	123 (11%)	143 (12%)	323 (14%)	279 (13%)	249 (12%)	76 (9.8%)
Chronic Non-Respiratory conditions	1,082 (11%)	140 (13%)	134 (12%)	236 (9.9%)	223 (10%)	253 (13%)	96 (12%)
**Staff type**							
Administrative/Executive (office based)	1,613 (17%)	184 (17%)	185 (16%)	389 (16%)	338 (16%)	377 (19%)	140 (18%)
Doctor	1,155 (12%)	155 (14%)	125 (11%)	273 (11%)	288 (13%)	221 (11%)	93 (12%)
Nursing	3,178 (33%)	362 (33%)	388 (34%)	814 (34%)	699 (33%)	647 (32%)	268 (35%)
Healthcare Assistant	584 (6.1%)	82 (7.4%)	78 (6.8%)	144 (6.1%)	115 (5.4%)	115 (5.7%)	50 (6.5%)
Midwife	197 (2.1%)	22 (2.0%)	29 (2.5%)	47 (2.0%)	53 (2.5%)	33 (1.6%)	13 (1.7%)
Healthcare Scientist	434 (4.5%)	31 (2.8%)	46 (4.0%)	104 (4.4%)	98 (4.6%)	120 (6.0%)	35 (4.5%)
Pharmacist	268 (2.8%)	20 (1.8%)	34 (2.9%)	69 (2.9%)	59 (2.8%)	52 (2.6%)	34 (4.4%)
Physiotherapist/Occupational Therapist/SALT	404 (4.2%)	35 (3.2%)	55 (4.8%)	123 (5.2%)	98 (4.6%)	69 (3.4%)	24 (3.1%)
Student (Medical/Nursing/Midwifery/Other)	232 (2.4%)	61 (5.5%)	18 (1.6%)	55 (2.3%)	27 (1.3%)	66 (3.3%)	5 (0.6%)
Estates/Porters/Security	185 (1.9%)	14 (1.3%)	36 (3.1%)	41 (1.7%)	55 (2.6%)	32 (1.6%)	7 (0.9%)

Table S3 continued from previous page

Other (non-patient facing)	169 (1.8%)	18 (1.6%)	23 (2.0%)	33 (1.4%)	45 (2.1%)	34 (1.7%)	16 (2.1%)
Other (patient facing)	1,141 (12%)	121 (11%)	136 (12%)	286 (12%)	265 (12%)	245 (12%)	88 (11%)
**Occupational setting**							
Outpatient	3,841 (40%)	386 (35%)	450 (39%)	961 (40%)	995 (46%)	675 (34%)	374 (48%)
Ambulance/Emergency Department/Inpatient Wards	396 (4.1%)	39 (3.5%)	56 (4.9%)	116 (4.9%)	98 (4.6%)	47 (2.3%)	40 (5.2%)
Intensive Care	1,358 (14%)	173 (16%)	183 (16%)	380 (16%)	340 (16%)	174 (8.7%)	108 (14%)
Maternity/Labour Ward	303 (3.2%)	14 (1.3%)	29 (2.5%)	69 (2.9%)	95 (4.4%)	53 (2.6%)	43 (5.6%)
Office	829 (8.7%)	108 (9.8%)	90 (7.8%)	206 (8.7%)	108 (5.0%)	286 (14%)	31 (4.0%)
Other	2,244 (23%)	318 (29%)	254 (22%)	515 (22%)	371 (17%)	656 (33%)	130 (17%)
Patient facing (non-clinical)	502 (5.3%)	56 (5.1%)	78 (6.8%)	114 (4.8%)	120 (5.6%)	91 (4.5%)	43 (5.6%)
Theatres	87 (0.9%)	11 (1.0%)	13 (1.1%)	17 (0.7%)	13 (0.6%)	29 (1.4%)	4 (0.5%)
**Patient contact**							
Yes	7,992 (84%)	943 (85%)	983 (85%)	2,008 (84%)	1,796 (84%)	1,631 (81%)	631 (82%)
No	1,568 (16%)	162 (15%)	170 (15%)	370 (16%)	344 (16%)	380 (19%)	142 (18%)
**Deprivation index**							
Most deprived (1)	823 (9.1%)	99 (9.1%)	111 (10%)	217 (9.8%)	179 (9.0%)	160 (8.3%)	57 (7.8%)
Deprivation 2	1,528 (17%)	183 (17%)	210 (19%)	408 (18%)	312 (16%)	314 (16%)	101 (14%)
Deprivation 3	1,962 (22%)	232 (21%)	238 (22%)	476 (21%)	434 (22%)	428 (22%)	154 (21%)
Deprivation 4	2,121 (23%)	260 (24%)	259 (24%)	498 (22%)	474 (24%)	459 (24%)	171 (23%)
Least deprived (5)	2,593 (29%)	308 (28%)	275 (25%)	620 (28%)	587 (30%)	557 (29%)	246 (34%)
Missing deprivation information	533	23	60	159	154	93	44
**Region of residence**							
East Midlands	517 (5.4%)	78 (7.1%)	72 (6.2%)	124 (5.2%)	35 (1.6%)	182 (9.1%)	26 (3.4%)
East of England	1,176 (12%)	155 (14%)	172 (15%)	287 (12%)	296 (14%)	116 (5.8%)	150 (19%)
London	1,199 (13%)	202 (18%)	174 (15%)	326 (14%)	235 (11%)	212 (11%)	50 (6.5%)
North East	223 (2.3%)	26 (2.4%)	23 (2.0%)	58 (2.4%)	69 (3.2%)	41 (2.0%)	6 (0.8%)
North West	794 (8.3%)	117 (11%)	89 (7.7%)	208 (8.7%)	171 (8.0%)	143 (7.1%)	66 (8.5%)
Northern Ireland	499 (5.2%)	22 (2.0%)	60 (5.2%)	151 (6.3%)	146 (6.8%)	77 (3.8%)	43 (5.6%)
Scotland	1,701 (18%)	30 (2.7%)	177 (15%)	398 (17%)	683 (32%)	178 (8.9%)	235 (30%)
South East	947 (9.9%)	133 (12%)	88 (7.6%)	226 (9.5%)	110 (5.1%)	340 (17%)	50 (6.5%)
South West	958 (10%)	122 (11%)	101 (8.8%)	215 (9.0%)	80 (3.7%)	404 (20%)	36 (4.7%)
Wales	230 (2.4%)	25 (2.3%)	28 (2.4%)	56 (2.4%)	80 (3.7%)	18 (0.9%)	23 (3.0%)
West Midlands	700 (7.3%)	94 (8.5%)	111 (9.6%)	182 (7.7%)	115 (5.4%)	135 (6.7%)	63 (8.2%)
Yorkshire and the Humber	616 (6.4%)	101 (9.1%)	58 (5.0%)	147 (6.2%)	120 (5.6%)	165 (8.2%)	25 (3.2%)
**Household structure**							
Lives with others (no children)	4,834 (51%)	616 (56%)	567 (49%)	1,075 (45%)	1,089 (51%)	1,082 (54%)	405 (52%)
Lives alone	1,156 (12%)	123 (11%)	136 (12%)	223 (9.4%)	257 (12%)	282 (14%)	135 (17%)

Table S3 continued from previous page

Lives with others (with children)	3,570 (37%)	366 (33%)	450 (39%)	1,080 (45%)	794 (37%)	647 (32%)	233 (30%)

Table S4: Crude incidence calculations and protection from prior infection for selected covariates, by demographic characteristic.

**Characteristic**	**Number of participants**	**Positive PCR tests**	**Exposure (person-days at risk)**	**Crude PCR positivity per 10,000 person-days (unadjusted) (95% CI)**	**Protection relative to baseline (fully adjusted) (95% CI)**
All	9560	1298	1521928	8.53 (8.07, 9.01)	
**Age group**					
<25	71	2	9274	2.16 (0.26, 7.79)	60.06% (-61.15, 90.1)
25-34	828	131	118750	11.03 (9.22, 13.09)	-55.17% (-90.75, -26.23)
35-44	2096	305	325934	9.36 (8.34, 10.47)	-15.73% (-35.22, 0.95)
45-54	3702	498	594357	8.38 (7.66, 9.15)	Baseline
55-64	2637	335	435942	7.68 (6.88, 8.55)	7.84% (-7.84, 21.24)
65+	226	27	37671	7.17 (4.72, 10.43)	4.6% (-43.93, 36.77)
**Gender**					
Male	1524	193	244177	7.9 (6.83, 9.1)	Baseline
Female	8036	1105	1277751	8.65 (8.15, 9.17)	-9.83% (-29.54, 6.89)
**Ethnicity**					
White	8611	1191	1378760	8.64 (8.15, 9.14)	
Asian	543	64	81792	7.82 (6.03, 9.99)	
Black	179	20	27264	7.34 (4.48, 11.33)	
Mixed-race	126	12	19324	6.21 (3.21, 10.85)	
Other	101	11	14788	7.44 (3.71, 13.31)	
**Medical conditions**					
None	7038	955	1125891	8.48 (7.95, 9.04)	
Immunosuppression	247	31	38272	8.1 (5.5, 11.5)	
Chronic Respiratory conditions	1193	168	188356	8.92 (7.62, 10.37)	
Chronic Non-Respiratory conditions	1082	144	169409	8.5 (7.17, 10.01)	
**Staff type**					
Administrative/Executive (office based)	1613	197	258200	7.63 (6.6, 8.77)	
Doctor	1155	156	185499	8.41 (7.14, 9.84)	
Nursing	3178	437	499514	8.75 (7.95, 9.61)	
Healthcare Assistant	584	95	92807	10.24 (8.28, 12.51)	

Table S4 continued from previous page

Midwife	197	14	29522	4.74 (2.59, 7.96)	
Healthcare Scientist	434	66	72365	9.12 (7.05, 11.6)	
Pharmacist	268	51	43138	11.82 (8.8, 15.54)	
Physiotherapist/Occupational Therapist/SALT	404	53	63614	8.33 (6.24, 10.9)	
Student (Medical/Nursing/Midwifery/Other)	232	21	41009	5.12 (3.17, 7.83)	
Estates/Porters/Security	185	23	29218	7.87 (4.99, 11.81)	
Other (non-patient facing)	169	27	29060	9.29 (6.12, 13.52)	
Other (patient facing)	1141	158	177982	8.88 (7.55, 10.37)	
**Setting**					
Outpatient	3841	568	600914	9.45 (8.69, 10.26)	Baseline
Ambulance/Emergency Department/Inpatient Wards	396	65	58022	11.2 (8.65, 14.28)	-9.56% (-44.84, 17.13)
Intensive Care	1358	209	200329	10.43 (9.07, 11.95)	-14.19% (-35.1, 3.48)
Maternity/Labour Ward	303	47	50155	9.37 (6.89, 12.46)	10.06% (-22.79, 34.12)
Office	829	81	145247	5.58 (4.43, 6.93)	16.61% (-7.57, 35.36)
Other	2244	254	376276	6.75 (5.95, 7.63)	7.11% (-9.71, 21.36)
Patient facing (non-clinical)	502	58	76398	7.59 (5.76, 9.81)	1.92% (-30.01, 26.01)
Theatres	87	16	14587	10.97 (6.27, 17.81)	-83.43% (-204.47, -10.51)
**Patient contact**					
Yes	7992	1086	1266739	8.57 (8.07, 9.1)	
No	1568	212	255189	8.31 (7.23, 9.5)	
**Deprivation index**					
Most deprived (1)	823	112	129010	8.68 (7.15, 10.45)	
Deprivation 2	1528	194	241221	8.04 (6.95, 9.26)	
Deprivation 3	1962	257	310875	8.27 (7.29, 9.34)	
Deprivation 4	2121	282	333791	8.45 (7.49, 9.49)	
Least deprived (5)	2593	361	421315	8.57 (7.71, 9.5)	
**Region of residence**					
East Midlands	517	56	89779	6.24 (4.71, 8.1)	Baseline
East of England	1176	160	195308	8.19 (6.97, 9.56)	-1.24% (-41.2, 27.41)
London	1199	102	182727	5.58 (4.55, 6.78)	33.66% (4.85, 53.75)
North East	223	31	29820	10.4 (7.06, 14.76)	-0.77% (-61.97, 37.3)
North West	794	113	111247	10.16 (8.37, 12.21)	-38.68% (-96.56, 2.16)
Northern Ireland	499	89	80473	11.06 (8.88, 13.61)	-13.44% (-63.67, 21.38)
Scotland	1701	330	275189	11.99 (10.73, 13.36)	-18.65% (-62.68, 13.47)
South East	947	98	156100	6.28 (5.1, 7.65)	-1.04% (-44.54, 29.36)
South West	958	97	168695	5.75 (4.66, 7.01)	-9.22% (-56.33, 23.69)

Table S4 continued from previous page

Wales	230	58	39407	14.72 (11.18, 19.03)	-47.2% (-120.42, 1.69)
West Midlands	700	78	98077	7.95 (6.29, 9.93)	-1.9% (-48.78, 30.21)
Yorkshire and the Humber	616	86	95106	9.04 (7.23, 11.17)	-2.82% (-49.45, 29.26)
**Household structure**					
Lives with others (no children)	4834	632	777602	8.13 (7.51, 8.79)	Baseline
Lives alone	1156	161	187571	8.58 (7.31, 10.02)	1.2% (-18.94, 17.93)
Lives with others (with children)	3570	505	556755	9.07 (8.3, 9.9)	-4.38% (-20.03, 9.23)
**Month of study**					
Sep 2022	9559	105	176165	5.96 (4.87, 7.22)	
Oct 2022	9025	181	271290	6.67 (5.74, 7.72)	
Nov 2022	8515	143	249090	5.74 (4.84, 6.76)	
Dec 2022	8096	292	243641	11.98 (10.65, 13.44)	
Jan 2023	7669	193	227909	8.47 (7.32, 9.75)	
Feb 2023	6943	182	181605	10.02 (8.62, 11.59)	
Mar 2023	5772	202	172228	11.73 (10.17, 13.46)	

Table S5: Crude incidence calculations and estimates of relative vaccine effectiveness by vaccination status and time since previous infection.

Time since previous infection	Vaccination status	Number of participants	Positive PCR tests	Exposure (person-days at risk)	Crude PCR positivity per 10,000 person-days (unadjusted) (95% CI)	VE relative to baseline (fully adjusted) (95% CI)
Confirmed naïve	Waned third dose	755	51	45366	11.24 (8.37, 14.78)	Baseline
	Fourth dose	527	122	69799	17.48 (14.52, 20.87)	-30.87% (-84.79, 7.31)
2+ years	Waned third dose	1345	77	82640	9.32 (7.35, 11.65)	Baseline
	Fourth dose	1183	119	140143	8.49 (7.03, 10.16)	32.87% (8.51, 52.20)
1-2 years	Waned third dose	1679	83	80251	10.34 (8.24, 12.82)	Baseline
	Fourth dose	1845	126	115464	10.91 (9.09, 12.99)	-1.67% (-40.95, 26.66)
6-12 months	Waned third dose	3136	159	174120	9.13 (7.77, 10.67)	Baseline
	Fourth dose	2659	188	252695	7.44 (6.41, 8.58)	23.14% (3.07, 39.06)
0-6 months	Waned third dose	2851	45	121019	3.72 (2.71, 4.98)	Baseline
	Fourth dose	1779	38	133847	2.84 (2.01, 3.9)	39.58% (7.99, 60.32)

Table S6: Estimated time spent in PCR positive state, averaged across the study population, and empirical median duration of PCR positivity by vaccination status and time since previous infection.

	**Estimated days spent in PCR positive state (95% CI)**	**Empirical median duration of PCR positivity (initial PCR positive to subsequent PCR negative) [interquartile range]**
Whole population	7.51 days (6.94, 8.13)	15 days [12, 27]
**Vaccination status**		
Waned third dose	8.50 days (6.79, 10.64)	15 days [13, 28]
Fourth dose	6.90 days (5.87, 8.11)	14 days [12, 26]
**Time since previous infection**		
Confirmed naive	9.51 days (7.08, 12.78)	16 days [13, 28]
2+ years	9.16 days (7.16, 11.72)	14 days [12, 27]
1-2 years	7.44 days (5.86, 9,44)	14 days [12, 21]
6-12 months	6.56 days (5.63, 7.63)	14 days [12, 23]
0-6 months	7.25 days (6.32, 8.31)	16 days [14, 28]
**COVID symptoms**		
COVID symptoms	8.09 days (7.36, 8.90)	15 days [13, 27]
Non-COVID symptoms or asymptomatic	4.70 days (4.04, 5.47)	14 days [12, 23]

References

[1] P. K. Andersen and N. Keiding. "Multi-state models for event history analysis". en. In: *Stat. Methods Med. Res.* 11.2 (Apr. 2002), pp. 91–115. URL: http://dx.doi.org/10.1191/0962280202SM276ra.

[2] A. van den Hout. *Multi-State Survival Models for Interval-Censored Data.* en. CRC Press, Nov. 2016. URL: https://play.google.com/store/books/details?id=-xUNDgAAQBAJ.

[3] C. H. Jackson. "Multi-state models for panel data: the msm package for R". In: *J. Stat. Softw.* 38.8 (2011), pp. 1–29. URL: https://pdfs.semanticscholar.org/9913/436418e5b7448e6bc62157be9c28d8568f8b.pdf.

[4] J. D. Kalbfleisch and J. F. Lawless. "The Analysis of Panel Data under a Markov Assumption". In: *J. Am. Stat. Assoc.* 80.392 (Dec. 1985), pp. 863–871. URL: https://www.tandfonline.com/doi/abs/10.1080/01621459.1985.10478195.

[5] T Therneau. "A package for survival analysis in R". In: *R package* (). URL: https://github.com/therneau/survival.

[6] P. C. Austin. "A Tutorial on Multilevel Survival Analysis: Methods, Models and Applications". en. In: *Int. Stat. Rev.* 85.2 (Aug. 2017), pp. 185–203. URL: http://dx.doi.org/10.1111/insr.12214.

[7] M. Wolkewitz, M. von Cube, and M. Schumacher. "Multistate Modeling to Analyze Nosocomial Infection Data: An Introduction and Demonstration". en. In: *Infect. Control Hosp. Epidemiol.* 38.8 (Aug. 2017), pp. 953–959. URL: http://dx.doi.org/10.1017/ice.2017.107.

[8] C. H. Jackson, B. D. Tom, P. D. Kirwan, S. Mandal, S. R. Seaman, K. Kunzmann, A. M. Presanis, and D. De Angelis. "A comparison of two frameworks for multi-state modelling, applied to outcomes after hospital admissions with COVID-19". en. In: *Stat. Methods Med. Res.* 31.9 (Sept. 2022), pp. 1656–1674. URL: http://dx.doi.org/10.1177/09622802221106720.

[9] P. D. Kirwan, A. Charlett, P. Birrell, S. Elgohari, R. Hope, S. Mandal, D. De Angelis, and A. M. Presanis. "Trends in COVID-19 hospital outcomes in England before and after vaccine introduction, a cohort study". en. In: *Nat. Commun.* 13.1 (Aug. 2022), p. 4834. URL: http://dx.doi.org/10.1038/s41467-022-32458-y.

[10] M. J. Stensrud and M. A. Hernán. "Why Test for Proportional Hazards?" en. In: *JAMA* 323.14 (Apr. 2020), pp. 1401–1402. URL: http://dx.doi.org/10.1001/jama.2020.1267.

[11] V. Hall, S. Foulkes, F. Insalata, P. Kirwan, A. Saei, A. Atti, E. Wellington, J. Khawam, K. Munro, M. Cole, C. Tranquillini, A. Taylor-Kerr, N. Hettiarachchi, D. Calbraith, N. Sajedi, I. Milligan, Y. Themistocleous, D. Corrigan, L. Cromey, L. Price, S. Stewart, E. de Lacy, C. Norman, E. Linley, A. D. Otter, A. Semper, J. Hewson, S. D'Arcangelo, M. Chand, C. S. Brown, T. Brooks, J. Islam, A. Charlett, S. Hopkins, and SIREN

Study Group. “Protection against SARS-CoV-2 after Covid-19 Vaccination and Previous Infection”. en. In: *N. Engl. J. Med.* (Feb. 2022). URL: http://dx.doi.org/10.1056/NEJMoa2118691.

[12] J. M. Gran, S. A. Lie, I. Øyeflaten, Ø. Borgan, and O. O. Aalen. “Causal inference in multi-state models–sickness absence and work for 1145 participants after work rehabilitation”. en. In: *BMC Public Health* 15.1 (Oct. 2015), pp. 1–16. URL: https://bmcpublichealth.biomedcentral.com/articles/10.1186/s12889-015-2408-8.

[13] A. C. Titman and L. D. Sharples. “Semi-Markov models with phase-type sojourn distributions”. en. In: *Biometrics* 66.3 (Sept. 2010), pp. 742–752. URL: https://onlinelibrary.wiley.com/doi/abs/10.1111/j.1541-0420.2009.01339.x.

[14] J. Wei, P. C. Matthews, N. Stoesser, J. N. Newton, I. Diamond, R. Studley, N. Taylor, J. I. Bell, J. Farrar, J. Kolenchery, B. D. Marsden, S. Hoosdally, E. Y. Jones, D. I. Stuart, D. W. Crook, T. E. A. Peto, A. S. Walker, K. B. Pouwels, D. W. Eyre, and COVID-19 Infection Survey team. “Protection against SARS-CoV-2 Omicron BA.4/5 variant following booster vaccination or breakthrough infection in the UK”. en. In: *Nat. Commun.* 14.1 (May 2023), p. 2799. URL: http://dx.doi.org/10.1038/s41467-023-38275-1.
