# Supplementary material for: Effect of second booster vaccinations and prior infection against SARS-CoV-2 in the UK SIREN healthcare worker cohort
Source: Lancet Reg Health Eur. 2023 Dec 14;36:100809. doi: 10.1016/j.lanepe.2023.100809 (PMC10727938; doi:10.1016/j.lanepe.2023.100809)
Supplement: SIREN Protocol [file mmc2.pdf]

SIREN

SARS-CoV2 Immunity & Reinfection Evaluation

SIREN: The impact of detectable anti SARS-CoV-2 antibody on the incidence of COVID-19 in healthcare workers

Synopsis

Study title	Impact of detectable anti-SARS-CoV-2 on the subsequent incidence of COVID-19 in healthcare workers
Short title	SIREN (**S**arscov2 **I**mmunity & **RE**infection Evaluatio**N**)
Chief Investigator	Susan Hopkins
Sponsor	UK Health Security Agency
Funder	Department of Health and Social Care
Study coordination	UK Health Security Agency<br>Public Health Agency (Northern Irish Sites), Public Health Scotland (Scottish Sites), Public Health Wales (Welsh Sites)
Statistics and design	Andre Charlett

Study management group	Study design and management: Susan Hopkins, Victoria Hall, Colin Brown, Ana Atti and Jasmin Islam (Meera Chand 2020-21)<br>Statistics: Andre Charlett<br>Epidemiology: Victoria Hall, Sarah Foulkes (Mary Ramsay 2020-21)<br>Virology and serology: Maria Zambon, Tim Brooks, Ashley Otter, Amanda Semper, Michelle Cole (2021-22)<br>Data management: Sarah Foulkes, Jameel Khawam, (Anne Marie O'Connell 2020-21)<br>Genomics: Richard Myers and Eileen Gallagher<br>Information governance: Robert Kyffin (Samantha Organ 2020-21)<br><br>Devolved Administrations: Diane Corrigan (2020 Muhammad Sartaj) (Northern Ireland), Lesley Price (Josie Murray 2020) (Scotland), Elen de Lacy Chris Norman (Eleri Davies 2020) (Wales)
Study Design	Prospective cohort study to determine incidence of new SARS-CoV-2 infection in seronegative and seropositive staff working in healthcare organisations
Study participants	Staff working in healthcare organisations in the UK
Sample size	Up to 100,000 staff working in healthcare organisations
Planned study period	Eight years
Planned recruitment period	Nine months initial recruitment. Initial phase:12 months follow-up for each participant from enrolment; interval analysis of incidence and prevalence monthly and statistical analysis on primary outcome 3 monthly.
Study Protocol version	11.0

Contents

SIREN: The impact of detectable anti SARS-CoV-2 antibody on the incidence of COVID-19 in healthcare workers......1

Synopsis......1

Contents......3

Summary......5

1. Background......7

2. Aim and Objectives......9

3. Methods......11

3.1 Study design......11

3.2 Study sites......11

3.3 Inclusion/exclusion criteria......12

3.4 Sample size......12

3.5 Site set up......17

3.6 Recruitment......17

3.7 Enrolment and consent......18

3.8 Data collection and measurements......19

3.9 Reporting of results......24

3.10 Participant withdrawal......25

3.11 Storage of materials and additional Laboratory testing......26

3.12 Retention......27

3.13 Ongoing involvement beyond March 2023......27

3.14 SIREN 2.0......27

4 Data management......27

5. Analysis plan......29

6. Confidentiality and information governance......31

7. Quality assurance and research governance......33

Appendix 1: Collaborators......36

Appendix 2: Multiplex Respiratory Pathogens Sub-study 2022-2023......37

Summary:......37

Appendix 3: SIREN Qualitative Sub-Study and Evaluation......43

Appendix 4: Amendment History......44

References......51

Summary

COVID-19 is causing a global pandemic. This study aims to find out whether staff working in healthcare organisations who have evidence of prior COVID-19, detected by antibody assays (positive antibody tests), are protected from future episodes of infection compared to those who do not have evidence of prior infection (negative antibody tests). With the introduction of COVID-19 vaccinations for healthcare workers from December 2020, this study will also examine immunity acquired by a vaccine and obtain early estimates of vaccine effectiveness. It will explore both short and long-term effectiveness of a vaccine against infection and immunological response to a vaccine, including potential differences in response associated with factors such as prior exposure and antibody status.

In this study, we will recruit staff working in healthcare organisations who will be followed for at least a year and study their immune response to the virus causing COVID-19, called Severe Acute Respiratory Syndrome Coronavirus-2 (SARS-CoV-2). We will do this by collecting data on their history of COVID-19 infection and any new symptoms. All participants enrolled into the study are being asked to have a regular nose swab or a combined nose and throat swab (depending on local decision) every other week to detect mild cases or cases who do not have symptoms. This is the main test that is currently used to detect and diagnose infection by looking directly for the virus in the nose and throat. Once the infection is cleared, we cannot detect the SARS-CoV-2 virus in samples. Therefore, we will also ask these individuals to have blood samples taken every other week to determine whether they have antibodies to the infection. The frequency of swab tests and/or blood tests may be changed to between 1 and 4 weekly tests. This will depend on national and local epidemiology, study retention, feedback and results. These blood samples allow the previous infection to be detected as the response to infection in the body is to produce small particles in the blood called "antibodies". It takes up to 4 weeks to make enough antibodies to fight the infection. But once someone recovers, antibodies stay in the blood at low levels and this may help prevent us from getting infected with the same infection again. However, for SARS-CoV-2 infection, we do not know yet if the detection of specific antibodies is a correlate of protection from future infection. Through this study we will provide this very important information which will help to understand the future impact of COVID-19 on the population.

The first SIREN participants were recruited on 18 June 2020 and therefore ended their one-year follow up in June 2021. Insights from SIREN have proved vital for informing Government policy and have made significant contribution to the understanding of immunity to SARS CoV-2. The national and international need for this data remains high, particularly with the emergence of new variants and the need to understand the durability of immunity following infection and vaccination. Participants were therefore offered an opportunity to remain in SIREN for up to an additional 12 months, upon completion of their original follow-up period to maintain and strengthen the evidence base provided by SIREN.

Following this extension, those participants who continued with the study began completing their first extension period on 18th June 2022. The last SIREN participants to have been recruited and were offered the opportunity to remain in the study until March 2023. This acknowledged

the ongoing importance of the study which continues to perform a surveillance role within the United Kingdom.

SIREN Multiplex Respiratory Pathogens Sub-study

The government set out guidance for living with COVID-19, removing the requirement for social distancing measures and widespread use of face coverings, both of which likely reduced influenza transmission and therefore the incidence of influenza cases over the last two years. Australia had the most cases of influenza in the last 5 years in 2022 due to a reduction in social distancing and universal masking and it was anticipated that there would also be high numbers of cases in the United Kingdom this winter[13]. SIREN is well set up to study the impact of influenza, and other respiratory pathogens, with a cohort of highly motivated HCW who are used to regular testing and an established clinical and laboratory infrastructure to process these samples. This gives SIREN the unique opportunity to test for other respiratory pathogens as well as SARS-CoV-2 to investigate the incidence and impact on the HCW population.

It will be vital for policy makers to understand the impact of the different pathogens over winter in order to plan to ensure patient and healthcare worker safety. The SIREN study will therefore test for influenza A, influenza B and other respiratory pathogens where available in addition to SARS-CoV-2.

Please refer to Appendix 2: SIREN Multiplex Respiratory Pathogens Sub-study

SIREN 2.0

As the UK moves to a period where it learns to live with COVID-19, it has become increasingly apparent that COVID-19 will be endemic with continuing waves of infections. The dynamic of how COVID-19 interacts with the HCW population and its relationship with other pathogens is still unclear. Additionally, there remain important questions around the durability of protection from infection and vaccines and the impact of new variants, to inform future public health management, including vaccine policy, which SIREN, with its longitudinal design, is well placed to address.

SIREN is a versatile study, with important assets including an engaged cohort of healthcare workers, partnerships with NHS sites nationwide, a valuable biobank and database, and an agile multidisciplinary study team which can now be leveraged to deliver wider public health research and surveillance benefits. This was evidenced in winter 2022/23 with the introduction of the SIREN Winter Pressures sub-study and demonstrates the benefits of introducing sub-studies within the main SIREN protocol, to facilitate efficient expansion of the study to complementary themes to address emerging public health priorities.

Given these considerations, the study period for the SIREN study will be extended for a further five years, remaining live until 2028. There will not be regular testing (serology or NAAT) throughout this period, but the option to recommence testing for defined periods may be offered to participants when the scientific need arises. We will undertake data linkage to participants'

testing, vaccination and health records (e.g., hospitalisation). During this period engagement activities will continue, to preserve our cohort and sites. We anticipate introducing new questionnaires and setting up new sub-studies for participant and site involvement, which our existing participants and sites will be invited to join. All sub-studies will be subject to favourable REC review and in consultation with our designed SIREN Participant Involvement Panel.

Winter 2023-24 is the first period of enhanced interest where our healthcare cohort will be tested for multiple respiratory pathogens over an extended timeframe. The importance of the impact of COVID-19, Influenza and RSV on HCWs was demonstrated in the multiplex respiratory pathogens sub-study, however the influenza season in 2022-23 was unusually early, and so regular testing was not in place for the start of the season. For the 2023-24 season data collection will be in place earlier, with the cohort commencing testing before October and the multiplex respiratory pathogen sub-study cohort will be incorporated into the main study as SIREN 2.0.

1. Background

On 31st December 2019, the first cases of infection with a novel coronavirus, subsequently designated SARS-CoV-2, emerged in Wuhan, China.[1] A global pandemic was declared by the World Health Organisation (WHO) on 11th March 2020.[2] By 9th May, there were more than 3.6 million confirmed infections and 240,000 reported deaths globally.[3]

In order to determine the population impact of potential future waves of the pandemic of COVID-19, there is an urgent need to understand whether prior infection and any host immune response provides protection from future reinfection with SARS-CoV-2.

Currently, a confirmed diagnosis of infection of SARS-CoV-2, relies on laboratory diagnosis of infection using reverse transcriptase polymerase chain reaction (PCR). Diagnostic PCR typically targets the viral ribonucleic acid (RNA)-dependent RNA polymerase (RdRp) or nucleocapsid (N) genes using swabs collected from the upper respiratory tract (nose and throat).[4, 5] However as PCR only detects acute infection we are unaware of the true population prevalence of infection. While there is increasing evidence that a proportion of the population can be infected and asymptomatic, the true proportion of asymptomatic infection in the population is unknown.

Across the UK, some NHS organisations are choosing to screen healthcare workers with RT-PCR to identify individuals who are positive for SARS-CoV-2 and reduce the risk of healthcare worker (HCW) transmission to other HCW and patients through identification and appropriate exclusion from work. However, PCR from upper respiratory tract swabs may be falsely negative, due to quality or timing of collection; or due to viral titres in upper respiratory tract secretions peaking in the first week of symptoms,[6] but declining below the limit of detection in patients who present with symptoms beyond this time frame.[7] In individuals who have been infected and recovered, PCR provides no information about prior exposure or immunity. In contrast, assays that reliably detect antibody responses specific to SARS-CoV-2 could contribute both to diagnosis of acute infection (via rises in IgM and IgG levels) and to identify those who have been exposed and recovered with or without symptoms (via persisting IgG).[8,9] A further research gap is whether the detection of

an antibody response to SARS-CoV-2 will provide immunity to re-infection. In a study of medical students, reinfections with HCoV-229E were detected in 937 student years of follow-up; neutralizing antibody to 229E was common but it did not appear to influence the occurrence of, or likelihood of illness with, reinfection as judged by complement fixation seroconversion.[10] While IgG and IgM to SARS-CoV-2 can be effectively used to determine past exposure, it may be that these antibodies do not protect against future infection. The presence of neutralising antibodies may be required as these not only confirm prior infection but also prevent future infection. There are no commercial assays for live virus neutralisation assays at present but UKHSA and other university partners are developing in house assays to detect these specific antibodies. We will examine correlates of different types of antibody response and the protective effect against re-infection.

Therefore, this study will answer the key question on whether prior SARS-CoV-2 infection confers future immunity to SARS-CoV-2 re-infection in staff working in healthcare organisations who have a higher prevalence of infection than the general population; in one study 20% of asymptomatic London HCW tested for SARS-CoV-2 were positive on PCR tests over a 5-week period from 23rd March to 3rd May, with incidence peaking at 7.1% and declining 6 fold after 5 weeks.[11] It will also determine whether reinfection is due to a lack of neutralising antibody or waning immunity over time in individuals who are anti-SARS-CoV-2 positive, whether such individuals can be re-infected symptomatically or asymptomatically, about the incidence of new infection in individuals without prior exposure and the severity of COVID-19 in initial and subsequent episodes of infection if re-infection occurs. We will also use genomics to determine the viral diversity in staff working in healthcare organisations at each sampling point and particularly if individuals are re-infected.

Healthcare workers (HCW) have been identified as one of the first groups targeted for vaccination and therefore will be an important group within which to obtain early estimates for vaccine effectiveness[12]. They also may be a group whose vaccination details may not be easily identified in other data sources (such as GP data). Therefore, from December 2020, the scope of this study will include examination of immunity and re-infection associated with healthcare workers who have received a COVID-19 vaccine.

On 18th June 2021 the first SIREN participants reached the end of their 12-month follow up period. To ensure SIREN continued to provide critical insights into immunity to SARS-CoV-2, participants were offered the opportunity to take part in additional follow up to 31 March 2023. Follow-up testing continued to collect regular PCR and serology samples with methods and frequency subject to local site variation.

<u>SIREN After March 2023</u>

As the UK moves to a period where it learns to live with COVID-19, it has become increasingly apparent that COVID-19 will be endemic with continuing waves of infections. The dynamic of how COVID-19 interacts with the HCW population and its relationship with other pathogens is still unclear. Additionally, there remain important questions around the durability of protection from infection and vaccines and the impact of new variants, to inform future public health management, including vaccine policy, which SIREN, with its longitudinal design, is well placed to address.

SIREN is a versatile study, with important assets including an engaged cohort of healthcare workers, partnerships with NHS sites nationwide, a valuable biobank and database, and an agile multidisciplinary study team which can now be leveraged to deliver wider public health research and surveillance benefits. This was evidenced winter 2022/23 with the introduction of the SIREN Winter Pressures sub-study and demonstrates the benefits of introducing sub-studies within the main SIREN protocol, to facilitate efficient expansion of the study to complementary themes to address emerging public health priorities.

Given these considerations, the study period for the SIREN study will be extended for a further five years, remaining live until 2028. There will not be regular testing (serology or NAAT) throughout this period, but the option to recommence testing for defined periods may be offered to participants when the scientific need arises. We will undertake data linkage to participants' testing, vaccination and health records (e.g., hospitalisation). During this period engagement activities will continue, to preserve our cohort and sites. We anticipate introducing new questionnaires and setting up new sub-studies for participant and site involvement, which our existing participants and sites will be invited to join. All sub-studies will be subject to favourable REC review and designed in consultation with our SIREN Participant Involvement Panel.

The importance of the impact of COVID-19, Influenza and RSV on HCWs was demonstrated in the multiplex respiratory pathogens sub-study, however, the influenza season in 2022-23 was unusually early, and so, regular testing was not in place for the start of the season. For the 2023-24 season data collection will be in place earlier, with the cohort commencing testing before October. During this period the cohort will follow the same pathway for molecular testing and complete the same questionnaires, but there will be some differences in how serology is collected. This will now be incorporated into the main SIREN protocol under SIREN 2.0.

2. Aim and Objectives

Aim: The overall aim of this study is to determine if prior SARS-CoV-2 infection in health care workers confers future immunity to re-infection.

Objectives:

Primary: To determine whether the presence of antibody to SARS-CoV-2 (anti-SARS-CoV-2) is associated with a reduction in the subsequent risk of re-infection over short term periods (reviewed monthly), the next year and in the longer-term.

Secondary:

1. To estimate the prevalence of SARS-CoV-2, Influenza and RSV infections in staff working in healthcare organisations by region, using baseline serological testing at study entry and symptom history from January 1st 2020 to date of study entry
2. To estimate the subsequent incidence of symptomatic and asymptomatic SARS-CoV-2 infection and determine how this varies over time, using regular PCR testing (combined with any intercurrent symptomatic testing)

3. To estimate cumulative incidence of new respiratory pathogen infections in staff working in healthcare organisations stratified by age, sex, staff group, ethnicity and co-morbidities.
4. To measure the ability to culture viable SARS-CoV-2 virus from cases of re-infection diagnosed by PCR and whether those that are persistently positive on PCR are continuing to shed viable virus
5. To use genomic comparison to determine whether healthcare workers who become SARS-CoV-2 PCR-positive for a second time within a defined time frame are experiencing persistent infection or re-infection
6. To determine how respiratory pathogen serological response changes over time
7. To determine whether there is a relationship between serological response (using enzyme immunoassay detection of IgG) and the presence of neutralising (protective) antibodies
8. To identify serological, demographic or clinical factors that correlate with the presence of SARS-CoV-2 neutralising antibodies, including subsequent disease severity
9. To investigate the phylogenetic relatedness of SARS-CoV-2 viruses causing staff working in healthcare organisations infections
10. To monitor effectiveness of a vaccine/vaccines against respiratory infections and symptomatic disease
11. To monitor immune response to vaccination against COVID-19 and influenza over time
12. To estimate the incidence and burden of symptomatic and asymptomatic respiratory infections
13. To quantify the number of sick days off work either due to respiratory symptoms, confirmed SARS-CoV-2 or self-reported diagnoses of other respiratory pathogens

3. Methods

3.1 Study design

The overall study design is a prospective longitudinal cohort study of staff working in healthcare organisations within the UK.

3.2 Study sites

Any NHS organisation in the UK (including all four nations) who can deliver the requirements of the study will be eligible to participate. Primary care sites (GP surgeries or clinical commissioning groups (CCGs)) as well as independent healthcare providers, will be eligible to participate if they can demonstrate the ability to deliver the study.

Each site will be asked to invite eligible staff to participate. While initially we are not providing quotas, we may review the cohort of participants recruited initially to ensure appropriate representation by staff group, age, sex and ethnicity; quotas may be provided at that stage if certain groups are under-represented.

Organisations which fulfil the criteria of the sites described above will also be able to become Participant Identification Centres (PICs), where they will publicise the study to staff (using the methods outlined in this protocol), identify potential eligible participants and refer them to a study site. In order to be a PIC site the organisation will be expected to attend a Site Initiation Visit and complete a PIC agreement with the referral study site.

A subset of sites that are able to offer multiplex PCR will switch to this in Winter 2022-23 allowing detection of influenza A, influenza B, SARS-CoV-2 and any other respiratory pathogens on the local sites' panel. Sites unable to offer multiplex will continue offering dedicated SARS-CoV-2 testing. If sites are unable to continue to offer the requirements of the study described above UKHSA may offer a centralised system to facilitate participant retention.

From 1st April 2023 sites that have not yet closed out will be offered the option of becoming dormant SIREN sites for the next phase of the study (until 2028). As a dormant site they may be offered the opportunity to resume active testing/study activity and/or join sub-studies.

SIREN 2.0

Dormant SIREN sites will be approached to restart serology collection between 1st September 2023-31st March 2024. Where dormant sites agree to participate, all participants who have not withdrawn, regardless of whether they have extended previously, will be invited to join SIREN 2.0. Sites will continue to undertake provide local phlebotomy for serum sample collection. However, all samples will now be directly sent to UKHSA Porton Down for testing.. All participants will be tested using a multiplex PCR assay to detect SARS-CoV-2, Influenza A, Influenza B and RSV but this will be managed centrally, not at sites (see data collection). Where sites do not restart testing,

they can remain dormant. However, their participants may be offered the chance to undergo regular testing via a centralised pathway.

3.3 Inclusion/exclusion criteria

Inclusion criteria:

- **Healthcare organisation staff member who works in a clinical setting where patients are present**
  This includes all staff members who work on a site where patients are present – they do not need to be patient facing. They must work in the healthcare organisation but do not need to be employed by that organisation (working student healthcare workers or those employed by contracting organisations are eligible).
- **Can provide written consent** (this is collected in an online survey)
- **Is willing to remain engaged with follow-up for one year, even if they move to another healthcare organisation**

Exclusion criteria*

- **Written consent not provided**
- **Temporary short-term staff member**
  For study purposes this would be a staff member unlikely to remain at the healthcare organisation for at least three months, unless they are expected to transfer to another healthcare organisation; bank, agency and locum staff are eligible as long as they fulfil this criterion.

*While SIREN is happy to accept participants who are enrolled in other studies, individuals who are already enrolled in studies may not be able to participate if the other study does not accept co-enrolment into SIREN.

SIREN is collaborating with a number of wider immunology studies which are conducting further investigation into immunity response of SARS-CoV-2. SIREN participants may be invited to join one or more of these studies, in line with the relevant eligibility criteria. Data may be shared between SIREN and associated studies, in line with information governance and data protection procedures outlined in this protocol. A full list of associated studies can be found in Appendix 1.

3.4 Sample size

A simulation approach has been used to estimate the power to detect relative differences between the study cohorts.

It is anticipated that at least 115 NHS organisations will join this study. We are aiming for up to 100,000 staff working in healthcare organisations to be recruited. We have estimated that 25% of our cohort will be seropositive at enrolment healthcare (based on 20% of staff who were asymptomatic and tested positive in one London hospital between 23rd March and 2nd May 2020[11]).

For the longer-term outcomes of the study, the initial sample sizes were calculated on the basis of recruiting 10,000 participants from 40 sites. It has been assumed that a minimum of 250

participants would be recruited from each selected healthcare organisation, with a standard deviation of 50. The proportion of seropositive recruits at each site has been obtained from a Gaussian distribution with a mean of 0.25 and standard deviation of 0.05 to reflect expected inter-site variation. The cumulative incidence in each Site in the seronegative cohort has been simulated using Gaussian distributions with means of 0.05, 0.1, 0.2 and 0.3 each with a coefficient of variation of 0.2. This range represents that which is feasible to observe over a 12-month period, given the behavioural and social interventions still being employed during the study to control transmission.

A study duration of 52 weeks has been assumed with the inter-test period of 2 weeks. A total attrition of 35% of participants is assumed, unaffected by sero-status and occurring at a constant rate over the 52 weeks of the study. Attrition has been assumed to be independent of the infection process.

A range of cumulative incidence in the seronegative cohort has been used to reflect the immune effectiveness; with units in the simulations being allocated to be infected or not, using a draw from a Bernoulli distribution with p equal to the Site and cohort specific simulated cumulative infection rate. A simplifying assumption of a constant infection rate over the study period has been used.

For each scenario a set of 200 simulations were performed. For each simulation, the total number if infections and person weeks of follow up was calculated for each cohort in each organisation. This data was analysed using a mixed effects Poisson model, using the natural logarithm of the person weeks as an offset. Power was estimated as the proportion of simulations for which the Wald statistic p value for the estimated incidence rate ratio of the seropositive to seronegative cohorts was less than 0.05. These are presented in Table 1, indicating that there is sufficient power for all but the smallest immune efficacy of 0.1 i.e. a 10% reduction in incidence in the seropositive cohort. Such a small reduction is indicative of an irrelevant level of protection to provide a means of controlling the pandemic via natural herd immunity.

Table 1: Power estimates obtained via simulation for a range of immune effectiveness and cumulative incidence

Cumulative incidence in the seronegative at baseline cohort (per 100 participants) in 12 months	Immune Effectiveness 10%	Immune Effectiveness 20%	Immune Effectiveness 30%	Immune Effectiveness 40%	Immune Effectiveness 50%
0.05	0.15	0.44	0.79	0.98	1.00
0.1	0.20	0.77	0.99	1.00	1.00
0.2	0.53	0.99	1.00	1.00	1.00
0.3	0.67	1.00	1.00	1.00	1.00

To investigate the ability of a study of this size to detect differential immune efficacy in subgroups of healthcare workers, for example those aged over 50 or from a BAME group, a further set of simulations were undertaken. It has been assumed that the subgroup of interest is on average 35% of the total cohort size. The cumulative incidence used in the previous simulations has again been used with just those in the subgroup that are seropositive having an absolute reduction in immune efficacy of 5%, for example, if the cumulative incidence in the seronegative cohort is 0.3 (30%) and the immune efficacy in the non-sub group is 0.5 with a cumulative incidence of 0.15 (15%) in this group, the cumulative incidence in the seropositive sub group is 0.2 (0.15+0.05 20%). The interaction between cohort type and subgroup has additionally been added to the model and the estimated power obtained using the proportion of the 200 simulations for which this interaction had a Wald test p value of less than 0.05. Only immune efficacies of 0.3 or greater have been used, and these are presented in Table 2.

Table 2: Power estimates obtained via simulation for a range of immune effectiveness and cumulative incidence for detecting a sero-status by subgroup interaction

Cumulative incidence in the seronegative at baseline cohort (per 100 participants) in 12 months	Immune effectiveness 30%	Immune effectiveness 40%	Immune effectiveness 50%
0.1	0.93	0.91	0.98
0.2	0.70	0.82	0.84
0.3	0.67	0.68	0.69

In order to determine the outcome of immunity in much shorter intervals, by increasing the cohort recruitment to 100,000, we will be able to detect a difference between 0.05% and 0.1% cumulative incidence; even taking the incidence to as low as 0.02% in the seronegative group there is still excellent power of around 94%.

Estimated power for a two-sample proportions test

Pearson's chi-squared test

Ho: p2 = p1 versus Ha: p2 != p1

alpha	power	N	N1	N2	nratio	delta	p1	p2
.05	.9403	1.0e+05	25000	75000	3	.001	.001	.002
.05	1	1.0e+05	25000	75000	3	.002	.001	.003
.05	1	1.0e+05	25000	75000	3	.003	.001	.004
.05	1	1.0e+05	25000	75000	3	.004	.001	.005

If the incidence is lower than this, i.e. 0.05% in the seropositive group and 0.1% in the seronegative the power becomes sub-optimal at around 66%, but is sufficient for large differences.

Estimated power for a two-sample proportions test

Pearson's chi-squared test

Ho: p2 = p1 versus Ha: p2 != p1

alpha	power	N	N1	N2	nratio	delta	p1	p2
.05	.663	1.0e+05	25000	75000	3	.0005	.0005	.001
.05	.9933	1.0e+05	25000	75000	3	.001	.0005	.0015
.05	1	1.0e+05	25000	75000	3	.0015	.0005	.002
.05	1	1.0e+05	25000	75000	3	.002	.0005	.0025

Estimates for the vaccine effectiveness element are based on an assumed population of 40,000 participants.

Table 3 – Precision estimates assessing 95% CI around a vaccine effectiveness (VE) of 60% and 90%

Incidence in unvaccinated	Cases in unvaccinated	95% CI around VE of 60%	95% CI around VE of 90%
0.5	32	39-74	81-95
1	65	46-70	85-93
2	130	50-68	86-93
5	325	54-65	88-92

95% CIs will become narrower as VE increases (wider as it decreases) and also wider if coverage increases and in any strata. Overall the table shows that reasonable precision should be achievable.

For the initial vaccine effectiveness estimate three months post vaccine, the focus is on those seronegative at baseline.

Estimates for vaccine effectiveness are based on the following assumptions; 65% are seronegative at baseline, based on the baseline of 70% and assuming an additional 5% since this time. 75% are vaccinated and incidence during the 3 months in unvaccinated is 0.5%, 1%, 2%, 5%. This is based on incidence seen in September 2020 of 0.25% per 2 week (0.5% in a month or 1.5% in 3 month) to the incidence of 0.85% in 2 weeks in October (1.7% in a month or 5% in 3 months).

N=40,000 (26000 seronegative of whom 29,500 are vaccinated and 6,500 unvaccinated) is assumed.

SIREN 2.0

The reduction in the cohort size for SIREN 2.0 will result in more uncertainty around estimates of protection for the 2023 covid autumn booster campaign compared to precision previously achieved in SIREN where the cohort had reduced to below 10,000 due to drop out. The complexity of the statistical methods currently being applied in the estimation of protection from previous infections and vaccines preclude simple assessments of the statistical power.

Assuming an influenza vaccine uptake of 80% (https://www.gov.uk/government/statistics/seasonal-flu-and-covid-19-vaccine-uptake-in-frontline-healthcare-workers-monthly-data-2021-to-2022) in the SIREN 2.0 cohort and a cumulative influenza attack rate of 25% in the unvaccinated (https://www.ncbi.nlm.nih.gov/pmc/articles/PMC9006182/) then Table 4 below provides the precision that influenza VE of 40%, 50% and 60% can be estimated.

Table 4 – Precision estimates assessing 95% CI around an assumed vaccine effectiveness (VE) of 40%, 50%, and 60%

Assumed VE	Cases in unvaccinated	Cases in vaccinated	95% CI around VE of 90%
40%	250/1000	600/4000	31.7% - 47.3%

**50%**	**250/1000**	**500/4000**	**42.8% - 56.3%**
**60%**	**250/1000**	**400/4000**	**53.9% - 65.3%**

Thus, precision of between +/- 5% and +/-10% are achievable for end of season VE estimates under the assumptions used. There is likely to be some attrition during the 2023/24 winter period which will result in some inflation of the precisions presented in Table4.

3.5 Site set up

A site initiation visit (SIV) will take place by conference call for each study site. During the SIV, a member of the central study team will describe the study methods and discuss possible ways to organise recruitment and data collection with the local clinical and research team. An electronic Recruitment Log (a participant linelist on a formatted MS Excel spreadsheet) will be provided to all sites to record details on all recruited participants. The recruitment log will be pre-populated with study number and passcode**.** Both fields are unique codes required by participants to access and complete the online consent form and enrolment questionnaire. Study sites will be responsible for updating and securely storing this information in the recruitment log and for securely transferring it to UKHSA SIREN team and/or the appropriate Devolved Administration public health organisation's SIREN team securely if requested.

3.6 Recruitment

All recruitment should take place as quickly as possible, although UKHSA have confirmed an extension of recruitment to 31st March 2021. Participants are recruited by an all-staff communication requesting volunteers, but sites may choose to use locally appropriate publicity methods, e.g. posters, social media, intranet, stalls etc. Local organisations can produce their own publicity materials, although UKHSA have developed approved posters for use.

The organisation's research team will be responsible for assessing eligibility and ensuring participants are informed about the study requirements, ensuring all participants receive a participant information leaflet prior to recruitment. The research team should then provide interested and eligible staff with a unique study number, passcode and the webpage to complete the online consent form and enrolment questionnaire, and their details should be recorded in the Recruitment Log. Participants will not be able to access the online survey without these two fields, which can only be provided by their local research team. Care should be taken by the local organisation's research team to ensure that the unique study number and passcode combination are not issued to multiple potential participants, and if once given to one individual should never be re-allocated (even if never used).

Following the introduction of a vaccine, all vaccinated healthcare workers should receive a leaflet informing them about SIREN and that someone from their local research team may be in contact to discuss enrolment. If people wish to opt out of this contact they can do so via local processes set up at each site, e.g. email.

Screening, eligibility assessment and formation of the cohort at each hospital:
For each participant, a member of the healthcare organisation's research team will assess eligibility and monitor the proportion of participants from different occupational groups; they may choose to send out further communications to support recruitment to under-represented groups. The healthcare organisation may also choose to enrich their seropositive participants at baseline, particularly if they have identified low seroprevalence among staff working in their healthcare organisation. This may be done by general communication encouraging staff who know they are seropositive to enrol, or by targeted communication to known seropositive staff, if this data is held and accessible according to local organisational protocols. The study is observational only and there is no randomisation or allocation to any subgroups.

Staff such as junior doctors may have planned moves to other healthcare organisations within the study period. Where the participant is moving to a new organisation participating in SIREN, it is the responsibility of the original enrolling organisation's research teams to transfer these participants (including PII) directly to the new sites and communicate this transfer to the SIREN team. The participant should keep their original study number for the study duration. Where staff may be moving to a healthcare setting not participating in SIREN, they can remain enrolled, subject to the approval of the principal investigator and their original enrolling organisation continuing to offer them testing. Frequency of testing for these participants can be flexible. Participants who are leaving healthcare practice (e.g. on retirement) can also remain enrolled in the study, subject to the approval of the site's principal investigator.

3.7 Enrolment and consent

Participants will be enrolled in SIREN once they have submitted the online consent form and enrolment questionnaire, which requires them to provide their contact details for follow-up communication.

Consent will be undertaken online as the first part of the enrolment questionnaire, with participant initials requested against each point. A link to a copy of the consent form will be automatically sent to both the participant and study team on submission of the enrolment consent form and questionnaire.

The local organisation's SIREN study team will check the validity of the responses of the consent form on receipt. If a participant has provided responses 'Yes' or 'Y', or 'x' throughout, this can be accepted as valid consent. In the event that the participant has given a response which is ambiguous (e.g. 'N', 'No' or '?'), the local study team will contact the participant, clarify whether they intended to consent and obtain a paper copy of the consent form or facilitate withdrawal. They will send a secure email to the central UKHSA study team containing participant study number and the steps taken to ensure valid consent (they do not need to transfer the consent to UKHSA).

Where the UKHSA enrolment questionnaire is not functioning (e.g. server down or internet not working), the research team may obtain consent using a paper copy of the consent form and proceed to obtain bloods, but must follow up and ensure that the participant has completed the enrolment questionnaire within 48 hours. This paper consent form does not need to be sent to the UKHSA SIREN team but should be retained securely at the site. A paper copy of the consent form is only to be used in exceptional circumstances as outlined above.

A supplementary consent form has been designed for participants previously recruited into UKHSA seroprevalence surveys, as detailed in section 6.

At the end of their 12-month follow-up period, active participants may be given the option to participate in an additional follow-up period of up to 12 months. Explicit consent will be obtained for one of three options; to end all involvement with SIREN, to continue with active follow-up or to continue with passive follow-up only. Passive follow-up will involve no further collection of SIREN samples but permits SIREN to link existing study data, including to potential future test results not collected as part of SIREN. This consent will be collected electronically via Snapsurvey and is not conditional to a participant ending their active involvement with SIREN. Where possible, participants will be notified of this choice in advance of the end of their original follow-up period. In the event of the online questionnaire being unavailable, a paper copy of consent to extended follow-up may be collected via sites following the process for consent outlined above.

<u>SIREN 2.0</u>

All participants who had not previously withdrawn were offered the opportunity to withdraw or cease data linkage in April 2023. Participants who did not withdraw have remained as part of the cohort and can be approached for further studies.

Participants who were part of the multiplex respiratory pathogens sub-study in 2022-23 were invited to continue regular testing over the summer of 2023. They will be enrolled into SIREN 2.0 using an opt- out form. Participants at dormant sites who agree to offer local serology testing who have not withdrawn will be offered to join SIREN 2.0, and will complete an opt in form. Participants who are at dormant or closed sites who have not withdrawn will also be offered to join SIREN 2.0 via an online consent form.

3.8 Data collection and measurements

3.8.1 Baseline data

Self-completed enrolment questionnaire: The participant will self-complete an online survey at enrolment. Baseline data includes demographic/social factors (gender; date of birth; ethnicity; smoking history); comorbidities including whether immunocompromised; job role; potential exposures to COVID-19; history of COVID-19 symptoms and testing since January 2020.

Baseline testing: Upon receipt of the consent form, the local organisation's research team will organise for the participant to attend appointment(s) to provide blood samples and swabs for viral testing. Ideally these samples will be provided within 48 hours of the participant's completion of the enrolment questionnaire; up to 5 days after enrolment will be accepted as an outer limit. If a participant has submitted samples for an antibody test up to 5 days prior to enrolment, this can count as their baseline antibody test, as long as there is sufficient serum available to be sent to UKHSA. Any samples for baseline testing taken over 5 days following enrolment should be recorded as a protocol deviation. Where possible, a baseline sample should be collected prior to receiving a vaccine, however a vaccine should not be delayed to accommodate this.

SARS-CoV-2 molecular screening: This will be undertaken according to the local organisation procedure for screening healthcare workers or through a protocol set up specific for this study. Screening will be performed at regular intervals from 1 to 4 weeks (as specified by the UKHSA SIREN study team) and at any point the participant has clinical symptoms that meet local criteria for testing. It may be a nose swab or a nose and throat swab and may be undertaken by self-swabbing or administered by a trained member of staff depending on the local organisational preference. The swab will be tested for SARS-CoV-2 RNA in an accredited laboratory (or those who have provided satisfactory verification documents), which may be the clinical laboratory or through the national testing programme, using a validated nucleic-acid amplification test (NAAT), such as RT-PCR. Where lateral flow device (LFD) tests are used, all positive LFD tests should be confirmed using a validated NAAT such as RT-PCR.

As this is a clinical sample that requires reporting of the result individually, full standard identifiers will be used. Residual specimen and any available nucleic RNA extracts should be stored at -80°C.

Whole viral genome sequencing should be performed on SARS-CoV-2 positive specimens. Where sequencing is not available locally, please follow the latest guidance and send SIREN samples preferably to UKHSA Colindale, or to your linked sequencing laboratory. The SIREN central study team can link sites to a sequencing laboratory if needed.

Anti-SARS-CoV-2 antibody testing: Each participant will have a venous blood test on the same day as the PCR screening (within 48 hours is acceptable). The local SIREN team will determine the optimal procedure within each healthcare organisation, which may be visiting a study nurse, attending the phlebotomy service with a pre-designed form, or ward teams/peer-to-peer blood testing is also acceptable if this is approved by local governance procedures and those taking blood have been appropriately trained. Up to 10ml venous blood sample will be taken (in one or more blood tubes) and serum will be separated. For participants in wider immunology studies, the volume of some blood draws will be greater, up to 100ml (typically 50-60ml). No more than 470ml blood will be taken in a 16-week rolling period. The volume required for local testing will be removed and the residual volume should be retained in the form of one full 2ml aliquot for use as below. The aliquot for local testing will be tested immediately in the organisation’s local laboratory using an immunoassay. As this is a clinical sample that requires reporting, full standard identifiers will be used.

The second aliquot will be stored for a maximum of 7 days at 2-8°C, or at -20°C or below for longer, and batch shipped to UKHSA for later analysis using alternative assays to establish correlates of protection and compare the serological response results across different assays. This will be completed initially for 10,000 participants to validate the assays, and the composite UKHSA serological assessment will be used to assign these participants to the seronegative or seropositive cohort at baseline. The results of this validation on 10,000 participants will inform whether future participants also receive composite UKHSA serological assessment or are assigned a serological status based on their local results (potentially adjusted for assay-specific validation results).

This additional UKHSA serological evaluation will be stopped or adjusted when the study team are of the view that there is sufficient data to predict the relationship of the commercial assay result to the serological characterisation. A small seropositive sub-cohort will be selected to follow for a detailed assessment of antibody dynamics.

Participants may be offered additional testing via the NHS Test and Trace testing platform using a finger prick collection method, if the test is not offered as standard by their trust. This optional test would follow procedures as agreed with the national testing platform and by offered in addition to the venous blood sampling described above.

SIREN 2.0

All participants will complete a new online baseline questionnaire. This will update demographic/social factors including, job role, where they work, the number of people in their household and any medical conditions that lead to an increased risk for respiratory infections.

3.8.2 Follow-up data

Participants will be asked to complete follow-up surveys and provide swabs and blood samples for repeat testing at regular intervals.

Follow up questionnaire: The participant will receive a unique link sent to their mobile phone or email address to complete their follow-up questionnaire. Initially the follow-up questionnaire will be sent every two weeks. The follow-up questionnaire will collect information on possible respiratory infection symptoms or exposures, risk factors, enrolment in COVID-19 vaccine/treatment trials, sick days taken due to respiratory illnesses and any COVID-19 or seasonal influenza vaccine during the follow-up interval.

Frequency of follow up testing visits: At the initiation of the study this will occur every two weeks for every site. The frequency may be altered to be more or less frequent (1 - 4 week intervals). This will depend on national and local epidemiology, study retention, feedback and results. This decision will be reviewed by the central UKHSA SIREN Study Management Group and, if and when, a decision is made, will be communicated to sites. Follow-up visits should not occur more than two days before the scheduled date or more than five days afterwards. If participants are unable to attend for testing within this period (e.g. annual leave), this should be recorded as a missed visit. For their next testing appointment, they can either be started on a new 14-day test cycle or returned their old one. Unless there is a clinical reason to do so, participants should not be tested more than once in a 7-day period for the purpose of SIREN.

Follow-up molecular testing: Procedures for follow-up SARS-CoV-2 RT-PCR (or equivalent NAAT) is the same as baseline.

SARS-CoV-2 viral culture may be attempted for those individuals who are persistently NAAT positive, have potentially been re-infected or have been infected since receiving a vaccine; this

may involve UKHSA contacting local research teams and requesting extra swab samples from these participants if there is no suitable residual sample available. Where this is required the participant may need to complete a test at home (as may be self-isolating). We would also request local organisation research teams to set-up procedures with their laboratory to identify these events of interest and report them to UKHSA SIREN team to ensure timely follow-up. Extra swab tests may also be requested for confirmatory or further testing.

Healthcare organisation research teams are expected to actively monitor testing attendance and test results in their cohort, to follow-up repeat non-attenders, and to alert the UKHSA SIREN team to events of interest (i.e. a possible re-infection, persistent infection or vaccine breakthrough). This may involve working with Virology/Microbiology and Occupational Health, depending on local procedures. If a participant misses eight consecutive scheduled testing visits or four months of visits (whichever is shorter) this should be recorded as a protocol deviation.

Follow-up serology

All samples should be tested locally for SARS-CoV-2 antibodies. Whilst sites are requested to retain baseline sera from all participants, at follow-up, sites are only asked to retain and send to UKHSA the sera from participants:

- (First samples for all participants)
- Who are seropositive for SARS-CoV-2;
- Who have previously been seropositive for SARS-CoV-2 (including prior to the study commencing if known);
- Who have been PCR positive for SARS-CoV-2 at any point (including prior to the study commencing if known);
- Any participant who has been vaccinated against SARS-COV-2 should also have their serum retained from the point of vaccination onwards.
- Participants where UKHSA SIREN team has contacted the local healthcare organisation directly to request samples
- Where groups of interests have been identified by SIREN and its scientific consortium partners their sera may be specifically requested for additional testing.

The laboratory manual includes details on how to manage this process.

Samples should be shipped in monthly (or more frequent if required) batches to UKHSA for further characterisation (see laboratory manual for directions).

Extended follow-up until March 2023

The extended follow-up period will continue to collect study data as outlined in the main follow-up, i.e. follow-up survey, PCR testing and serology testing. These may be at reduced frequencies or via different collection methods. Specific testing schedules and procedures may differ by site and will be dependent on local arrangements at each trust, as outlined in the site manual. Serological follow-up may be at a reduced frequency compared to the main follow-up period but

should occur at a minimum of every three months. PCR testing should occur at a minimum of monthly and may continue to take place in a supervised environment at sites, or via a centralised national postal testing.

3.8.3 Event of Interest up to 31st March 2023

Once a SARS- CoV-2 event of interest is detected within the SIREN study, an investigation flow is flagged. This may involve requests for additional information and testing.

3.8.4 Qualitative data

Where specific themes of interest are highlighted through data collected or direct feedback from sites and participants further qualitative research may be conducted to explore them further. This may involve focus groups, feedback forms, structured interviews or other standard methods of gathering qualitative data. This data may be collected from participants, sites or other professionals impacted by SIREN data (for example infection control teams, hospital management and occupational health departments).

For further methodology see Appendix 3.

3.8.5 SIREN 2.0

Participants will continue to be asked to complete follow-up surveys and provide swabs and blood samples for repeat testing at regular intervals; however, these are different from the original protocol and thus outlined below.

Follow-up questionnaire

The participant will receive a unique link sent to their mobile phone or email address to complete their follow-up questionnaire as in the original protocol. Questionnaires will be sent every 2 weeks. The follow-up questionnaire will collect information on any symptoms or exposures to respiratory infections, sick days taken, any COVID-19 or seasonal influenza vaccine during the follow-up interval and any antibiotic use.

Follow-up molecular testing

All participants will be tested using the same postal pathway as that used by the multiplex respiratory viruses sub study._Participants will be asked to complete the swab every 2 weeks on the same day they complete their questionnaire. The frequency may be altered to be more or less frequent (1–4-week intervals)._Participants will receive self-swab kits by mail and will package and mail the kits back to a central laboratory or laboratories. Testing will be co-ordinated by the central SIREN team and performed by a UKHSA approved laboratory. The swab will be tested for SARS-CoV-2, Influenza A, Influenza B and RSV RNA but may also be tested for any RNA or DNA from any other respiratory pathogen available in the accredited laboratory using a validated nucleic-acid amplification test (NAAT), such as RT-PCR. Residual specimen and any available nucleic RNA extracts should be stored at -80°C.

All samples positive for SARS-CoV-2 will be sent for viral whole genome sequencing. Samples positive for other respiratory pathogens will be stored -80°C and may be tested in the future.

Follow-up serology

Teams at local SIREN sites who agree to recommence serology testing will be asked to arrange regular venepuncture for sera. The local SIREN team will determine the optimal procedure within each healthcare organisation, as per the baseline protocol. Up to 10ml venous blood sample will be taken (in one or more blood tubes) and serum will be separated locally. Sites will not test locally but will be store samples for a maximum of 7 days at 2-8°C, or at -20°C or below for longer, and batch ship to UKHSA Porton Down for testing. Participants will be bled at up to four points between September 2023-March 2024.

Participants may also be offered self-collected testing such as by finger-prick testing to supplement sampling at sites.

Events of Interest

This is a responsive study and so the definition of an event of interest is liable to be adapted over the study. Whilst previously we focused on SARS-CoV-2 reinfections or vaccine breakthroughs, going forward events of interest may include other pathogens. We may request additional samples including swabs, Synthetic Absorbent Matrix (SAM) strips, oral fluid and serology, participants will be invited to provide these samples which will be optional.

3.9 Reporting of results

The local PCR and serological results will be reported according to the local healthcare organisation's standard occupational health procedures and the methods of communication should be agreed and disseminate by the research team. Requesting tests and accessing results may require an appropriate team (according to the organisations processes this may be the research team and/or occupational health team and/or another designated clinical team) to have access to the individual's electronic health record/pathology system.

Action on positive results: If the PCR screen is positive for SARS-CoV-2, the participant will be advised by the local healthcare organisation and/or NHS Test and Trace (or the relevant contact tracing service in the devolved administrations) of the result and to follow the national guidance for self-isolation and the organisation guidance for return to work, with contact tracing and action as required. If the serology assay is positive, the participant will receive advice from the local healthcare organisation indicating that this does not mean they are immune to reinfection, and that they must continue to adhere to infection control measures at home and work as usual. This may involve the healthcare organisation's research team, Occupational Health, or Laboratory/Virology teams, dependent on local organisational procedures. As previously described, positive specimens/nucleic extracts should be sent for sequencing and viral culture may be attempted by UKHSA for those individuals who are persistently NAAT positive, or have potentially been re-infected.

Results on self-swabs sent to UKHSA for complementary investigation will be provided to the local SIREN research teams, who should then inform the participants.

SIREN 2.0

All NAAT testing will be performed centrally and all results will be communicated to participants directly from the central SIREN team. All SARS-CoV-2 and Influenza (positive/negative) and RSV (positive) results will be uploaded to the Second-Generation Surveillance System (SGSS) or equivalent notification system as under Health Protection Regulations of a notifiable infection. If a positive PCR result is returned participants will be advised to follow local guidance for return to work.

Where new serum samples collected for SIREN 2.0 are tested, participants will receive their serology results directly from the central team. If the SARS-CoV-2 serology assay is positive, the participant will receive advice from the central team in the result report indicating that this does not mean they are immune to reinfection, and that they must continue to adhere to infection control measures at home and work as usual.

3.10 Participant withdrawal

Participants may withdraw at any time, and this is explained in the participant information leaflet. Participants are directed to complete an online survey accessed from the SIREN webpage and advised their withdrawal will only be finalised once they have completed all the questions. On a request to withdraw they will be given the following options:

1. Do you wish to withdraw from SIREN? You will no longer receive follow-up questionnaires or requests for SIREN PCR and serology testing. Yes/No
2. Do you agree for all of your existing survey data and test results to remain in the study? Yes/No
3. Do you agree for us to undertake future testing of your existing samples? Yes/No
4. Do you agree for the study team to continue to link and analyse your results from any future COVID-19 and respiratory pathogen testing outside of SIREN? Yes/No

For the extended follow-up period, participants will be given an explicit option to continue their involvement with SIREN, as either active or passive follow up. Participants will also have the option to end their involvement at the end of their original 12-month follow up period. Among participants opting to take part in extended follow-up, their rights over data and withdrawal for the main study component will be unaffected. If participants wish to withdraw from the extended follow-up they may do so at any time, following the withdrawal process and options outlined above.

Participants will be able to opt out of SIREN activity including data linkage, and communications including study updates and invitations to optional sub-studies and questionnaires on the SIREN portal.

3.11 Storage of materials and additional Laboratory testing

All residual serum, positive swab material, and nucleic acid extract as outlined in the previous sections will be stored at the hospital site and shipped to UKHSA or agreed sequencing laboratories in batches. They may be used for confirmatory testing and serological characterisation, additional respiratory pathogen testing, or for viral genome sequencing and/or culture. An additional swab may be taken if required alongside the primary swab in order to facilitate confirmatory and /or centralised testing pathways. No human DNA genomic investigations will be undertaken.

Additional serological testing: In addition to the baseline cohort serological characterisation described above, SARS-CoV-2 seropositive participants in whom reinfection or with vaccine breakthrough is identified, plus a cohort of matched non-infected seropositive controls, will have their sera further characterised using additional assays and for the presence of neutralising antibody, to provide hypothesis generating data on mechanisms of protective immunity.

Enhanced biochemical and functional serological testing will also be conducted in a subset of participants through associated studies (Appendix 1).

Genomic analysis: All samples positive for SARS-CoV-2 from participants will be sequenced as part of the routine sequencing of NHS residual samples. For participants who have more than one positive PCR test, genomes will be compared where possible to provide evidence to support reinfection or persistent infection. Phylogenetic analysis of SARS-CoV-2 from staff in healthcare organisations, using the study samples and the wider collection of genomes available through the Cloud Infrastructure for Microbial Bioinformatics (CLIMB), will also be undertaken as an exploratory analysis into the diversity and spread of SARS-CoV-2 in healthcare workers.

Viral Culture: Participants with possible re-infection or persistent infection with SARS-CoV-2 will be identified and viral culture may be requested. This may be on residual sample from the swab already taken, but in certain circumstances we may request another swab is taken and sample sent for culture if viral culture is not possible due to the type of sample.

T-cell assays and other studies: Those individuals who are persistently NAAT (nucleic acid amplification test) positive for respiratory pathogens, have potentially been re-infected, or have discordant serology may be contacted by the SIREN Study Team to link into optional associated studies (Appendix 1) e.g. assessing T cell responses, B cell responses or further antibody characteristics

SIREN 2.0:

In addition to the above,which will remain in place, all samples will be stored centrally in a UKHSA approved laboratory and serum samples will be included in the SIREN biobank. Additional assays including for other pathogens may be tested where a relevant scientific question arises as decided by the SIREN Study Management Group.

3.12 Retention

Participants will be involved with SIREN for 12 months, or up to 93 months for those involved in the extended follow-up. During this time, strategies will be employed to ensure sites and participants remain informed, feel valued and their contribution is recognised.

Neither study sites nor participants will be offered an incentive payment for their participation in SIREN. However, the contribution of sites and participants may be recognised with tokens of appreciation including, but not limited to, stickers, badges, lanyards or certificates. Tokens should not be of sufficient financial value to be considered an incentive payment and should not offer benefits to participants that may constitute unreasonably favourable treatment in the workplace. These may be offered by the central UKHSA SIREN team, or by sites at the discretion of the local SIREN study team.

3.13 Ongoing involvement beyond March 2023

The study period for the SIREN study will be extended for a further five years, remaining live until 2028. This will not involve regular PCR, serology or questionnaire follow up throughout the full duration, but linkage to respiratory pathogen test results taken outside the study and access to vaccination records and other key health information registers will be maintained.

During the 5 years of extended follow up as the scientific need arises this cohort may be offered the opportunity to resume testing for a defined period, answer questionnaires, or be invited to join optional sub-studies.

3.14 SIREN 2.0

To support retention of participants sites offering serology will receive additional funding for 2.5 days of research nurse time.

4 Data management

All study documentation will be stored at each site, either in hard copy in a secure environment and/or in electronic copy in an access-limited location on a healthcare organisation server, as decided suitable by the organisation. Use of online platforms for storing study documentation, which should not include PII, could be considered, subject to the healthcare organisation research team providing UKHSA sufficient reassurance that appropriate data security safeguards are in place. Information received by UKHSA as the central study site will be stored securely in access-limited locations on UKHSA servers. Healthcare organisations are required to complete and sign an Organisation Information Document before they are approved to begin recruiting participants into SIREN. This includes a data processing agreement and a data sharing agreement which stipulate requirements around data confidentiality and research governance. UKHSA, as the sponsor, has the right to audit study site compliance with the terms of the agreement.

Study data is as follows:

- Recruitment log, containing personal and demographic information, retained by the healthcare organisation research team, and transfer may be requested by UKHSA SIREN team. Information field to collect are NHS number, name, date of birth, sex, date of enrolment and staff type. The local organisation's SIREN team may also choose to optionally collect ethnicity in order to monitor for quotas or to monitor cohort demographics.
- NHS and Lighthouse laboratory records, identified by personal information – this data (including name, date of birth and postcode) is automatically transmitted to UKHSA as part of the official notification of infection (under Health Protection Regulations) through the Second Generation Surveillance System (SGSS), or appropriate secure alternatives (e.g. CSV files via SFTP) – it includes both molecular and serology results performed by the laboratories. The study will access the routine data generated from the national screening programme in the participant organisations with no additional laboratory requirements. Ideally, these data collected by UKHSA will include the sample type, assay used, cycle threshold (CT) value and optical density as appropriate for each sample tested. For Devolved Administrations (Scotland, Northern Ireland and Wales), laboratory data on healthcare organisation testing may be organised either via: organising reporting from Devolved Administration laboratories through to SGSS, or the Public Health Agency undertaking the laboratory matching for their participants, and then providing linked data back to the UKHSA SIREN team. Details of data processing and approvals for this will be agreed and documented with respective agencies.
- Questionnaire and electronic consent data are entered by the participants and will include participant identifiable information to minimise the burden on research staff, as the sample size has increased. This will be managed through the SIREN information assets, with data received through SnapSurvey (this is a secure web-based system hosted on the UKHSA server) and imported into the SIREN SQL server.
- In addition to the participant questionnaires, vaccination data may be collected via the National Immunology Management System (NIMS). This includes date of vaccination, manufacturer and batch number of the vaccine.
- UKHSA and the Devolved Administration Public Health Agencies may access centrally held information to find identifiers (e.g. NHS number) and may also link centrally held health and care data to the data collected in SIREN.
- Viral genomic data is identified by a unique sequence identifier (pseudonymised with the link to personal information held by UKHSA under existing surveillance protocol). Genomic data for SIREN is held within UKHSA data assets and within the UKHSA network.
- Viral culture results and the results of UKHSA serology testing will be available to the UKHSA SIREN team through linkage with the UKHSA MOLIS data asset.
- Where centralised laboratories are utilised for molecular or serological testing, data will be transferred securely to the UKHSA SIREN team.

All source data will be securely transferred to UKHSA with the identifiers described above. Data will be received into UKHSA from the survey (a secure and encrypted web survey hosted on a UKHSA server), via secure email (recruitment log), through UKHSA's established data assets Second Generation Surveillance System (SGSS; laboratory data), MOLIS, or through end-to-end encrypted bespoke arrangements (e.g. genomic data). The person identifiable information is required to run the follow-up survey (dependent on having names and contact number/email), and for UKHSA to undertake secure data linkage across the data assets as well as drawing in data from the Lighthouse laboratories.

The SIREN database will be a SQL database (with MS Access front-end) on a secure server at UKHSA with access restricted to named authorised staff within the UKHSA SIREN team. Participant Identifiable data including full name, email address and mobile number is stored in a separate database table to the main parent table. This will reduce viewing frequency of this data as the main parent table will be regularly accessed by the study team to generate reports. All analysis will be performed by study personnel on the pseudonymised linked datasets.

Access to Data: Direct access will be granted to authorised representatives from the Sponsor for monitoring and/or audit of the study to ensure compliance with regulations. Access to study data and responsibility for undertaking data management and analysis will be undertaken by named authorised staff within the UKHSA SIREN team. All members of the SIREN team involved in data linkage have been trained in handling data according to Caldicott guidelines and Section 60 of the Health and Social Care Act. All researchers are aware of the Data Protection Act 2018, and the need to maintain absolute confidentiality.

Data Recording and Record Keeping: The data will be securely held at the National Infection Service, UKHSA. Data collection, storage and use will be consistent with the procedures described in the NHS Information Governance Toolkit. All databases will be encrypted and appropriately access-restricted.

Data storage: Electronic data will be stored on UKHSA secure servers and will remain active for the duration of the study. Participants' identifiable data will then be removed and the data will be archived within the platform to be retained for a period of 5 years.

Records retention policy: The continued secure storage of participant data in all SIREN information assets will be subject to a regular (quarterly) review by the SIREN Study Management Group. Decisions on whether to continue to retain this data and the justification for this will be documented in the SIREN study Decision Log.

5. Analysis plan

5.1 Overall Analysis

All enrolled participants will be included in analyses, which will account for clustering by research site. Analyses will be conducted regularly to inform the UK's response to the COVID-19 pandemic.

Results will be available to all organisations involved in the research. The study follow-up period will end by default 12 months following the enrolment of the last participant, but by consensus of the study management group and funder may be extended or terminated sooner if findings are sufficient.

There are no formal stopping rules for futility, utility or lack of power. The final decision to terminate the study will be made by UKHSA and Department for Health and Social Care.

Estimates of both cumulative incidence and incidence density in the seropositive and seronegative cohorts will be obtained using mixed effects models assuming counts of PCR positive have a negative binomial distribution, a log link function, and the natural logarithm of the total number of subjects or the total follow-up time use as an offset, respectively. Inclusion of a binary predictor indicating the sero-status of the cohort into this model will provide estimates of the incidence rate ratio. Sites will be incorporated as a random intercept to account for unmeasured, shared, site level factors. To account for a non-constant force of infection, calendar month will be incorporated as an additional random effect. An assessment of the role of factors such as age, gender, ethnicity in immunity will be explored by inclusion of interactions within the model between each and serological status.

While the above analytical approaches provide a “classical” person-years approach to prospective cohort analysis and provide familiar measures of association, it may be inadequate to assessment of immunity provided by seroconversion. As it is expected that seropositivity is likely to confer a degree of short to median term protection for a SARS-CoV-2 infection, multi-state and parametric cure rate models incorporating frailty will be employed. These “survival” type of models provide a more detailed assessment of factors associated with both short term and longer-term protection from infection, and how immunity may wane over time. Both mixture, explicitly assuming an immune and non-immune group and non-mixture “cure rate” models will be assessed using information criterion to choose which provides a better fit to the observed data. Bayesian approaches to cure rate models with frailty as describe by deSouza[13] will be employed.

Multi state models explicitly allowing those within an “immune” state to flow into a “susceptible” state as antibodies wane will also be employed. This framework can allow subjects to move from seronegative (susceptible) to seropositive (immune) when infected during the study period. An additional absorbing state will be used for those infected that died. It is also possible to introduce “misclassification” of state into the multi state model, providing an estimate of sensitivity to account for imperfect serological tests. Approaches like those proposed by Jackson[14] will be employed.

5.2 Analysis for vaccine effectiveness (VE)

Survival analysis with time-varying covariates will be used to estimate the hazard ratio in vaccinated compared to unvaccinated SIREN participants with VE = 1 – HR. A nested test negative case-control analysis will also be done with those swabbed but negative as the controls. If more than one vaccine is used vaccine effectiveness will be stratified by vaccine manufacturer.

Vaccine effectiveness will also be stratified by baseline positivity (either PCR or antibody), age group (<50, >=50) and time since vaccination (three-month intervals and as a spline). Interaction with sex, ethnicity and risk group will be tested and, if significant, vaccine effectiveness will be stratified by these factors.

If the vaccine is rolled out over a very short period to HCWs with very high coverage then the unvaccinated group will be small and probably an unusual subset. Even if coverage is not high those that do not get vaccinated when it is highly recommended may be different in ways that could lead to confounding. For example, those previously infected may not see the need for vaccination, or those not regularly working on site might miss vaccination. Those that perceive themselves as low risk of severe disease or with less patient contact may also be less likely to get the vaccine. Those not getting vaccinated may also be more likely to be those not providing regular swabs or blood samples. It will therefore be important to compare the vaccinated and unvaccinated cohorts to identify these potential biases. Using only those completing regular follow-up may help reduce such biases.

If coverage is very high and rapid then instead of vaccine effectiveness assessment it may be possible to do an impact assessment using a controlled interrupted time series approach in which COVID-19 incidence is compared over time in the HCW population to the general population (using external data) or between sites if vaccine introduction varies sufficiently by site. This can be done using Poisson or negative binomial regression.

Procedure for Accounting for Missing, Unused, and Spurious Data: Analyses will be restricted to cases with antibody and PCR tests. The PCR test for virus is being used as a diagnostic test and hence has high performance. Sufficient sera will be obtained to re-run the immunological assays in case of initial assay failure. For similar reasons we do not anticipate that spurious data will be obtained.

Procedures for Reporting any Deviation(s) from the Original Statistical Plan: Deviations from the original statistical plan or the statistical analysis plan will be described and justified in the analysis reports.

6. Confidentiality and information governance

The data management plan (Section 4) demonstrates that UKHSA and the Public Health bodies in the Devolved Administrations will need to be able to extract data from multiple healthcare organisations, including staff moving between organisations, national testing laboratories undertaking clinical diagnostic tests when staff are unwell, and potentially national sequencing consortium laboratories. Data linkage based on a single study identifier is neither practical nor robust in such circumstances. The suggested process for handling identifying data in a secure and appropriate way is therefore as follows:

- On enrolment, participants will be given a unique study number (three letter/number combination (five for Scottish Health Boards and Northern Irish Health and Social Care Trusts) identifying the healthcare organisation, plus five digits identifying the participant)
- The healthcare organisation will compile a participant database (Recruitment Log) containing the name, job role, date of birth, sex NHS number (or equivalent unique identifier in Devolved Administrations), and study number, passcode of each enrolled participant. They may need to collect other personal information in order to monitor quotas, request tests and contact participants appropriately. They may also develop local tools to assist managing patient, sample and data flows and returning results; if they do so this should be stored according to information governance requirements.
- The local healthcare organisation will retain these data for local study management and will transfer a copy of the database securely to UKHSA using appropriate encryption if requested and will be stored, password protected, in a limited-access environment on a UKHSA server.
- UKHSA and the Public Health Organisations in the Devolved Administrations will use the participant's personal information to retrieve their test results from the national surveillance systems as described in the data management plan. They may also use personal information to request the residual samples for additional processing in the sub studies and to link to identifiers and health and care data held centrally.
- Positive swabs from the study will be routinely sequenced under the arrangement between the NHS and sequencing laboratories. In order to retrieve the genomic data, UKHSA and Public Health Organisations in the Devolved Administrations may use participant's identifying information to search the sequencing results databases, which are within the public health organisations, to retrieve the unique sequence ID. The unique sequence ID allows the public health organisations to retrieve the genomic data for that sample from the Cloud Infrastructure for Microbial Bioinformatics (CLIMB, which holds a pseudonymised genome collection on a non-UKHSA server).

- The SIREN SQL database will receive and manage data from the above data sources. This will include participant identifiable information, as required for testing linkage and communication of follow-up survey links to the participants. The continued retention of participant identifying information will be subject to regular review by the Study Management Group, as per the Records Retention policy.
- If a participant moves to another healthcare organisation, it will be the responsibility of the original enrolling organisation to organise the participant's transfer to a new SIREN participating organisation, and to notify the UKHSA SIREN team of this change. Any communication involving PII or study number should be sent by secure email, and guidance is provided to all healthcare organisations on this.
- If, following review of results, the study team deem it necessary for result interpretation, access to past results or clinical management of a participant, we may contact sites to discuss individual participant results either by email or on the telephone; the discussion would be with the most appropriate individual at the site e.g. research team, occupational health, microbiology consultant. We may request access to pseudonymised results which are usually held by Occupational Health.

UKHSA has undertaken some seroprevalence surveys (LondonCOVID and ESCAPE); these studies have been/are being terminated and individuals who are eligible are being offered the

opportunity to transition into SIREN. As the data from these studies would be extremely useful to SIREN, we are keen to access these results, however the protocol/consent for these surveys do not include the use of patient identifiable information by UKHSA or the use of results in other studies. Therefore, we have included a supplementary consent form for these participants which the local research teams will retain locally. The use of data from these surveys is optional and refusing consent would not impact and individual from being able to participate in SIREN.

7. Quality assurance and research governance

The study may be monitored or audited in accordance with the study protocol and standard operating procedures, GCP and relevant regulations.**Risk assessment:** No formal risk assessment is required. The study involves recruiting individuals without symptoms who will be asked to give full informed consent to have swabs taken or self-swab their nose or nose and throat, and provide a blood sample taken by a study research nurse, the hospital phlebotomy department or another individual judged to have been appropriately trained. Further participation in follow up visits to collect the same samples is based on consent of the participant. The main burden of participating in the study is the time taken for the study visits (which will be as far as possible at the same time as routine organisation mandated screening if it is in place) or the potential for minimal bruising from blood sampling though this is unlikely with experienced staff taking the samples. As to the potential impact on work if a sample is returned as positive; while a positive result may necessitate self-isolation, the impact of identifying these asymptomatically positive cases would be regarded as a positive outcome from the perspective of reduced of risk to patients, other staff and social contacts. There is minimal risk of harm to any patient from participating since it does not include any therapeutic intervention. The diagnostic test for the presence of virus from the nose or nose and throat swab will be conducted by an accredited laboratory (or those with satisfactory verification documents) and will be returned to the participant’s research nurse and participant through standard hospital practice.

Study monitoring: As there are minimal risks posed to patients by this observational and non-interventional study, formal study monitoring is not planned. However, sites are expected to adhere to the study protocol, and conduct the study in line with the principles of GCP to ensure best practice. The study team will liaise with sites regularly to ensure samples are being sent in a timely fashion.

Safety reporting: There are no interventions in this study, and the only procedures are a standard blood draw performed by a healthcare professional, and a participant swab by a trained professional or self-swab using a methodology that is being used widely across the country. Therefore, there is minimal safety risk to participants and safety reporting is not applicable.

Study committees: Oversight will be provided by a study management group including the investigators named above, representatives of collaborating and participating organisations as appropriate, and chaired by the Chief Investigator.

Protocol deviations: A deviation is a departure from the approved study protocol or other study document or process, or any applicable regulatory requirement. Any deviations from protocol will be documented by the team delivering SIREN in each organisation in a protocol deviation spreadsheet, which will be stored securely and sent to UKHSA securely on a monthly basis.
Protocol deviations which we would expect to be recorded and reported to the Sponsor monthly up to March 2023 include (but are not limited to):

- Participants who miss more than eight consecutive visits.
- Participants who did not have the correct samples undertaken at any given visit
- Where samples are incorrectly discarded or not tested as per protocol

For SIREN 2.0 protocol deviations we would expect to be recorded and reported to the Sponsor monthly include (but not limited to):

- Participants who miss more than 2 consecutive bleeds.

Serious breaches: A serious breach is a breach of the protocol or of the conditions or principles of GCP which is likely to affect to a significant degree –

(a) the safety or physical or mental integrity of the trial subjects; or

(b) the scientific value of the research.

In the event that a serious breach is suspected the Sponsor must be contacted within 1 working day. In collaboration with the Chief Investigator, the serious breach will be reviewed by the Sponsor and, if appropriate, the Sponsor will report it to the approving REC committee and the relevant local healthcare organisation within seven calendar days. A serious breach would include a data breach. The organisation (both local and relevant public health body) will be expected to complete a review document which examines and circumstances, response, root cause and mitigation actions, and return this document to the Sponsor.

Appendix 1: Collaborators

The SIREN Study has collaborations with multiple academic partners to maximise the utility of the data and samples collected as part of the SIREN study. As new research is developed over time the list of collaborators will change. All collaborators will adhere to the data sharing agreements outlined in the main protocol and data sharing agreements.

Below is the current list of active research collaborators:

**Institution**	**Named partner**
British Society for Immunology	Jennie Evans<br><br>Erika Aquino
The Francis Crick Institute	Rupert Beale<br>Edward Carr
Imperial College London	Wendy Barclay
Newcastle University	Christopher Duncan
University of Birmingham	Alex Richter
University of Cambridge	Helen Baxendale<br>Javier Castillo-Oliveras Pallardo<br>Jonathan Heeney<br>Wilhelm Schwaeble<br>James Thaventhiran
University of Edinburgh	Kenneth Baillie

University of Liverpool	Lance Turtle
University of Oxford	Susannah Dunachie<br>Paul Klenermann
University of Sheffield	Thushantha de Silva
Wellcome Sanger Institute	Ewan Harrison
World Influenza Centre	Ruth Harvey

Appendix 2: Multiplex Respiratory Pathogens Sub-study 2022-2023

Summary:

Over the course of the COVID-19 pandemic it has become increasingly clear that COVID-19 will become endemic in the population. This therefore means that it will be present alongside other respiratory pathogens that cause significant morbidity and mortality. The government has set out guidance for living with COVID-19, removing the requirement for social distancing measures and widespread use of face coverings, both of which likely reduced influenza transmission and therefore the incidence of influenza cases over the last two years. Australia had the most cases of influenza in the last 5 years in 2022 due to a reduction in social distancing and universal masking and it is therefore likely that there will be high numbers of cases in the United Kingdom this winter[13]. SIREN ideally placed to study the impact of influenza, and other respiratory pathogens, with a cohort of highly motivated HCW who are used to regular testing and an established clinical and laboratory infrastructure to process these samples. This gives SIREN the unique opportunity to test for other respiratory pathogens as well as SARS-CoV-2 to investigate the incidence and impact on the HCW population.

It is vital for policy makers to understand the impact of these respiratory pathogens over winter in order to implement strategies ensuring patient and healthcare worker safety. The SIREN study will therefore test for influenza A, influenza B and other respiratory pathogens where available, in addition to SARS-CoV-2.

Throughout winter 2022-23 SIREN will therefore undertake a sub-study within our cohort. Where available, SIREN sites will process PCR samples on their local multiplex assay including, but not restricted to, influenza A/Band SARS-CoV-2. Other respiratory pathogens will be tested according to the specific multiplex assay used. The interaction between SARS-CoV-2 and other respiratory pathogens will become increasingly important as we progress to endemicity. This sub study will investigate the effects of a variety of respiratory infections in HCWs, particularly COVID-19 and influenza which will be vital for future workforce planning.

It has become increasingly apparent that COVID-19 is not seasonal in the same way that Influenza is and the RSV seasons rates over the last year have been unusual, with levels high earlier and persisting longer. Therefore, to better understand this, the sub-study plans to extend the postal pathway participants beyond March 2023. It is vital that there is good understanding of how COVID-19 interacts with other respiratory pathogens throughout the year.

Aims and Objectives

As the main SIREN protocol, with the addition of the following sub-study objectives:

Primary objectives:

1. Incidence of influenza infection in healthcare workers
2. Influenza vaccine effectiveness in healthcare workers

Secondary objectives:

1. Incidence of asymptomatic influenza
2. Incidence of co-infection (e.g. influenza and SARS-CoV-2)
3. Influenza vaccine effectiveness by vaccine type
4. Symptom variation between respiratory pathogens
5. Number of sick days caused by influenza and other confirmed respiratory pathogens
6. Serological response to influenza vaccination over time

Methods

The sub-study methodology will mirror that of the main SIREN study, with the addition of use of multiplex assays for influenza and any other respiratory pathogens available, and where available increasing serology sampling to monthly as seen in figure 1.

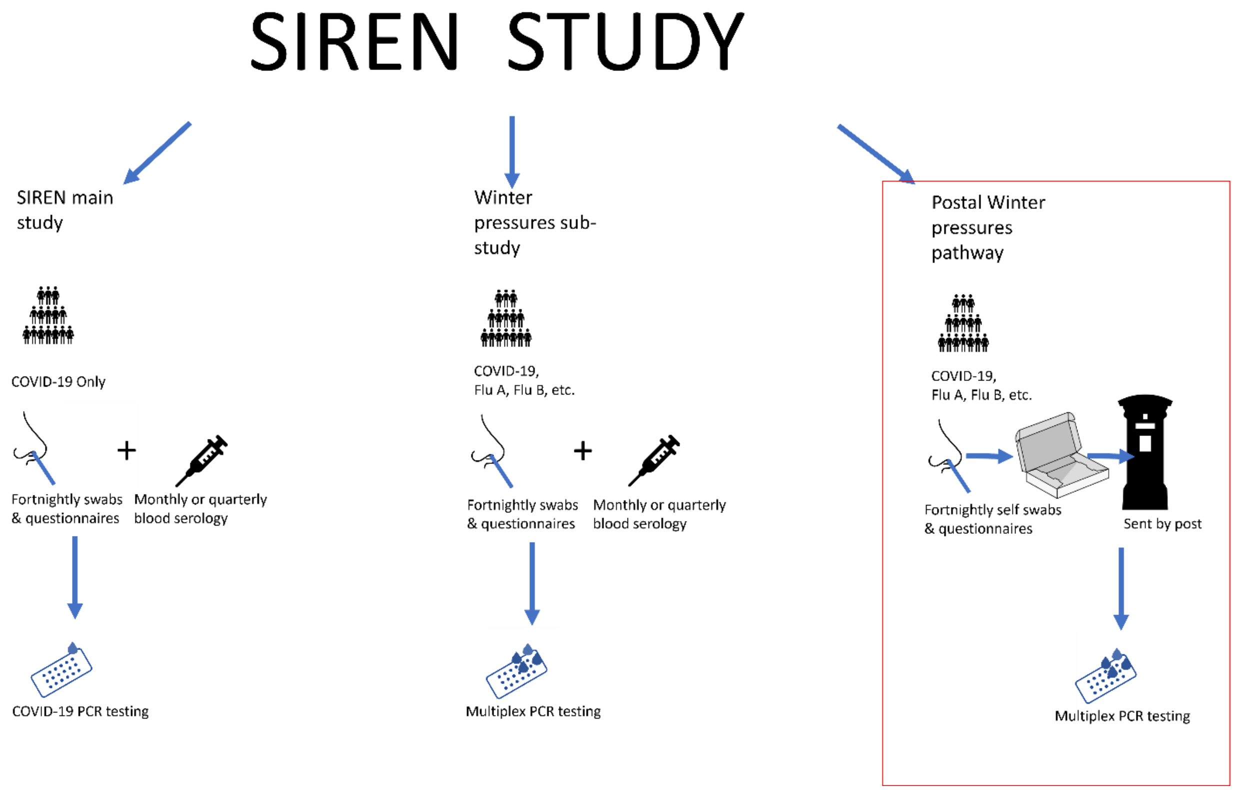

Figure 1: Diagram illustrating the SIREN main study and winter pressures sub-study protocols including a postal pathway

Study sites:

A subset of sites already participating in the main SIREN study that are able to offer multiplex PCR will switch to this in Winter 2022-23 allowing detection of influenza A, influenza B and any other respiratory pathogens on the local sites' panel. Sites unable to offer multiplex will continue offering dedicated SARS-CoV-2 testing. If sites are unable to continue to offer the requirements of the study described above UKHSA may offer a centralised system to facilitate participant retention.

If participants wish to continue in the SIREN study but sites are unable to offer SIREN testing, participants will be offered a centralised postal self-swab pathway to enable them to continue in the study. It has become clear via feedback from participants that some participants are keen to continue but their sites are unable to offer regular testing. These participants will follow the "postal pathway".

Identification / Recruitment of participants:

Of the SIREN sites that confirm participation in the Winter Pressures sub-study, all participants at that site will have to consent to collection of their samples for the multiplex testing.

This information will be provided to all participants at the sites participating in the Sub-study in advance of their next appointment.

To relieve the burden both on research staff and the time commitments of the NHS staff participants', attendance at the following study visit will be regarded as 'inferred consent'. Participants will have detailed information regarding the sub-study provided to them well in advance of their next appointment with the opportunity to carefully consider their participation in the sub-study. Participants will also be able to ask questions from the research team at their next SIREN appointment if required. The decision not to ask participants to undertake formal written consent is following careful consideration and consultation with DHSC, UKHSA, NIHR, local clinical research networks (CRNs), HRA and has the support from the SIREN participant involvement panel who all believed it was the best approach.

Participants who do not wish to participate in the Winter Pressures Sub-study will be informed that they are able to transfer to sites not offering the sub-study or can withdraw from the study.

Previous participants from sites joining the sub-study who have completed their active follow up but remain study participants (i.e. who have not formally withdrawn) will be invited to join the sub-study. Participants will be invited using a new SnapSurvey consent form which will be distributed via email/text depending on participants' preferences, as with other communication. .

Participants who are joining the postal pathway will be offered a new consent form via SnapSurvey and receive a participant information leaflet outlining the process. They will need to provide an address to receive their packs and will be informed that this information will be shared with a UKHSA approved third party to deliver the packs, using an approved company that is contracted by UKHSA.

Active SIREN sites who would like to join the winter pressures sub-study, but where their laboratory is unable to support this, may also provide samples for centralised testing at UKHSA or third party laboratories. These samples will either be the residual sample once SARS-CoV-2 testing is completed locally, or by taking two swabs from each participant. One swab will be tested locally for SARS-CoV-2 as per the current SIREN pathway, the other packaged and sent to a central laboratory for multiplex testing. The results from the central laboratory may not be in real time and participants may receive the results at any point up until the end of the study. Discordant SARS-CoV-2 results (from local laboratories and central laboratories) will be audited and assessed according to antibody results and CT values, with standard operating procedure documents to determine any actions required. Sites who are offering the two-swab option may allow participants to opt in or out of the winter pressures pathway, if this is logistically feasible. Participants who do not wish to join the winter pressures pathway can discuss with sites whether they can continue with the original SIREN pathway or if they should withdraw from the study altogether. A specific participant information leaflet will be provided to participants of these sites. By allowing a second swab to be taken this will be considered implicit consent to the winter pressures sub-study.-

Participants who are joining the postal pathway will be offered a new consent form via SnapSurvey and receive a participant information leaflet outlining the process. They will need to provide an

address to receive their packs and will be informed that this information will be shared with a UKHSA approved third party to deliver the packs, using an approved company that is contracted by UKHSA.

Data collection and measurements:

Molecular screening: Sites in the sub-study will offer multiplex assays and this will be undertaken according to the local organisation procedure for screening healthcare workers or through a protocol set up specifically for this study. Screening will be performed at regular intervals from 1 to 4 weeks (as specified by the UKHSA SIREN study team). It may be a nose swab or a nose and throat swab and may be undertaken by self-swabbing or administered by a trained member of staff depending on the local organisational preference. Participants who are self-swabbing may return samples to a central site by post. The swab will be tested for SARS-CoV-2, Influenza A and Influenza B RNA as well as any RNA or DNA from any other respiratory pathogen available on the panel in an accredited laboratory using a validated nucleic-acid amplification test (NAAT), such as RT-PCR. Residual specimen and any available nucleic RNA extracts should be stored at $-80^{O}C$. Where available local SIREN sites may offer influenza typing as per local protocols and further genotyping / whole genome sequencing may be requested on pathogens where available.

Serology: Where possible serology samples should be taken monthly rather than quarterly

Questionnaire: Additional questions are added but these will be asked to both those in the Sub-study and in the main study and are covered in the main protocol.

Postal Pathway: Participants will follow a mirrored methodology to the main SIREN study, however participants will have less testing and some will be performed from home via self-collection. Participants will continue the same fortnightly questionnaires and will continue to swab every fortnight. Participants will receive self-swab kits by post (as has been conducted in many NHS sites throughout the pandemic) and will package and mail the kits back to a central laboratory or laboratories. Testing will be co-ordinated by the central SIREN team and performed by UKHSA approved central laboratories.

Some swabs may contain guanidine, an inactivating transport medium that enables high throughput testing in certain SIREN sites. This is in standard use in many other kits including routine use for home sexually transmitted infection kits in the UK. This is an irritant to the skin and eyes, and a risk assessment has been conducted separately within SIREN for this and hazard instructions will be issued to participants within the instructions for users. Warnings and mitigating steps to avoid contact will be advised. This was discussed with the participant information panel who felt that it was acceptable.

Whilst fortnightly swabbing is optimal to achieve the objectives of this study, participants will be encouraged to provide samples according to what is most practical. Therefore these participants

will not have a designated testing window and missed swabs will not be considered a protocol deviation. Participants will only continue with regular serology if it is convenient and practicable and enhanced serology is not required for events of interest.

Beyond March 2023 participants will be offered to continue on this pathway with regular self-swabbing, questionnaires and no serology. As more sites close following March 2023 other participants may be invited to join this pathway. This will enable surveillance of COVID-19, RSV and Influenza beyond the winter months.

Reporting of Results:

Action on positive PCR results for other respiratory pathogens will be guided by current guidance and protocols. Where no national guidance is available, this will be the responsibility of local teams to manage as per their local policies with advice from the UKHSA SIREN team.

Participants in the postal pathway will not receive results in real-time, however, notifiable infections will be notified to public health agencies via SGSS. Participants will be informed of their results at the end of the study and will be informed of this as part of the consent process.

Additional Laboratory Testing:

Additional testing will be the same as per the main protocol, but may be performed for influenza and other respiratory pathogens, specifically:

Serology: Further serological investigation relating to other respiratory pathogens will be performed in certain cohorts such as, influenza vaccine breakthrough or persistent infection or other groups identified by the SIREN consortium.

Genomic Analysis: Positive samples for influenza may also be typed according to local protocols. Additional genetic or phylogenetic analysis of influenza may also be performed in other groups identified by the SIREN consortium

Funding

Funding has been secured within the current SIREN budget. The majority of NHS England provided assays have no difference in cost between monoplex and multiplex and reimbursement follows the same model as the main study. For any sites using systems that are not funded centrally there is funding from within the UKHSA budget to reimburse sites directly.

Appendix 3: SIREN Qualitative Sub-Study and Evaluation

Summary:
Due to the nature of running a clinical trial during a pandemic the SIREN study has needed to be agile and adapt to stay relevant. A major method of ensuring this is feedback acquired from sites, participants and the central team. Through regular feedback themes emerge that are of interest when planning future iterations of the SIREN study but also to attitudes towards COVID-19, vaccination and the impact of winter respiratory pathogens on the workforce. In order to gain a better understanding a qualitative research arm has been formed as part of the SIREN study.

Aims and Objectives:
Aims:

- To build better understanding of the barriers and levers to site participation in the study and to identify lessons for future manifestations of the study
- To consider the impact that SIREN data has on sites and participants
- To explore specific themes that have emerged from SIREN data collection including but not limited to: staff experience, vaccination and attitudes towards COVID-19 and other respiratory pathogens

Methods:
The evaluation will utilise standard qualitative research tools. This will include a combination of focus groups and semi-structured interviews. To ensure that there is a broad range of opinions, participants of the focus groups will be recruited from numerous areas, including participants, sites, central SIREN team and staff at sites who are not directly involved in the SIREN study, but potentially impacted by the results. Staff not directly involved with SIREN could include infection control teams, occupational health, hospital management teams who could all be impacted by the SIREN study testing their staff regularly.

Focus groups will be conducted either in person or online depending on what is possible to ensure that there is representation across the UK. They will be run by researchers with experience in qualitative research and where possible will have two researchers facilitating each group. The researchers will follow a topic guide and, where specific themes are to be explored will have structured questions.

Participants will be selected to ensure as representative a cohort as possible, to enable this, a combination of generalised and targeted recruitment may be employed. Participants will be offered an honorarium in line with NIHR guidance. Over-recruitment will be pursued to counteract attrition.

Thematic analysis will be conducted by the researchers with initial theming performed by one and then cross referenced by another. This will then be streamlined into themes and written into a report. These reports will be used internally to shape the SIREN study going forward and where relevant public health themes emerge will be passed to Governmental organisations and published in peer reviewed journals.

Funding:
Funding for the study comes from the current SIREN study budget and an HDR-UK grant (HDRUK2022.0322) awarded to fund the appointment of external researchers with experience

with qualitative research. The main cost is the staff time for conducting and analysing the qualitative research, however, an honorarium will be paid for participants at the rate recommended by the NIHR[16].

Appendix 4: Amendment History

Amendment No.	Protocol Version No.	Date issued	Author(s) of changes	Details of Changes made
1	2	20052020	Susan Hopkins	Removed Appendix 1 including PIL, Consent and Questionnaires<br><br>Added table of contents
2	3.1	08062020	Susan Hopkins	Modified Consent to be electronic to reduce data burden.<br><br>Increased sample size to determine whether there is immunity at much shorter time intervals to provide UK government with evidence of immunity or not at much faster intervals.<br><br>Modified sample storage to reflect the larger sample size<br><br>Added that PCR swab samples could form part of screening protocol for Trust or specific to this study and taken by nose and throat swab or nose self sampling.<br><br>Removed RT-PCR and changed to PCR to allow other local platforms to be used.

3	4.1	16082020	Susan Hopkins	**Eligibility for sites extended to primary care/CCG and the independent sector.**<br><br>**Eligibility for sites from Devolved Administrations added**<br><br>**Eligibility of PIC (Participant identification centres) sites**<br><br>**Further details on inclusion/exclusion criteria.**<br><br>**Further details on electronic consent.**<br><br>**Action on positive results expanded**<br><br>**Removal of mention of NHS policy for routine swab testing in all hospitals in England.**<br><br>**Removal of plan to provide quotas initially.**<br><br>**Clarification of recruitment period to end 30/9/2020 for all sites.**<br><br>**The enrolment log to continue to be maintained by local organisation but transferred to UKHSA only on request.**<br><br>**Change from multiple aliquots of follow-up serum to one full 2ml serum.**<br><br>**Clarification of which follow-up serum samples need stored and sent to UKHSA.**<br><br>**Viral culture detail added.**<br><br>**Option of requirement to obtain additional swab for further testing.**<br><br>**Further description of who can take blood/swab samples – can be done by peer to peer sampling as long as**

				**approved by organisation guidance and individuals appropriately trained.**<br><br>**Added using individual's results from previous studies (including additional consent procedures for those in sero-prevalence surveys).**<br><br>**Options for publicity materials added**<br><br>**Description for paper consent process if website is down.**<br><br>**Further description of staff who are eligible**<br><br>**Protocol Deviations further defined with reporting mechanisms for deviations and breaches**<br><br>**Explicitly stated that local teams will need access to participants electronic record.**<br><br>**Further information about data collection and storage**<br><br>**Records retention policy added**

**4**	**5.0**	**20112020**	**Susan Hopkins**	**Date of recruitment extended to 31/03/21.**<br><br>**SARS-COV2 updated to SARS-CoV-2**<br><br>**Reference to wider immunology studies added**<br><br>**Appendix of wider immunology collaboration studies added**<br><br>**Maximum volume of blood samples increased to facilitate sample collection for wider immunology studies**<br><br>**Data Protection Act 1998 updated to 2018 to include GDPR legislation**<br><br>**Updated protocol deviations to reduce burden on sites**
**5**	**5.1**	**23122020**	**Susan Hopkins**	**Updates to reflect the introduction of a COVID-19 vaccine among healthcare workers, including; updated background, two aims added, updated analysis plan and the expansion of study data to include data from the National Immunology Management System (NIMS).**<br><br>**Updated additional serology testing and assays to include analysis of B cells.**<br><br>**Additional collaboration study included in Appendix 1.**
**6**	**5.2**	**07012021**	**Susan Hopkins**	**Leaflet at vaccination added as an additional means of recruitment**

**7**	**6.0**	**28052021**	**Susan Hopkins**	**Updates to reflect the addition of an extended follow up period including; updated summary, background, primary aim, enrolment and consent, data collection and methods and withdrawal procedures.**
**8**	**6.1**	**17062021**	**Susan Hopkins**	**Details of optional antibody testing added as an additional testing method, subject to local procedures.**<br><br>**Appendix name amended to ‘Wider Immunological Studies’ for clarity**<br><br>**Frequency of missed visits increased from three to eight missed visits to qualify as a protocol deviation.**
**10**	**6.2**	**11/08/2021**	**Susan Hopkins**	**Addition of paragraph outlining retention strategy and enabling participants to receive tokens of appreciation**
**14**	**6.3**	**23/12/2021**	**Susan Hopkins**	**Updating branding to UKHSA**<br><br>**Updating laboratory process in line with Laboratory manual v.4.1**<br><br>**Inclusion of paragraph 3.8.3 outlining process for follow up of Events of Interest.**

**15**	**6.4**	**13/01/2022**	**Susan Hopkins**	**Section 3.8.1 SARS-CoV-2 molecular screening**<br><br>**Additional sentence added in line with national guidance**<br><br>**Where lateral flow device (LFD) tests are used, all positive LFD tests should be confirmed using a validated NAAT such as RT-PCR.**
**16**	**7.0**	**23/6/2022**	**Susan Hopkins**	**Updates to reflect the addition of an extended follow up period including; updated summary and background**
**17**	**7.1**	**08/08/2022**	**Susan Hopkins**	**Updates to reflect the addition of questions to the follow up survey relating to LFD results and exposures to COVID-19 at work and in the community**
**18**	**7.2**	**12/08/2022**	**Susan Hopkins**	**Additional questionnaire asking questions on symptom duration and severity of COVID-19 infection to all participants who had a history of COVID-19 infection**
**19**	**7.3**	**15/08/2022**	**Susan Hopkins**	**Additional questionnaire asking if participants had tested positive by LFD since April 2022 and if this was confirmed by PCR**
**20**	**7.4**	**06/10/2022**	**Susan Hopkins**	**Additional pathway to allow postal swab kits to be sent to participants who test positive by LFD and an additional questionnaire to further understand the participants previous clinical history**
**21**	**8.0**	**14/11/2022**	**Susan Hopkins**	**Additional appendix 2 outlining the SIREN Winter Pressures sub-study investigating other respiratory pathogens in addition to SARS-CoV-2**
**22**	**9.0**	**01/12/2022**	**Susan Hopkins**	**Addition of a postal kit pathway to the SIREN Winter Pressures sub-study to ensure sufficient participants for power**

**23**	**9.0**	**01/12/2022**	**Susan Hopkins**	**Addition of a study poster, adjustment of postal leaflet for self swabbing**
**24**	**9.1**	**03/01/2023**	**Susan Hopkins**	**Adjustments for sites offering Pathway B to allow two swabs to be taken at each visit**
**25**	**10.0**	**17/02/2023**	**Susan Hopkins**	**Additional appendix 3 outlining a qualitative research arm to evaluate the SIREN study**
**26**	**10.1**	**28/03/2023**	**Susan Hopkins**	**SIREN is extending into another year and this will include provision to continue passive data collection for all participants for 5 years as well as to continue the postal pathway for some participants to test over the Spring/ Summer.**
**27**	**10.1**	**17/04/2023**	**Susan Hopkins**	**Enables the restarting of postal pathway participants testing and surveys and provides a clearer updated process to reflect a change in specimen labelling requirements.**
**28**	**10.1**	**31/05/2023**	**Susan Hopkins**	**An extension to the PITCH substudy (nested within SIREN), updated to reflect the evolving pandemic, specifically mentioning RSV and flu and potential to study other pathogens.**
**29**	**10.1**	**05/06/2023**	**Susan Hopkins**	**Minor changes to key study documents to improve comprehension and accessibility following feedback from users.**
**30**	**10.1**	**21/06/2023**	**Susan Hopkins**	**Minor changes to key study documents to reflect an extension to the testing schedule. This supports a minor change for the postal pathway, specifically extending testing until March 2024.**

**31**	**11.0**	**14/08/2023**	**Susan Hopkins**	**SIREN 2.0**<br><br>**Protocol updated to bring all participants onto the same testing strategy. This includes regularly testing for influenza A/B and RSV as well as SARS-CoV-2.**

References

1. Zhu N, Zhang D, Wang W, et al. A Novel Coronavirus from Patients with Pneumonia in China, 2019. *N Engl J Med* 2020;382(8):727-33.
2. World Health Organisation. WHO announces COVID-19 outbreak a pandemic. Available from: http://www.euro.who.int/en/health-topics/health-emergencies/coronavirus-covid-19/news/news/2020/3/who-announces-covid-19-outbreak-a-pandemic. WHO: Geneva, 2020 (accessed 11 May 2020).
3. World Health Organisation. Coronavirus disease (COVID-19) Situation Dashboard. Available from: https://covid19.who.int/. WHO: Geneva, 2020 (accessed 11 May 2020).
4. Public Health England. Guidance and standard operating procedure COVID-19 virus testing in NHS laboratories. Available from: https://www.england.nhs.uk/coronavirus/wp-content/uploads/sites/52/2020/03/guidance-and-sop-covid-19-virus-testing-in-nhs-laboratories-v1.pdf. UKHSA: London, 2020 (accessed 11 May 2020).
5. Konrad R, Eberle U, Dangel A, et al. Rapid establishment of laboratory diagnostics for the novel coronavirus SARS-CoV-2 in Bavaria, Germany, February 2020. *Euro Surveill* 2020;25:9.
6. To KK, Tsang OT, Leung WS, et al. Temporal profiles of viral load in posterior oropharyngeal saliva samples and serum antibody responses during infection by SARS-CoV-2: an observational cohort study. *Lancet Infect Dis* 2020;20(5):565-574.
7. Wikramaratna P, Paton RS, Ghafari M, Lourenco J. Estimating false-negative detection rate of SARS-CoV-2 by RT-PCR. *MedRvix* 2020; https://www.medrxiv.org/content/10.1101/2020.04.05.20053355v2**.**
8. Li Z, Yi Y, Luo X, et al. Development and clinical application of a rapid IgM-IgG combined antibody test for SARS-CoV-2 infection diagnosis. *J Med Virol* 2020;doi:10.1002/jmv.25727
9. Hamre D, Beem M: Virologic studies of acute respiratory disease in young adults. V. Coronavirus 229E infections during six years of surveillance. *Am J Epidemiol.* 1972; 96: 94–106.
10. Hamre D, Beem M: Virologic studies of acute respiratory disease in young adults. V. Coronavirus 229E infections during six years of surveillance. *Am J Epidemiol.* 1972; **96**: 94–106.
11. Treibel TA, Manisty C, Burton M. et al. COVID-19: PCR screening of asymptomatic healthcare workers at London hospital. *Lancet* 2020;18(1):462.

12. Department of Health and Social Care. Priority groups for coronavirus (COVID-19) vaccination: advice from the JCVI, 2 December 2020. Available from https://www.gov.uk/government/publications/priority-groups-for-coronavirus-covid-19-vaccination-advice-from-the-jcvi-2-december-2020/priority-groups-for-coronavirus-covid-19-vaccination-advice-from-the-jcvi-2-december-2020 DHSC: London, 2020 (accessed 7 December 2020).
13. Australian Government Department of Health and Aged Care. Australian influenza surveillance report. 2022. https://www1.health.gov.au/internet/main/publishing.nsf/Content/A7A060B878509419CA2588BF001207AA/$File/flu-12-2022.pdf
14. de Souza D, Cancho VG, Rodrigues J, Balakrishnan N. Bayesian cure rate models induced by frailty in survival analysis. *Stat Methods Med Res* 2017;26(5):2011-28
15. ChristoUKHSAr H. Jackson. Multi-State Models for Panel Data: The msm Package for R. *J Stat Soft* 2011;38:8.
16. NIHR, Payment guidance for researchers and professionals. NIHR, 2021.
