# Supplementary material for: Effect of second booster vaccinations and prior infection against SARS-CoV-2 in the UK SIREN healthcare worker cohort
Source: Lancet Reg Health Eur. 2023 Dec 14;36:100809. doi: 10.1016/j.lanepe.2023.100809 (PMC10727938; doi:10.1016/j.lanepe.2023.100809)
Supplement: SIREN Statistical Analysis Plan [file mmc3.pdf]

SIREN

SARS-CoV2 Immunity & Reinfection Evaluation

SIREN Study Statistical Analysis Plan

**Sections**	Date
**1. Original Statistical Analysis plan**<br>• As recorded in original study protocol	May 2020
**2. Vaccine Effectiveness Statistical Analysis plan**<br>• As recorded in Protocol Amendment December 2020	December 2020
**3. Key subsequent decisions made concerning statistical analysis methods**	
**3.1. First Interim analysis**	December 2020
**3.2. Interim analysis 2**	February 2021
**3.3. Interim analysis 3 (First Vaccine effectiveness analysis)**	February 2021
**3.4. Interim analysis 4**	December 2021
**3.5. Interim analysis 5**	March 2022
**3.6. Interim analysis 6**	March 2023
**Version 1**	03/08/2023

1. Original statistical analysis plan, May 2020

All enrolled participants will be included in analyses, which will account for clustering by research site. Analyses will be conducted after each 4-week period to inform the UK's response to the COVID-19 pandemic. Results will be available to all organisations involved in the research. The study follow-up period will end by default 12 months following the enrolment of the last participant, but by consensus of the study management group and funder may be terminated sooner if findings are sufficient.

There are no formal stopping rules for futility, utility or lack of power. The final decision to terminate the study will be made by Public Health England and Department for Health and Social Care.

Estimates of both cumulative incidence and incidence density in the seropositive and seronegative cohorts will be obtained using mixed effects models assuming counts of PCR positive have a negative binomial distribution, a log link function, and the natural logarithm of the total number of subjects or the total follow-up time use as an offset, respectively. Inclusion of a binary predictor indicating the sero-status of the cohort into this model will provide estimates of the incidence rate ratio. Sites will be incorporated as a random intercept to account for unmeasured, shared, site level factors. To account for a non-constant force of infection, calendar month will be incorporated as an additional random effect. An assessment of the role of factors such as age, gender, ethnicity in immunity will be explored by inclusion of interactions within the model between each and serological status.

While the above analytical approaches provide a "classical" person-years approach to prospective cohort analysis and provide familiar measures of association, it may be inadequate to assessment of immunity provided by seroconversion. As it is expected that seropositivity is likely to confer a degree of short to median term protection for a SARS-CoV-2 infection, multi-state and parametric cure rate models incorporating frailty will be employed. These "survival" type of models provide a more detailed assessment of factors associated with both short term and longer-term protection from infection, and how immunity may wane over time. Both mixture, explicitly assuming an immune and non-immune group, and non-mixture "cure rate" models will be assessed using information criterion to choose which provides a better fit to the observed data. Bayesian approaches to cure rate models with frailty as described by deSouza will be employed.[1]

Multi state models explicitly allowing those within an "immune" state to flow into a "susceptible" state as antibodies wane will also be employed. This framework can allow subjects to move from seronegative (susceptible) to seropositive (immune) when infected during the study period. An additional absorbing state will be used for those infected that died. It is also possible to introduce "misclassification" of state into the multi state model, providing an estimate of sensitivity to account for imperfect serological tests. Approaches like those proposed by Jackson will be employed.[2]

Procedure for Accounting for Missing, Unused, and Spurious Data: Analyses will be restricted to cases with antibody and PCR tests. The PCR test for virus is being used as a diagnostic test and hence has high performance. Sufficient sera will be obtained to re-run the immunological assays in case of initial assay failure. For similar reasons we do not anticipate that spurious data will be obtained.

Procedures for Reporting any Deviation(s) from the Original Statistical Plan: Deviations from the original statistical plan or the statistical analysis plan will be described and justified in the analysis reports.

2. Vaccine Effectiveness analysis plan, December 2020

Survival analysis will be used to estimate the hazard ratio in vaccinated compared to unvaccinated SIREN participants with VE = 1 – HR. A nested test negative case-control analysis will also be done with those swabbed but negative as the controls. If more than one vaccine is used vaccine effectiveness will be stratified by vaccine manufacturer. Vaccine effectiveness will also be stratified by baseline positivity (either PCR or antibody), age group (<50, >=50) and time since vaccination (three-month intervals and as a spline). Interaction with sex, ethnicity and risk group will be tested and, if significant, vaccine effectiveness will be stratified by these factors.

If the vaccine is rolled out over a very short period to HCWs with very high coverage, then the unvaccinated group will be small and probably an unusual subset. Even if coverage is not high those that do not get vaccinated when it is highly recommended may be different in ways that could lead to confounding. For example, those previously infected may not see the need for vaccination, or those not regularly working on site might miss vaccination. Those that perceive themselves as low risk of severe disease or with less patient contact may also be less likely to get the vaccine. Those not getting vaccinated may also be more likely to be those not providing regular swabs or blood samples. It will therefore be important to compare the vaccinated and unvaccinated cohorts to identify these potential biases. Using only those completing regular follow-up may help reduce such biases.

If coverage is very high and rapid then instead of vaccine effectiveness assessment it may be possible to do an impact assessment using a controlled interrupted time series approach in which COVID-19 incidence is compared over time in the HCW population to the general population (using external data) or between sites if vaccine introduction varies sufficiently by site. This can be done using Poisson or negative binomial regression.

3. Key subsequent decisions made concerning statistical analysis methods

3.1. First interim analysis (December 2020)

Time-varying explanatory variables introduced:

- Vaccine status not reliably available so a categorical variable was introduced for the weeks when the COVID-19 vaccination began (8th December 2020 to first week January 2021 to account for time to develop an antibody response)
- Binary categorical variable for alpha-variant emergence: this varied by region, and the cut-point of when over 50% of PCR-positive tests at Pillar-2 laboratories were SGTF positive.

Statistical modelling approach:

- Used logistic regression model with re-infection or not as outcome variable

3.2. Interim analysis 2 (February 2021)

- Adapted statistical modelling approach upon peer-reviewer comments to use a time-segmented Poisson regression model to better account for the changes in baseline risks of re-infection. This model gave equivalent results to the logistic regression model.

3.3. First Vaccine Effectiveness Analysis, Interim analysis 3 (March 2021)

Handling vaccine variable

- Time since vaccination segmented into 11 pre-specified vaccine intervals, using consistent time periods as national vaccine surveillance team, to calculate time since vaccination varying VE.

Statistical modelling approach

- Mixed effects (shared-frailty) Poisson regression model. Individual subject's follow-up time was split at each unique event time and categorised to fit as a count-time model using number of infections as the outcome variable and the logarithm of the person time as an offset.[3,4]

3.4. Interim analysis 4 (December 2021)

Handling vaccine variable(s)

- Larger number of periods used for post-vaccination intervals, particularly post dose-2 given available follow-up time/numbers to estimate time-varying VE.
- Two dose BNT162b2 vaccine categorised into short-interval (predominately 3-week inter vaccination interval) and long-interval (predominately 3-month inter vaccine interval) following UK policy change to delay delivery of dose 2 during study period. This was initiated to investigate whether interval between doses affected VE.

Handling those with a previous SARS-CoV-2 infection

- Sub-cohorts of those infected before vaccination, and those with no evidence of a previous infection before vaccination.
- Allowed participants to progress from negative to positive cohort after primary infection, ignoring the first 90 days after first PCR positive date, after which they

become at risk of reinfection (corresponding to SIREN reinfection definition of two PCR positives 90 days apart).

Modelling approach

- Cox regression model used, however the Poisson model outlined in 3.2 gave equivalent results
- The sub-cohort of those without a previous infection was used in the estimation of vaccine effectiveness in each post vaccination time category. The time to infection was based on calendar time, with follow-up beginning on the 7th December 2020 with those recruited post the 7th December 2020 having a delayed entry into the analysis.

3.5. Interim analysis 5 (March 2022)

Handling vaccine variable(s)

- Comparing waned dose 2 protection against dose 3

Handling variant period

- Follow-up time split by dominant variant, providing separate estimates of booster protection during Delta dominant period and Omicron dominant period

Modelling approach

- Cox regression model used

3.6. Interim analysis 6 (March 2023)

References

1. de Souza D, Cancho VG, Rodrigues J, Balakrishnan N. Bayesian cure rate models induced by frailty in survival analysis. Stat Methods Med Res 2017;26(5):2011-28
2. Jackson CH. Multi-State Models for Panel Data: The msm Package for R. J Stat Soft 2011; 38:8.
3. Holford TR, Life tables with concomitant information. Biometrics. 1976; 32: 587-597
4. Holford TR, The analysis of rates and of survivorship using log-linear models. Biometrics. 1980; 36: 299-305
